# The pleiotropic functions of intracellular hydrophobins in aerial hyphae and fungal spores

**DOI:** 10.1371/journal.pgen.1009924

**Published:** 2021-11-17

**Authors:** Feng Cai, Zheng Zhao, Renwei Gao, Peijie Chen, Mingyue Ding, Siqi Jiang, Zhifei Fu, Pingyong Xu, Komal Chenthamara, Qirong Shen, Günseli Bayram Akcapinar, Irina S. Druzhinina

**Affiliations:** 1 The Key Laboratory of Plant Immunity, Jiangsu Provincial Key Lab of Solid Organic Waste Utilization, Nanjing Agricultural University, Nanjing, China; 2 Fungal Genomics Laboratory (FungiG), Nanjing Agricultural University, Nanjing, China; 3 Institute of Chemical, Environmental and Bioscience Engineering (ICEBE), TU Wien, Vienna, Austria; 4 Key Laboratory of RNA Biology, Institute of Biophysics, Chinese Academy of Science, Beijing, China; 5 Department of Medical Biotechnology, Institute of Health Sciences, Acibadem Mehmet Ali Aydinlar University, Istanbul, Turkey; Oregon State University, UNITED STATES

## Abstract

Higher fungi can rapidly produce large numbers of spores suitable for aerial dispersal. The efficiency of the dispersal and spore resilience to abiotic stresses correlate with their hydrophobicity provided by the unique amphiphilic and superior surface-active proteins–hydrophobins (HFBs)–that self-assemble at hydrophobic/hydrophilic interfaces and thus modulate surface properties. Using the HFB-enriched mold *Trichoderma* (Hypocreales, Ascomycota) and the HFB-free yeast *Pichia pastoris* (Saccharomycetales, Ascomycota), we revealed that the rapid release of HFBs by aerial hyphae shortly prior to conidiation is associated with their intracellular accumulation in vacuoles and/or lipid-enriched organelles. The occasional internalization of the latter organelles in vacuoles can provide the hydrophobic/hydrophilic interface for the assembly of HFB layers and thus result in the formation of HFB-enriched vesicles and vacuolar multicisternal structures (VMSs) putatively lined up by HFBs. These HFB-enriched vesicles and VMSs can become fused in large tonoplast-like organelles or move to the periplasm for secretion. The tonoplast-like structures can contribute to the maintenance of turgor pressure in aerial hyphae supporting the erection of sporogenic structures (e.g., conidiophores) and provide intracellular force to squeeze out HFB-enriched vesicles and VMSs from the periplasm through the cell wall. We also show that the secretion of HFBs occurs prior to the conidiation and reveal that the even spore coating of HFBs deposited in the extracellular matrix requires microscopic water droplets that can be either guttated by the hyphae or obtained from the environment. Furthermore, we demonstrate that at least one HFB, HFB4 in *T*. *guizhouense*, is produced and secreted by wetted spores. We show that this protein possibly controls spore dormancy and contributes to the water sensing mechanism required for the detection of germination conditions. Thus, intracellular HFBs have a range of pleiotropic functions in aerial hyphae and spores and are essential for fungal development and fitness.

## Author summary

Hydrophobins (HFBs) are unique, non-toxic small fungal proteins with outstandingly high surface activity. They modulate surface properties by self-assembling at interfaces and have a multitude of applications for drug delivery, biosensors, and cosmetics. To explore the applied potential of HFBs, we investigated their functions in the naturally HFB-enriched *Trichoderma* spp. fungi (Ascomycota). We show that HFBs unexpectedly specifically accumulate inside aerial hyphae, where they associate with lipid-enriched organelles and putatively line up the vacuolar structures, which contribute to the structure and longevity of aerial mycelium. The maximum of HFB synthesis and secretion happens before the sporulation, providing a massive extracellular matrix suitable for the protective coating of rapidly produced spores. Furthermore, HFBs are involved in the water sensing mechanism of spores and thus are linked to the dormancy/germination switch. Our results reveal that HFBs have a broad range of intracellular functions and are essential for fungal development and fitness.

## Introduction

The hydrophobicity of the body surface is essential for the fungal lifestyle. Fungi feed by secreting digestive enzymes and subsequently absorbing dissolved small molecules from the surrounding substrate. This nutritional strategy requires a hydrophilic surface. However, nonmotile spores of higher fungi need to be hydrophobic because they are passively dispersed by air or water. Thus, for reproduction, fungi grow out of the substrate and form biofilms–aerial hyphae and hydrophobic sporogenic structures (e.g., fruiting bodies, sporangia, and conidiophores) and spores [1, 2] catching a usually short moment of environmental conditions suitable for the dispersal. The hydrophobicity of the spore or hyphal cell wall also influences their postdispersal biotic and abiotic interactions such as adhesion to substrates and their symbiotic partnerships [3, 4]. Therefore, the ability to adjust and modulate the hydrophobicity of the body surface is crucial for fungal lifestyle and autecology [1, 5].

One billion years of evolution of filamentous fungi [6] has resulted in molecular adaptations to the physicochemical challenges associated with their lifestyle. For example, higher filamentous fungi commonly secrete hydrophobins (HFBs), which are unique small (usually < 20 kDa) amphiphilic and highly surface-active proteins [7–9] that are characterized by the presence of eight cysteine (Cys) residues, four of which form two Cys—Cys pairs. HFBs are believed to be initially secreted in a soluble form and then can spontaneously localize at the hydrophilic/hydrophobic interface, where they assemble into amphipathic layers [10] of varying solubility [7]. These layers significantly decrease the interfacial tension, thus allowing the hyphae to breach the liquid surface and grow into the air by forming buoyant colonies. Fruiting bodies, aerial hyphae, and spores are also largely coated by HFBs to reduce wetting, provide resilience to environmental stresses [1, 11], promote the adhesion of spores and hyphae to hydrophobic surfaces or interactions with symbiotic partners [4], and influence growth and development [12–14].

Since fungi frequently need to produce large amounts of spores in a short period of time [15] and spores require a HFB coat, they need to rapidly secrete large amounts of these proteins [1] that have to be synthesized by sporulating and most commonly aerial hyphae. In molds, aerial hyphae are ephemeral structures [5] that are vulnerable to the environmental stressors in an open air and quickly become aged because they are deprived of nutrient and water absorption. Thus, aerial hyphae are frequently exposed to drought, UV radiation, and mechanical damage by rain, wind, or animals, and they may also need to grow against gravity. The secretion of HFBs during the formation of sporangia and spores is unlikely, as conidiogenesis is an energy-consuming task [15] that leaves few resources for the synthesis of accessory proteins required in large amounts. Under such circumstances, the mechanisms by which HFBs can be abundantly secreted are not known.

Most fungi have only a few HFB-encoding genes (JGI Mycocosm, June 2020), although the genomes of some extremophilic species (*Wallemia ichthyophaga* [16]) or mycorrhizal mushrooms [17] contain exceptionally rich arsenals of HFBs. In Ascomycota molds, the genomes of fungi from the order Hypocreales have an extreme variety of HFB-encoding genes [18]. Among them, the genus *Trichoderma* exhibits the highest number and diversity of HFBs, which were reported as the main genomic hallmarks of these fungi [18, 19]. The number of HFBs in individual *Trichoderma* species can range from seven in *T*. *reesei* to 16 in *T. atroviride* [18]. Contrary to HFBs in mushrooms and some airborne *Ascomycota* such as species of *Penicillium* and *Aspergillus*, most *Trichoderma* HFBs do not form rodlets or amyloids and are soluble in organic solvents and detergents [7, 20, 21]. An ecological genetic study of the two *Trichoderma* species, *T*. *harzianum* and *T*. *guizhouense*, revealed that HFB4 on the spore surface can control the preferential dispersal mode and spore survival [1]. However, to date, the biological or ecological role of the enrichment of HFBs in *Trichoderma* genomes has not been understood [18]. In the present study, we investigated the localization, secretion mechanisms, and function of HFBs during the development of two commonly occurring cosmopolitan sibling *Trichoderma* species, *T*. *harzianum* and *T. guizhouense* [1, 22, 23]. The intracellular localization of HFBs was also investigated in the recombinant methylotrophic yeast *Pichia pastoris*, which has no HFB-encoding genes in its genome [24].

## Results

Our previous ecological genetic investigation revealed that HFB4 and HFB10 are highly significant for the fitness of the two sibling *Trichoderma* species [1]. In particular, HFB4 contributes to the anemophilous dispersal of *T*. *guizhouense* and evolves under positive natural selection pressure in *T*. *harzianum*, where it is likely associated with the affinity to pluviophilous (rain droplets) spore dispersal. The genomes of *T*. *harzianum* CBS 226.95 (Th) and *T*. *guizhouense* NJAU 4742 (Tg) contain eleven and nine HFB-encoding genes, respectively [1]. All these proteins have the characteristic arrangement of eight Cys residues and possess signal peptides, which indicate their affinity to the conventional secretory pathway through endoplasmic reticulum (ER) and extracellular vesicle trafficking.

To address the function of these genes during different stages of the life cycle, we first tested their expression at (i) spore germination, (ii) in submerged trophic hyphae, (iii) in aerial hyphae before conidiation, and (iv) in aerial conidiating mycelium (Table 1). In both species, the highest transcription level of *hfb* genes was recorded during the life stages that included the formation of aerial hyphae. A principal component analysis (PCA) of the expression profile of *hfb* genes confirmed the strong involvement of *hfb4* and *hfb10* in the development of aerial mycelium and spores of Tg and Th and a minor role of *hfb3* (only noticeable at the conidiophore formation stage), followed by *hfb2* (S1 Fig). The remaining genes of both species were silent at these developmental stages and are probably required either for sexual reproduction or biotic interactions. Consequently, we then constructed a library of *hfb*-deficient, *hfb*-overexpressing and fluorescently labeled mutants for the above four genes in both species (Table 2). As fluorescent protein tags may potentially influence the properties of HFBs [25], we predicted the properties of the fusion proteins using *in silico* 3D modeling and molecular dynamics (MD) simulation analysis for their behavior in water (S2 Fig). Based on this analysis, we selected the two fluorescent proteins (mRFP and YFP) and designed most optimal fusion constructs that correspond to the exposed position of the HFB hydrophobic patches with the highest probability (S2 Fig). To verify that off-site genetic transformation did not cause phenotypic differences, each genotype was represented by multiple mutants (two to five) and characterized based on at least a pair. Furthermore, we produced reverse complemented mutants in which fluorescently tagged and untagged HFB-encoding genes were reintroduced to the corresponding deletion mutants and compared their properties. The phenotypes of the mutants are shown in the S3 Fig. Briefly and as expected, the deletion of either *hfb4* or *hfb10* or both genes resulted in reduced conidiation and a “wetted hyphae” phenotype (impaired surface hydrophobicity) in both species [1]. The deletion of *hfb2* did not result in phenotypic alterations.

**Table 1:** Expression of *hfb*-encoding genes during four stages of *Trichoderma* development as quantified by qPCR in relation to the housekeeping gene *tef1* [1]. The complementary principal component analysis is shown in S1 Fig.

Parental strain NCBI Genome ID	*hfb* gene name	NCBI Accession Numbers		Relative gene expression							
				Spore germination		Trophic hyphae		Aerial hyphae		Conidiation	
				Fold expression	SD	Fold expression	SD	Fold expression	SD	Fold expression	SD
***T*. *harzianum* CBS 226.95 (MBGI00000000.1)**	-	PTB60449	MF527127	**0**		**0**		**0**		**0**	
	*hfb3*	PTB52129	MF527128	**0**		**7.02E-05**	*2*.*9E-05*	**4.93E-01**	*1*.*6E-01*	**6.14E-02**	*7*.*4E-03*
	***hfb4***	PTB58174	MF527129	**4.67E-05**	*9*.*0E-06*	**3.11E-04**	*8*.*9E-06*	**3.47E+00**	*3*.*7E-02*	**1.27E+00**	*2*.*2E-01*
	*hfb5*	PTB53925	MF527130	**0**		**0**		**0**		**0**	
	*hfb6*	PTB60167	MF527131	**0**		**0**		**0**		**0**	
	***hfb2*** *	PTB60601	MF527132	**0**		**1.08E-01**	*1*.*9E-02*	**1.06E+00**	*2*.*1E-01*	**1.75E-01**	*2*.*3E-02*
	*hfb9a*	PTB50855	MF527133	**0**		**9.43E-06**	*4*.*3E-06*	**2.37E-05**	*6*.*7E-06*	**1.73E-03**	*3*.*6E-04*
	*hfb9b*	PTB48391	MF527134	**0**		**6.62E-06**	*1*.*8E-06*	**2.26E-04**	*1*.*3E-04*	**5.95E-04**	*6*.*1E-05*
	***hfb10***	PTB48206	MF527135	**2.85E-01**	*2*.*5E-02*	**1.47E-01**	*4*.*6E-02*	**2.14E+00**	*3*.*0E-01*	**7.66E-01**	*1*.*1E-01*
	-	PTB49111	MF527136	**0**		**8.54E-04**	*7*.*9E-05*	**5.23E-04**	*1*.*1E-04*	**5.39E-05**	*1*.*4E-05*
	-	PTB56946	MF527138	**0**		**0**		**0**		**0**	
***T*. *guizhouense* NJAU 4742 (LVVK00000000.1)**	*hfb3*	OPB45549	MF527117	**0**		**9.68E-03**	*1*.*1E-03*	**3.19E-01**	*4*.*5E-02*	**1.59E-02**	*3*.*8E-03*
	***hfb4***	OPB37525	MF527118	**1.58E-04**	*6*.*2E-05*	**1.12E-02**	*2*.*5E-03*	**1.30E+00**	*1*.*6E-01*	**7.36E-01**	*1*.*7E-01*
	*hfb5*	n.a.	MF527119	**0**		**1.22E-04**	*4*.*1E-05*	**4.88E-03**	*5*.*8E-04*	**3.18E-02**	*5*.*2E-03*
	*hfb6*	OPB38878	MF527120	**0**		**0**		**0**		**0**	
	***hfb2*** *	OPB38530	MF527121	**0**		**1.87E-01**	*1*.*5E-02*	**7.66E-01**	*1*.*2E-01*	**2.83E-02**	*1*.*0E-02*
	*hfb9a*	OPB40515	MF527122	**0**		**6.32E-06**	*1*.*4E-06*	**6.58E-06**	*3*.*6E-06*	**3.69E-04**	*9*.*0E-05*
	*hfb9b*	OPB44528	MF527123	**0**		**3.39E-03**	*6*.*9E-04*	**7.15E-03**	*1*.*1E-03*	**2.35E-03**	*3*.*7E-04*
	***hfb10***	OPB44696	MF527124	**1.90E+00**	*8*.*0E-01*	**1.31E+01**	*3*.*7E-01*	**1.42E+01**	*2*.*7E+00*	**2.34E+00**	*2*.*2E-01*
	-	OPB45278	MF527125	**0**		**3.53E-06**	*2*.*2E-06*	**1.73E-05**	*5*.*9E-06*	**0.0019**	*2*.*6E-04*

*hfb2, representing *hfb2* sensu lato including *hfb7* [26, 27].

**Table 2:** Strains used in this study.

Strain description	TUCIM ID	Strain name	TUCIM ID	Strain name
	*Trichoderma guizhouense*		*Trichoderma harzianum*	
Wild type	4742	**NJAU 4742**	916	**CBS 226.95**
Fluorescently labeled mutants	7436	_**Tg**_ ***hfb4*::*mrfp*-_Tg_*hfb10*::*yfp***	7422	_**Th**_ ***hfb4*::*yfp-*_Th_*hfb10*::*mrfp***
*hfb4*-deleted mutant	7435	_**Tg**_ **Δ*hfb4*-1**	7421	_**Th**_ **Δ*hfb4*-3**
	7434	_**Tg**_ **Δ*hfb4*-4**	7420	_**Th**_ **Δ*hfb4*-11**
*hfb10*-deleted mutant	7431	_**Tg**_ **Δ*hfb10*-2**	7417	_**Th**_ **Δ*hfb10*-2**
	7430	_**Tg**_ **Δ*hfb10*-3**	7416	_**Th**_ **Δ*hfb10*-17**
*hfb4* and *hfb10* double-deleted mutant	7426	_**Tg**_ **Δ*hfb4*-_Tg_Δ*hfb10*-2**	7413	_**Th**_ **Δ*hfb4*-_Th_Δ*hfb10*-27**
	7427	_**Tg**_ **Δ*hfb4*-_Tg_Δ*hfb*10-11**	7412	_**Th**_ **Δ*hfb4*-_Th_Δ*hfb10*-30**
*hfb2*-deleted mutant	7442	_**Tg**_ **Δ*hfb2*-1**	8005	_**Th**_ **Δ*hfb2*-1**
	7441	_**Tg**_ **Δ*hfb2*-17**	8006	_**Th**_ **Δ*hfb2*-2**
*hfb4*-overexpressing mutant	7433	_**Tg**_ **OE*hfb4*-6**	7419	_**Th**_ **OE*hfb4*-11**
	7432	_**Tg**_ **OE*hfb4*-13**	7418	_**Th**_ **OE*hfb4*-13**
*hfb10*-overexpressing mutant	7429	_**Tg**_ **OE*hfb10*-9**	7415	_**Th**_ **OE*hfb10*-1**
	7428	_**Tg**_ **OE*hfb10*-10**	7414	_**Th**_ **OE*hfb10*-6**
*mrfp*-labeled *hfb2*-overexpressing mutant	7439	_**Tg**_ **OE*hfb2*::*mrfp*-23**	-	
	7438	_**Tg**_ **OE*hfb2*::*mrfp*-24**		
*mrfp*-labeled *hfb3* strain	10530	_**Tg**_ ***hfb3*::*mrfp***		
*mrfp*-labeled *hfb4* strain	7424	_**Tg**_ ***hfb4*::*mrfp***		
*rab7*-deleted mutant	8001	_**Tg**_ **Δ*rab7*-30**		
	8002	_**Tg**_ **Δ*rab7*-55**		
*mrfp*-labeled *hfb4-* and *rab7-*deleted mutant	8003	_**Tg**_ ***hfb4*::*mrfp*-_Tg_Δ*rab7*-11**		
	8004	_**Tg**_ ***hfb4*::*mrfp*-_Tg_Δ*rab7*-17**		
*hfb4*-complementary mutant to *hfb4-*deleted mutant	10522 10523	_**Tg**_ **Δ*hfb4*::*hfb4*-10** _**Tg**_ **Δ*hfb4*::*hfb4*-15**		
*hfb4*::*mrfp*-complementary mutant to *hfb4-*deleted mutant	10524 10525	_**Tg**_ **Δ*hfb4*::*hfb4*::*mrfp*-3** _**Tg**_ **Δ*hfb4*::*hfb4*::*mrfp* -5**		
*hfb10*-complementary mutant to *hfb10-*deleted mutant	10526 10527	_**Tg**_ **Δ*hfb10*::*hfb10*-11** _**Tg**_ **Δ*hfb10*::*hfb10-*12**		
*hfb10*::*yfp*-complementary mutant to *hfb10-*deleted mutant	10528 10529	_**Tg**_ **Δ*hfb10*::*hfb10*::*yfp*-1** _**Tg**_ **Δ*hfb10*::*hfb10*::*yfp*-7**		
*mrfp-*expressing strain under the control of the *hfb4* promoter	8000	_**Tg**_ **P**_***hfb4***_ **::*mrfp***		
***Pichia pastoris***				
Wild type			8007	**KM71H**
KM71H strain transformed with the empty vector of pPICZαA			6633	_**Pp**_ **vec**
*hfb4-*expressing strain under control of the *P*_aox1_ promoter with the signal peptide of α mating factor from *Saccharomyces cerevisiae*			6616	_**Pp**_ **P**_***aox1***_ ***S***_**α**_ **::_Tg_*hfb4***
*hfb2-*expressing strain under control of the *P*_aox1_ promoter with the signal peptide of α mating factor from *S*. *cerevisiae*			6617	_**Pp**_ **P**_***aox1***_ **S**_**α**_ **::_Tg_*hfb2***
*gfpuv-*labeled *hfb4-*expressing strain under control of the *P*_aox1_ promoter with the signal peptide of α mating factor from *S*. *cerevisiae*			6625	_**Pp**_ **P**_***aox1***_ **S**_**α**_ **::_Tg_*hfb4*::*gfpuv***
*mrfp-*labeled *hfb2-*expressing strain under control of the *P*_aox1_ promoter with the signal peptide of α mating factor from *S*. *cerevisiae*			6626	_**Pp**_ **P**_***aox1***_ **S**_**α**_ **::_Tg_*hfb2*::*mrfp***

TUCIM, the TU Collection of Industrial Microorganisms, Vienna, Austria.

### Hydrophobins massively accumulate inside aerial hyphae

**Fig 1:**
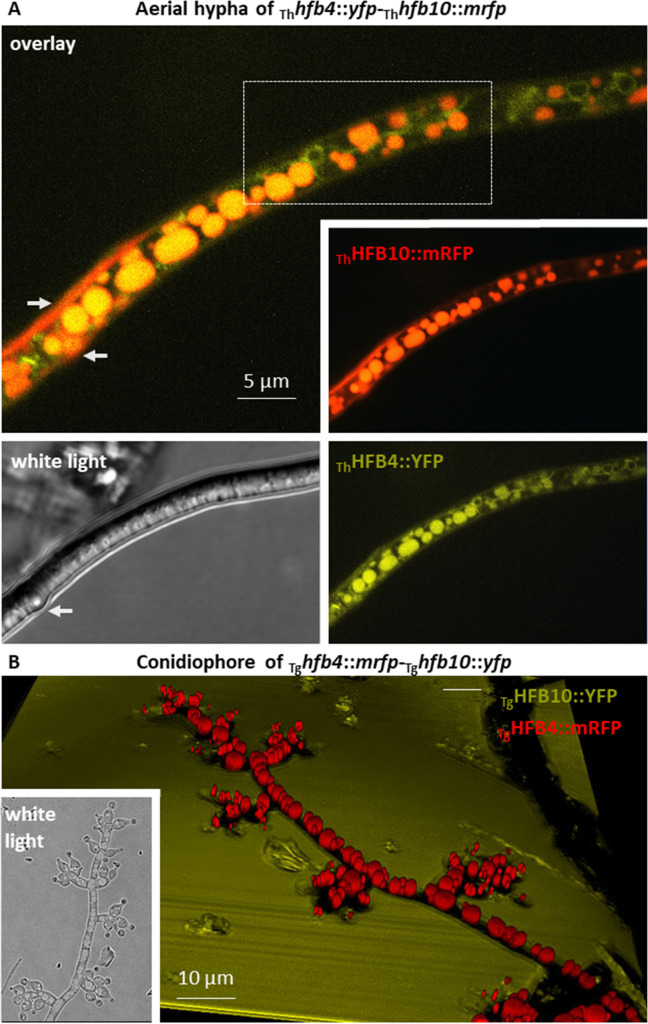
*In vivo* visualization of intracellular accumulation of fluorescently labeled hydrophobins in aerial hyphae and conidiophores of *Trichoderma* spp. (A) Aerial hyphae of *T*. *harzianum*_Th_*hfb4*::*yfp*-_Th_*hfb10*::*mrfp* accumulating HFB4::YFP and HFB10::mRFP in putative conventional and tubular vacuoles (dashed area) and the periplasm (arrows). (B) A 3D reconstruction of fluorescent overlay images of the mature conidiophore of *T*. *guizhouense* producing HFB4::mRFP and HFB10::YFP. The white light image of the same conidiophore is shown in an inset.

*In vivo* epifluorescence microscopy of mutant strains expressing fluorescently labeled HFBs (fused with mRFP and YFP in different combinations in the two species) unexpectedly revealed that the formation of aerial hyphae was accompanied by massive intracellular accumulation of HFB4 and HFB10, respectively, which were stored in different types of membrane-bound vesicles and in a periplasmic or cell wall location (Fig 1). Additionally, HFBs were not visible in the cytoplasm and, also contrary to expectations, HFB4 and HFB10 were not associated with the cell wall. Even though both proteins were also detected extracellularly, HFB4 had a higher affinity for intracellular accumulation in stalk cells of conidiophores and phialides (sporogenic cells on conidiophores) (Figs 1 and S4) than HFB10. In contrast, HFB10 had a higher affinity than HFB4 to solid/liquid interfaces on the microscopy glass slide outside the cells (Fig 1). Another protein that was possible to visualize was HFB3 that also showed intracellular accumulation and was also visible on the surface of phialides specifically at collarettes (S5 Fig). As *hfb3* had very low expression levels (Table 1) and consequently HFB3::mRFP was hardly visible *in vivo*, this protein did not contribute to the subsequent study.

The selective affinity of HFB4 and HFB10 to conidiophores (S4 Fig) suggested that their intracellular localization was linked to their function rather than caused by the potential “utilization” of an alien fusion construct in vacuoles. Nevertheless, to test the role of the fluorescent tag, we expressed mRFP (without HFBs) using the promoter and signal peptide of _Tg_HFB4 (_Tg_P_*hfb4*_::*mrfp*). The resulting mRFP protein was secreted into the medium and was not retained in hyphae (S6 Fig). Thus, the intracellular localization of fusion proteins is not associated with the fluorescent tag.

To test whether this behavior of HFB peptides would occur in other fungi, we overexpressed *Trichoderma* HFB-encoding genes in *Komagataella pastoris* (Saccharomycetales, syn. *Pichia pastoris*), which has no *hfb* genes in its genome [24]. The resulting mutants are listed in Table 2. Despite the presence of the signal prepropeptide of the α-mating factor from *Saccharomyces cerevisiae*, *P*. *pastoris* cells also massively accumulated HFBs intracellularly (S7 Fig). Together, these results indicate that the intracellular localization of fluorescently tagged HFBs in vesicle-like organelles and in the periplasm is linked to the properties of HFBs and is not led by the signal peptide or a tag.

### Internalization of putative HFB-enriched organelles in vacuoles and the formation of vacuolar multicisternal structures

The morphology of HFB-containing organelles resembled vacuoles and lipid bodies (Fig 1).

**Fig 2:**
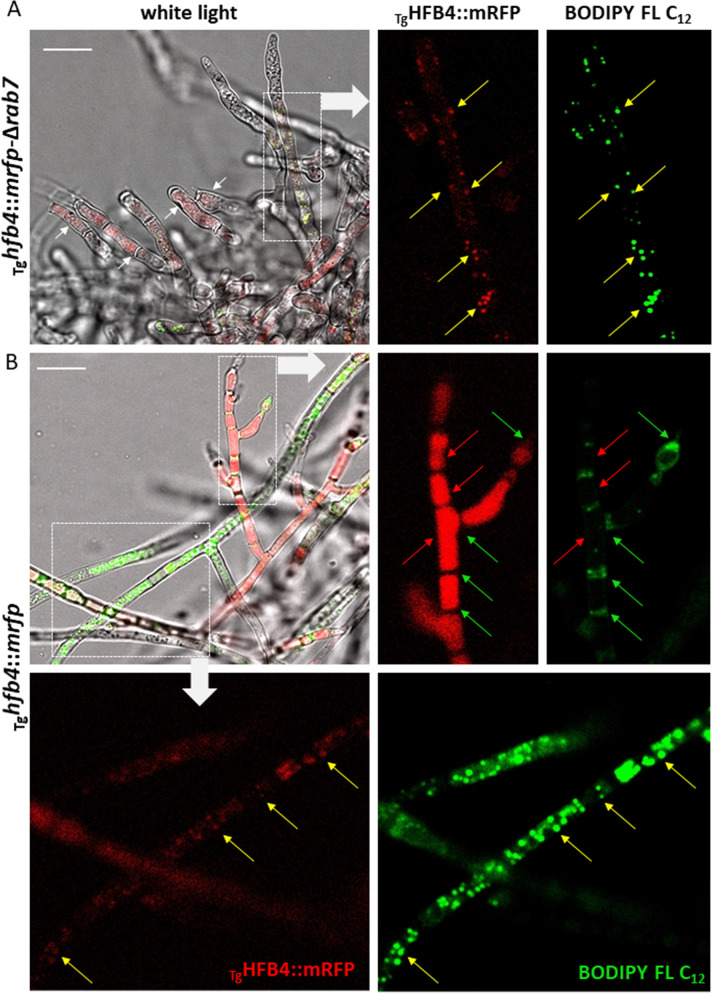
Accumulation of HFBs in vacuoles and lipid-enriched organelles. (A) Fluorescent images of the phospholipid-specific stained [using a green fluorescent fatty acid (BODIPY FL C_12_)] aerial hyphal tips of the *Trichoderma* mutant producing HFB4::mRFP but lacking the key protein for the cytoplasm-to-vacuole sorting pathway, RAB7 (homologous to YPT7 in *S*. *cerevisiae*). Note, the mutant forms almost no aerial hyphae. (B) Fluorescent images of the phospholipid-specific stained (using BODIPY FL C_12_) aerial hyphae of the Tg mutant producing _Tg_HFB4::mRFP. The pairs of red and green fluorescent images correspond to framed areas showing the localization of the fluorescently labeled HFBs (red arrows) and phospholipids (green arrows) or both (yellow arrows). Scale bar = 10 μm.

To test for the vacuolar location of HFBs, we used Tg and deleted the gene encoding endosomal RAB7 (OPB37336 in Tg), which is a small GTPase required for the late steps of multiple vacuole delivery pathways [28] (homologs include *ypt7* in *Saccharomyces cerevisiae* [29] and *avaA* in *Aspergillus nidulans* [28]), in Tg and in mutants expressing HFB4::mRFP (_Tg_*hfb4*::*mrfp* strain). The mutants _Tg_Δ*rab7* and _Tg_*hfb4*::*mrfp*-_Tg_Δ*rab7* showed strongly reduced growth with abolished ability to form aerial hyphae. They were highly hydrophilic, did not produce conidiophores or conidia and had no conventional large vacuoles (Figs 2A and S8). The cytoplasm of the _Tg_*hfb4*::*mrfp*-_Tg_Δ*rab7* strain was enriched in small HFB4::mRFP-containing vesicles (heterogeneous appearance of cytoplasm) but lacked large HFB-containing organelles as in Fig 1. Thus, the analysis of RAB7-deficient mutants confirmed that vacuole integrity is important for the formation of HFB-enriched organelles.

To test whether the other type of HFB-containing organelles observed in Fig 1 corresponded to lipid bodies, we applied differential phospholipid stain, BODIPY FL C_12_ (Thermo Fischer Scientific, USA), to the _Tg_*hfb4*::*mrfp* and _Tg_*hfb4*::*mrfp-*_Tg_Δ*rab7* mutants (Fig 2A). These results revealed cases of separate and overlapping localizations of HFB4 and lipids. In particular, this assay indicated that in lipid-rich aerial hyphae lacking large vacuoles, HFB4 was localized in lipid-enriched organelles, while in vacuolized cells, such mature conidiophores, HFB4 was preferentially detected in large vacuoles (Fig 2B). In the absence of integer vacuoles in the _Tg_Δ*rab7* strain, HFB4 could be detected in small vesicles and lipid-enriched organelles (Fig 2). The same investigation in spores reveled rather separate localization of HFB4 and lipids (S9 Fig) suggesting the different intracellular behavior of these proteins in spores and sporogenic structures.

**Fig 3:**
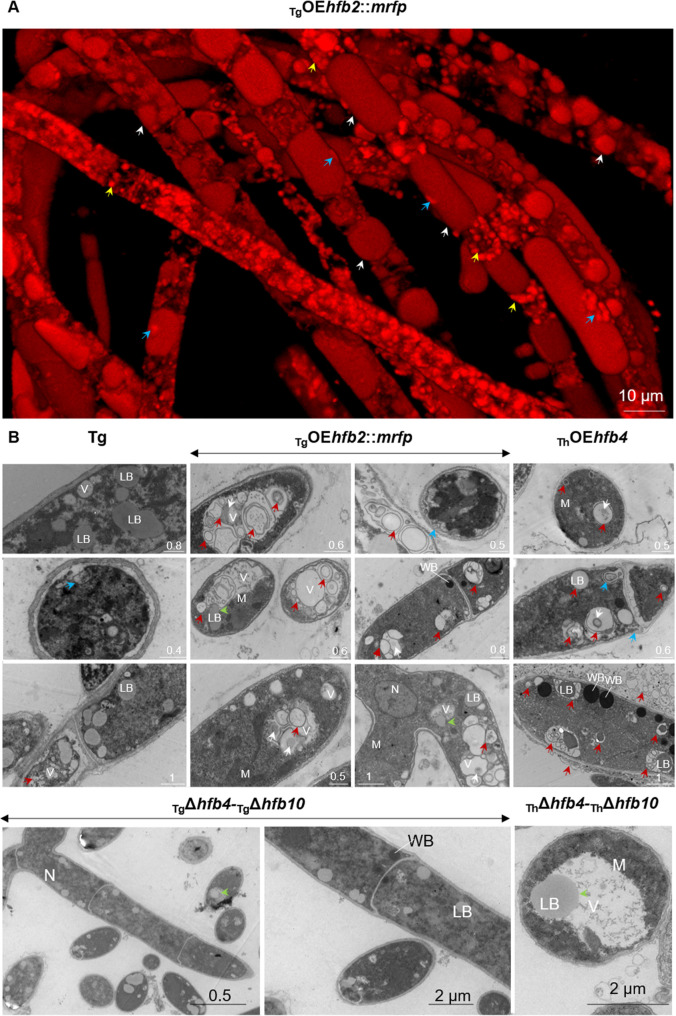
Ultrastructure of *Trichoderma* hyphae enriched in HFBs. (A) Aerial hyphae of *T*. *guizhouense* producing HFB2::mRFP under the control of the strong constitutive promoter of the *cdna1* gene from *T*. *reesei* QM6a (See Materials and Methods for details). White and yellow arrows point to the different types of organelles or vesicles accumulating HFB2::mRFP. The blue arrow indicates a putative site of organelle rupture or periplasmic accumulation. S1 Video contains a video corresponding to this image that provides more details. (B) Representative TEM micrographs showing the hyphal ultrastructure of strains including the wild-type strain of *T*. *guizhouense* (Tg), HFB2::mRFP-overexpressing strains of Tg [as on (A)], HFB4-overexpressing *T*. *harzianum* (Th), or hyphae of Tg and Th lacking HFB4 and HFB10. All images of the aerial hyphae were obtained from a two-day-old colony. V–vacuole, LB–putative lipid body, N–nucleus, M–mitochondrion, and WB–Woronin body. Red arrows point to putative HFB vesicles in vacuolar multicisternal structures (VMSs) and “matryoshka” structures. Blue arrows indicate the release of VMS content to the periplasm [see blue arrows in (A)]. Green arrows highlight the putative internalization events of LBs to Vs. Representative images were selected from a total of 695 images obtained for Tg (361) and Th (334). Samples for TEM were prepared with at least two mutants and 30 images studied per genotype. Mutants are listed in Table 2. Enlarged TEM images are available from the S11 Fig.

Another mean of investigating the *Trichoderma* hyphae accumulating HFBs is the ultrastructure analysis using transmission electron microscopy (TEM). In this case, our model strains were the visibly pink _Tg_OE*hfb2*::*mrfp* mutants (S10 Fig) because they constitutively produced a fluorescently tagged HFB2 and thus secured the presence of HFB-overloaded cells in the TEM sample. However all other strains were also analyzed. The fluorescent microscopic analysis of _Tg_OE*hfb2*::*mrfp* mutants revealed that aerial hyphae massively accumulate and tolerate a large quantity of HFB2 (Fig 3A; see the corresponding video in S1 Video). Interestingly, the colonies of these mutants appeared healthy despite of the visibly heavy intracellular load of a surface-active protein (S10 Fig). The TEM analysis of the aerial hyphae of the parental and HFB-overexpressing strains compared with those of the deletion strains revealed numerous vacuolar multicisternal structures (VMSs) associated with the high production of HFBs (Fig 3B). VMSs were characterized by a “matryoshka” of electron-dense membranes or films, and this result was found with the three HFBs studied (HFB4, HFB10, and HFB2) in both species. The matrix of the VMSs was almost electron transparent, with frequent inclusions of various sizes and electron densities (Figs 3 and S11).

The lipid-enriched organelles in HFB-overproducing cells were relatively more electron dense than those in the cells of the wild type and strains lacking HFBs, suggesting differences in their chemical composition, such as possible protein enrichment (*see above*). Multiple detections of putative internalization of lipid-enriched organelles in vacuoles (Fig 3B) suggested that this process could be important for VMSs and “matryoshka” formation. The hyphae of mutants lacking HFBs (in particular, _Tg_Δ*hfb4*- _Tg_Δ*hfb10* or _Th_Δ*hfb4*-_Th_Δ*hfb10*, Fig 3B) contained putative lipid bodies and vacuoles but were deprived of VMSs. Notably, the periplasm volume appeared to be essentially smaller in mutants lacking HFB4 and HFB10 than in the parental and HFB-overexpressing strains (Figs 3B and S11). At several sites, the release of VMS content into the periplasmic space was imaged (Figs 3B, also *see* S16 *below*), indicating that HFB secretion may go through the initial localization of HFB-enriched vesicles and VMSs in periplasm.

The ultrastructure of HFB-overproducing *P*. *pastoris* cells resembled that of *Trichoderma* with abundant VMSs also having the characteristic “matryoshka” appearances except that VMSs were not linked to the periplasm (S7 Fig). Similar to *Trichoderma*, multiple events of putative lipid bodies internalized in vacuoles were also observed in *P*. *pastoris*. Surprisingly, in this yeast, the release of HFBs occurred by the bursting of cells overloaded with intracellular HFBs. However, this finding is in agreement with the fact that *P*. *pastoris* is initially an HFB-free organism and thus has no mechanism for their secretion (S7 Fig).

Because VMS membranes seen in TEM micrographs can originate from the endoplasmic reticulum (ER) as ER whorls [30] and the massive accumulation of membranous structures in vacuoles resembles autophagy morphology [31, 32], we tested the expression of autophagy-related genes. The results showed no significant upregulation of autophagy marker genes associated with high HFBs accumulation (aerial hyphae or constitutive expression) (S12 Fig). Similarly, the accumulation of HFB-enriched VMSs did not cause any considerable upregulation of autophagy marker genes in *P*. *pastoris* (S12 Fig).

Taken together, the results of the above analyses indicated that in aerial hyphae, HFBs first accumulated in lipid-enriched organelles that occasionally were internalized in vacuoles and thus likely gave rise to VMSs. Our results allow us to conclude that in VMSs, HFBs self-assembled in the hydrophobic/hydrophilic interface created during the contact of the lipids with the vacuolar content. This can led to the lining up of VMS membranes by HFBs and/or the formation of HFB vesicles. Such process strongly resembles the formation of bilayer HFB1 vesicles reported by Hähl et al. in a cell-free oil emulsion in water and other liquids [33]. In some *Trichoderma* cells, in particular in _Tg_OE*hfb2*::*mrfp* mutants, several VMSs containing HFB vesicles fused and formed gigantic central tonoplast-like structures, as observed in Fig 3A and S1 Video. The release of VMSs with visually stable HFB microvesicles to the periplasm (Fig 3) can be associated with HFB secretion in aerial hyphae (*see below*).

### HFBs can aid conidiophore erection by contributing to turgor pressure in aerial hyphae

**Fig 4:**
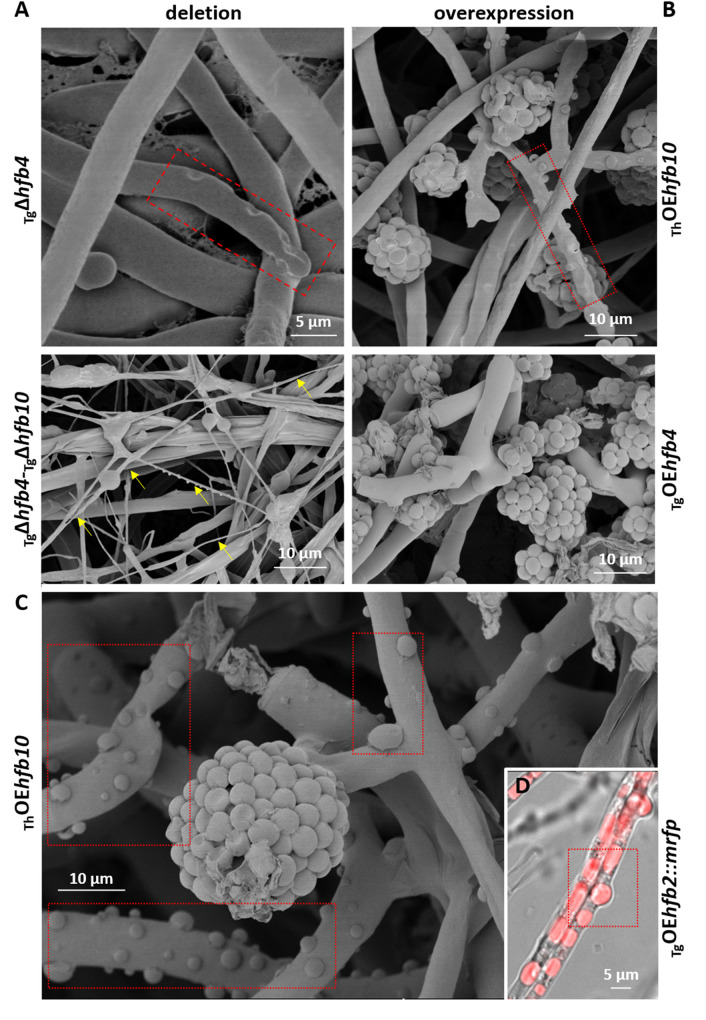
Morphology of aerial hyphae formed by HFB-deficient and HFB-overexpressing mutants of *Trichoderma* spp. suggests alterations in turgor pressure. (A) Cryo-SEM micrographs of aerial mycelium of HFB-deficient *Trichoderma* cultures grown on PDA for 11 d. The yellow arrows point a "spaghetti with mozzarella" phenotype that is made of remains of burst hyphae. (B-C) Cryo-SEM micrographs of aerial mycelium of HFB-overexpressing *Trichoderma* cultures grown on PDA for 28 d. (D) An image from epifluorescence microscopy showing the HFB-rich content of protrusions corresponding to those shown in (B) and (C). The red boxes in (A)–(B) and (C)–(D) highlight indentations or herniations corresponding to putatively reduced or increased intracellular turgor pressure, respectively. Representative images were selected from a total of 523 images obtained for Tg and Th. Samples for SEM were prepared with at least two mutants and 30 images studied per genotype. Mutants are listed in Table 2.

The deletion and overexpression of HFBs influenced the structure of mature aerial hyphae (Fig 4). A cryo-scanning electron microscopy (cryo-SEM) analysis of strains lacking either HFB4 or HFB10 or both revealed multiple large indentations (0.3–1 μm in diameter) on the surface of aerial hyphae. It was also observed on the hyphal tips (Fig 4A), where the turgor pressure is expected to be the highest [34]. In contrast, HFB-overexpressing strains had a visually inflated appearance (Fig 4B and 4C). Moreover, conidiophores and aerial hyphae (that have high expression of HFBs) of both species had abundant herniations (protrusions) of 0.3 to 1.3 μm in diameter filled with HFB-enriched vesicles. This morphology was absent in HFB-deletion strains (Fig 4) but frequently documented for different wild-types strains of *Trichoderma* (S13 Fig).

Herniations and the multiple observed cases of cell wall rupture and cell bursting (Figs 4 and 3A) suggest that HFBs are linked to the regulation of intracellular turgor pressure in aerial hyphae. Turgor pressure is the internal force that pushes the cytoplasm against the cell wall [34]. Thus, the indentations and herniations observed in HFB mutants could develop due to changes in the cell wall structure, as HFBs were reported to affect this organelle [35]. However, analysis of the cell wall ultrastructure did not reveal any visible effect of HFBs on the hyphal cell wall (S14 Fig) except that for the older hyphae of parental or HFB-overexpressing strains had a thick extracellular matrix that did not explain the observed indentations or herniations. Thus, the HFB-dependent phenotype was not linked to the functionality of the cell wall.

The elevated turgor pressure should influence the development of the hyphae in the conditions of the osmotic stress [36]. To test this, we cultivated Tg, deletion mutant (_Tg_*Δhfb4*-_Tg_Δ*hfb10*), and the HFB-overexpressing strain (_Tg_OE*hfb2*::*mrfp*) in 0.75 M, 1M, and 1.5 M solutions of NaCl, 1 M and 2 M sucrose, and 2 M glycerol (S15 Fig). These experiments reveled the outstanding resistance of the HFB2::mRFP overexpressing strain to the conditions of osmotic stress and showed no difference between Tg and the deletion strain due to the downregulation of HFB-encoding genes in submerged culture (Table 1). This result supports the above hypothesis on the putative role of intracellular HFBs in the maintenance of turgor pressure.

In aerial hyphae, which are usually hydrophobic and therefore deprived of water influx and less influenced by osmosis, turgor pressure can be maintained by the formation of a large central vacuole (= tonoplast) [37]. The results above suggest that besides the vacuole integrity, HFB vesicles can contribute to the formation and mechanical stability of such organelles. In line with this hypothesis, we observed that at the end of the life span of aerial mycelium (10–12 days of cultivation on PDA at room temperature and 12 h light cycle), the aerial parts of the colonies of both species were degraded, and vital hyphae were maintained only adjacent to conidiophores (Fig 4). At this stage, the strains lacking the major HFBs had almost no intact aerial hyphae because most of them had already burst, resulting in a "spaghetti with mozzarella" cryo-SEM phenotype (Fig 4A), where regular hyphae were intermixed with thin hyphal debris (putatively empty cell walls).

In summary, we speculate that HFBs can line up interface in VMSs and thus form either single HFB vesicles or large aggregates of such vesicles or tonoplast-like structures, contributing to the development of turgor pressure in aerial hyphae, including conidiophores, in a manner similar to that of tonoplasts in plant cells [38]. We propose that the mechanism of HFB secretion by aerial hyphae starts from the intracellular accumulation of HFBs in the lipid-enriched organelles and hydrophobic/hydrophilic interface of VMSs or other vesicles. It is followed by the release of HFB-enriched vesicles through the plasma membrane to the periplasm, from where they are squeezed out through the cell wall and can disintegrate or partially remain intact (S16 Fig and S2 Video). The secretion of large VMSs may also cause hyphal bursts (Figs 3 and 4). The process is likely further stimulated by the increased intracellular turgor pressure that is also influenced by intracellular HFBs. This hypothesis is also supported by the observations of enlarged bursting cells of *P*. *pastoris* overexpressing HFBs (S7 Fig). This fungus has likely no mechanisms for targeting HFB vesicles to the periplasm; therefore, excess HFBs result in cell rupture due to increased intracellular pressure.

### Secretion of the massive extracellular HFB-enriched matrix precedes the formation of spore rings

**Fig 5:**
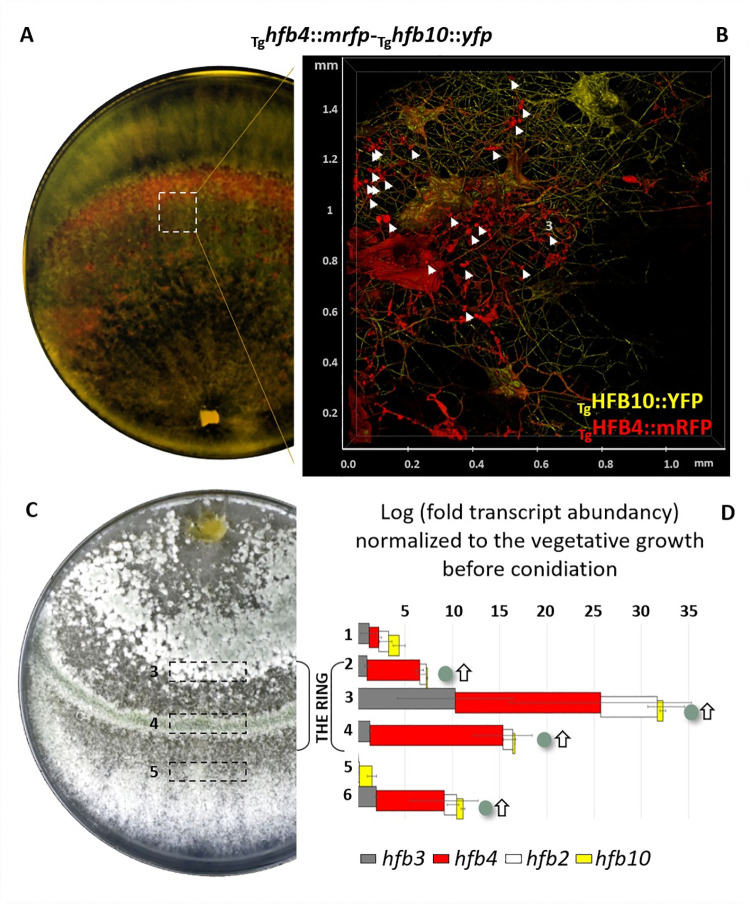
Colony architecture and dynamic HFB accumulation in aerial hyphae before and during conidiogenesis. (A) Fluorescent imaging of early conidiating *T*. *guizhouense* colony (48 hours) with mRFP- and YFP-labeled HFB4 and HFB10, respectively. The time course images of Tg and Th observed in ChemiDoc are shown in S17 Fig. (B) A 3D reconstruction of the same colony under epifluorescent microscope. Arrows point to the conidiophores with spore clumps. S3 Video shows detailed 3D animations of the extracellular matrices for both species. (C) A colony of *T*. *guizhouense* forming the sporulating ring after 96 hours of incubation. (D) Relative expression of *hfb*s during the fine time course of conidiogenesis (stages 1 to 6 correspond to the hyphal growth without conidia as 1, early compact pustula formation as 2, appearance of the conidial ring as 3 and 4, the vegetative growth beyond the conidial ring as 5, and dispersed conidiogenesis as 6, also see S18 Fig the boxed areas in **C**) of Tg quantified by qPCR. The values are normalized to those of the housekeeping gene *tef1* and calculated in relation to aerial hyphae prior to conidiogenesis (stage 1). Horizontal bars indicate standard deviations. Green dots and white arrows right to the bars indicate spores and conidiophores appearing stages, respectively. Complementary results for Th are provided in S18 Fig.

The massive accumulation of HFBs in *Trichoderma* colonies turning to the conidiation is visible without magnification if fluorescently labeled mutants are placed in a fluorescent imaging system, such as ChemiDoc MP (Bio-Rad, USA) (Fig 5A). The time course observation showed the coordinated appearance of HFB4 and HFB10 prior to conidiation along with the formation of conidiophores (S17 Fig). The complementary monitoring of *hfb* gene expression during conidiogenesis demonstrated that in both species at least four *hfb* genes (*hfb2*, *hfb3*, *hfb4*, and *hfb10*) were significantly (up to several thousand-fold in Th, S18 Fig) upregulated precisely at the beginning of conidiophore formation compared to the vegetative hyphal growth (Fig 5D). In both species, the expression of *hfb3* was clearly apparent at this stage, while it was not considered significant when the entire aerial mycelium was tested (Table 1).

Observation of the intact colony surface (no added water) under epifluorescent microscope revealed an HFB-enriched protein matrix surrounding the spores and conidiophores (Fig 5A). A 3D reconstruction of the colony surface showed that the matrix has an uneven distribution of HFB4 and HFB10 (see the S3 Video), with HFB4 being more associated with the spores and HFB10 with the hyphae. Thus, the massive secretion of HFBs by aerial hyphae is likely a prerequisite for conidiation not only because they are essential spore protectants [1, 10] but also because intracellular HFBs aid the formation of all hydrophobic structures of fungal colonies, such as aerial hyphae, conidiophores, and spores.

### Small water droplets aid the coating of spores by HFBs

**Fig 6:**
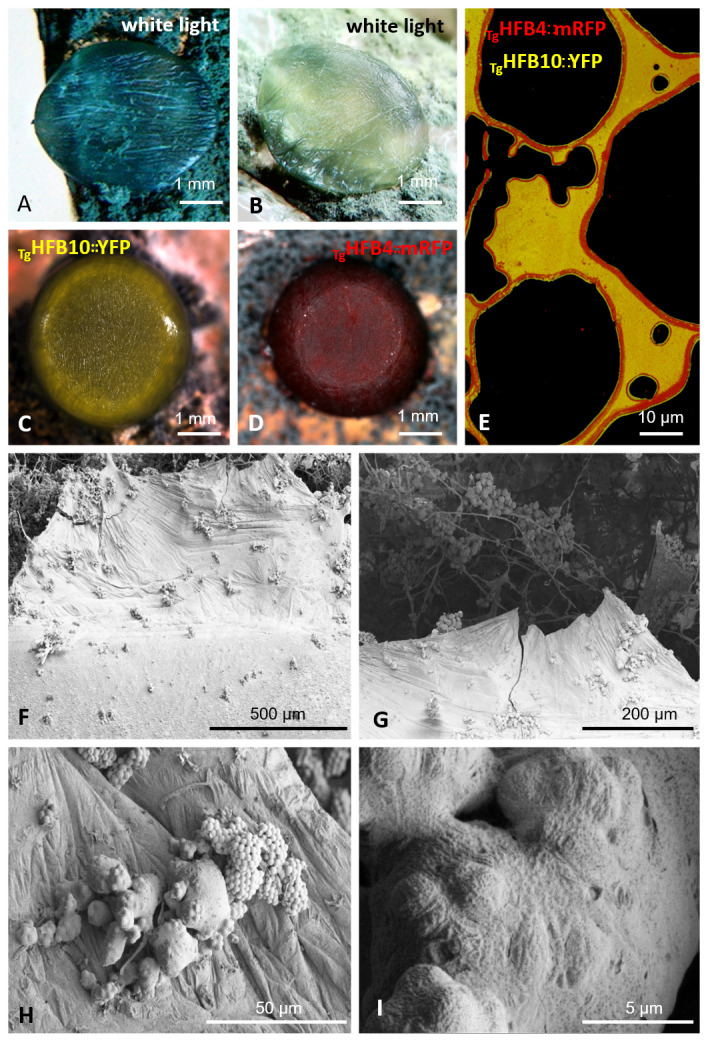
HFB-containing macroscopically visible films on the surface of water drops applied on the sporulating colonies of *Trichoderma* spp. (A) and (B) Droplets of 10 μl of water applied on the conidiating areas of *T*. *guizhouense* and *T*. *harzianum* colonies imaged 10 min after exposure to the open air at room temperature. (C) and (D) The stereo confocal microscopy images of 10 μl of water droplets applied on the conidiating areas of _Tg_*hfb10*::*yfp* and _Tg_*hfb4*::*mrfp* strains, respectively. (E) The fragment of the film formed by _Tg_*hfb4*::*mrfp—*_Tg_*hfb10*::*yfp* after it was destroyed by the cover glass and imaged in epifluorescent microscope. (F)–(G) and (H)—(I) are the films shown in (A) and (B), respectively, observed in cryo-SEM.

**Fig 7:**
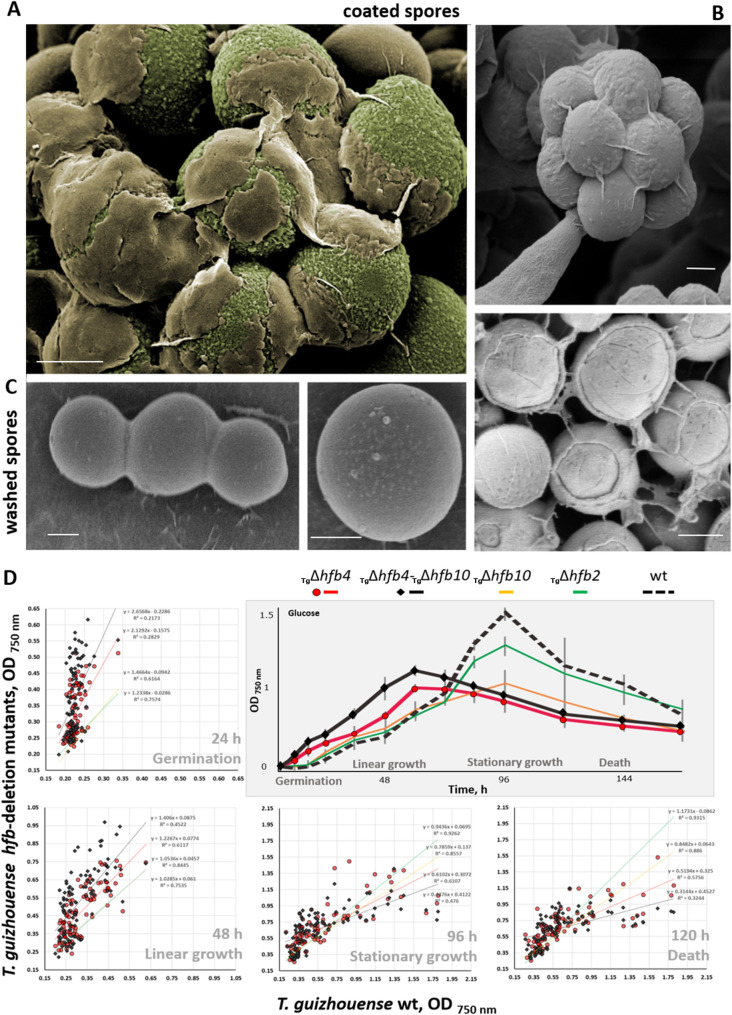
The water-soluble HFB-enriched coat of *Trichoderma* spores and its role in spore germination. (A) Premature Tg conidia with an uneven coat of extracellular proteins. Note: image was artificially colored for clarity. (B) A mature spore clump and spores evenly coated by a HFB-containing protein matrix. (C) Water-washed conidia on the surface of a glass slide. Scale bar = 1μm. (D) Scatter plots showing the growth of HFB-deficient mutants of *T*. *guizhouense* (Y-axis) against that of the parental strain (X-axis), as measured by the OD_750_ in FF Biolog microplates (see the Materials and Methods for the details). Every value corresponds to an individual carbon source (N = 96 incl. water). Each value for the mutant was calculated from at least three biological replicates for at least two mutants. Black diamond and red circle markers represent the _Tg_Δ*hfb4* and _Tg_Δ*hfb4*-_Tg_Δ*hfb10* mutants, respectively. The trend lines demonstrate the growth of these and also the _Tg_Δ*hfb10* and _Tg_Δ*hfb2* mutants. The results for individual carbon sources for *T*. *harzianum* and the respiratory activity for both species are given in S1 Dataset. An insert shows a representative example of the growth curves of *T*. *guizhouense* and respective *hfb*-deletion mutants on glucose as obtained from the FF Biolog microplates.

Considering the superior affinity of HFBs to self-assemble at interfaces, we applied water droplets on the surface of a conidiating colony (what also imitates pluviophilous conditions of rain or dew). Every drop became quickly (1 to 3 min) coated by a macroscopic film consisting of multiple layers of HFBs (Fig 6 and S4 Video). Notably, strains lacking HFB4 in Tg could not form such films (S4 Video). Microscopic observations of such drops allowed us to monitor the spore-coating mechanism during drying of water (even though a substantial portion of HFBs was lost as they assembled on the glass surface). The animation in S5 Video showed that if a drop of water was applied on the colony surface, HFB4 and HFB10 on the hyphae and spores would be rapidly dissolved in the water and assembled in a complex multilayer at the water/air interface. The drying of such droplets resulted in a shrinking and coating on spores in a HFB “pack” (Fig 7B). The reduced fitness of spores from Tg strains lacking *hfb4* or *hfb10* [1] indicates the importance of this coat for spore survival.

Thus, we propose that an even coating of spores by water-soluble HFBs can be achieved in the presence of water droplets that may be either guttated by the fungus [39–42] or derived from the environment.

### HFB4 acts as a water sensing regulator and dormancy/germination controller in *T*. *guizhouense* spores

We noticed that spores of the Tg strains lacking HFB4 frequently germinated *in situ* above the parental colony before dispersal (S19 Fig). Therefore, we hypothesized that the *hfb4*-deleted and/or (*hfb4* and *hfb10*) double-deleted strains became impaired in either the control of spore dormancy or initiation of germination. Because this developmental step may be influenced by the carbon source, we averaged this factor by monitoring the growth of the *hfb*-deletion mutants in Biolog FF Microplates on 95 carbon sources and water. Indeed, the Tg strains lacking HFB4 did not show a lag phase and germinated almost immediately after inoculation (Fig 7D). The lack of HFB10 did not significantly influence the germination rate, although the lack of both HFBs resulted in the fastest germination. Similar to the observations from the potato dextrose agar (PDA) cultivations (see Fig 4A), in these experiments, the strains lacking HFB4 had a significantly shorter life span than the wild type strain (Fig 7D). We observed that HFB4- and/or HFB10-deletion mutants (_Tg_Δ*hfb4* and _Tg_Δ*hfb4*-_Tg_Δ*hfb10*) were not able to reach the same final biomass as the wild type strain before the biomass started to decline (Fig 7D). This finding also pointed to the role of intracellular HFB4 in cellular metabolism and aging (*see above*).

**Fig 8:**
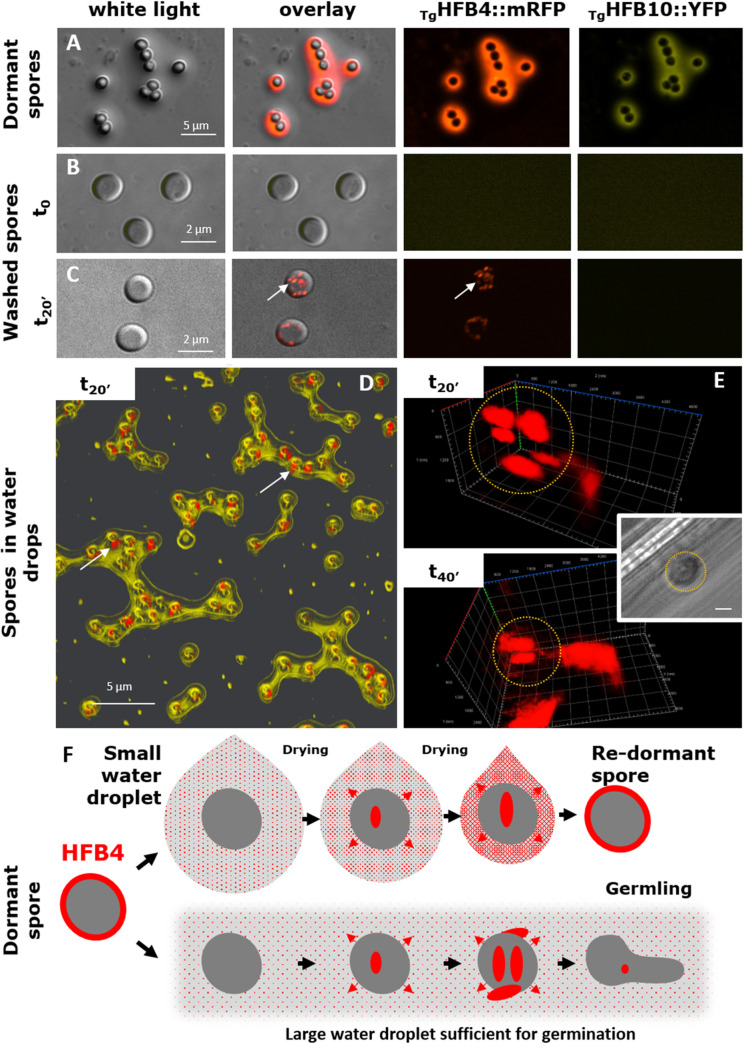
Dynamics of HFB4 secretion in spores of *T*. *guizhouense*_*Tg*_*hfb4*::*mrfp-*_*Tg*_*hfb10*::*yfp* after water washing. (A) Dormant spores on a glass cover slide. A coat containing HFB4::mRFP and HFB10::YFP is visible in a confocal microscope. (B) Washed spores imaged under a confocal microscope. (C) Same as in (B) but after incubation for 20 min (t_20’_) in excess water. The synthesis of HFB4:mRFP became visible (arrows). (D) A 3D reconstruction of the recovery of HFB4 and HFB10 coating on the spores drying from small water droplets. Arrows point to intracellular HFB4::mRFP; extracellular HFB10:YFP (yellow) assembles in the water/air and interface. (E) A 3D reconstruction of the super-resolution CLSM images showing the secretion of HFB4::mRFP by a washed spore incubated in water for further 20 minutes after imaging in (C) and (D). (t_20’-_t_40’_). The inset shows the white light image of this spore. Scale bar = 1 μm. (F) Flowchart showing a proposed role of HFB4 in the water sensing and dormancy maintenance in spores of *T*. *guizhouense*. When a HFB4-coated spore is placed in a small water droplet, the coat is washed away and HFB4 is dissolved. This event is followed by the immediate activation of the translation and secretion of HFB4 in spores. While the water droplet shrinks because of drying, the extracellular concentration of HFB4 increases, which putatively signals to the spore that environmental moisture is not sufficient for germination. Thus, the spore remains dormant and drying results in the recovery of the HFB4 coat. If a spore lands in a large water droplet, the HFB4 on the spore surface will be washed away, thus resulting in a low concentration of this protein in the solution. Due to the relatively large drop size, the subsequent secretion of HFB4 also does not result in a significant increase in HFB4 concentration. This decreased concentration of HFB4 putatively signals that the moisture in the environment is sufficient for germination. Note: deletion of the *hfb4* gene leads to nearly immediate germination of spores (Fig 7D).

Washing off the water-soluble HFB4 and HFB10 coat from the dormant spores activated the high production of HFB4 (Fig 8A–8E) in Tg after 20–30 min of incubation in water. Interestingly, the appearance of fluorescently labelled HFB4 (namely HFB4::mRFP) inside spores could be directly observed under the epifluorescent microscope or the super resolution CLSM. In a small water droplet, most of washed out HFB10 assembled at the solid-water and air-water interfaces (Fig 8D) while HFB4 remained mainly in the solution. With the shrinking of a small water droplet, the concentration of HFB4 in the spore-surrounding environment increased (Fig 8D). We propose that this process signaled to the spore that moisture was insufficient for germination and dormancy had to be maintained. After the drop dried, the coat containing at least these two HFBs was recovered and the spore remained dormant (Fig 8F). In a large water drop that did not dry for several hours, the secretion of additional HFB4 by the spore did not result in a substantial increase in HFB4 concentration or recovery of the HFB coat on the spores (Fig 8F). We propose that the prolonged “naked” stage when the spore was not covered by HFBs signaled that the environment was sufficiently moist for germination.

The sensing regulator role of HFB4 would be possible only if this protein could migrate through the coat of HFB10 on the spore surface (Fig 8A, 8D, 8E and 8F). Consistent with this hypothesis, a quartz crystal microbalance measurement with dissipation monitoring revealed no interaction between the recombinant _Tg_HFB10 and the _Tg_HFB4 (S20 Fig). Nonmixing layers of fluorescently tagged HFB4 and HFB10 are also shown in Figs 1, 5 and 7. Based on the above results, we propose a water sensing role for HFB4 and its involvement in spore dormancy control in Tg.

Interestingly, we also did not observe cases of HFB co-localization with lipids in spores (S9 Fig). It may suggest that in spores, HFBs follow the conventional secretory pathway avoiding the intracellular accumulation step.

## Discussion

Since their discovery, HFBs have been known to play multiple extracellular roles in filamentous fungi [14, 43]. Located outside of the cell, HFBs assemble at hydrophobic/hydrophilic interfaces, modulate surface properties, and mediate a broad spectrum of fungal interactions with their environment [8]. This study provides the insight in pleiotropic functions of HFBs inside fungal cells, which essentially extends our understanding of the functionality of these proteins and further explains their universal presence in fungi.

### The putative role of HFBs in conidiophore formation through the regulation of turgor pressure

In many molds, including *Trichoderma* spp., conidiophores are not evenly distributed on the colony surface but appear in pustules or form characteristic conidial rings in response to circadian rhythms [44, 45] or other stimuli [46]. We showed that in *Trichoderma* colonies, the emergence of such conidiation hot spots starts with the intracellular accumulation of HFBs. We also observed high upregulation of multiple HFB-encoding genes either before or at early stages of conidiogenesis. In turn, *Trichoderma* mutants deprived of major HFBs produce significantly fewer spores than the parental strain; however, they still tend to form conidial rings or pustules. We assume that these proteins are required for normal (= abundant) conidiation and the formation of fit spores [1], but the process itself is triggered by other factors, such as starvation, illumination or other stresses [44].

Prior to their extracellular release, HFBs accumulate inside sporogenic hyphae. We propose that this accumulation has a dual function: first, it is associated with the secretion for the subsequent spore coating, but it also helps the erection of conidiophores. Both of these functions are potentially linked to intracellular turgor pressure. The aerial hyphae of the *hfb*-deletion and *hfb*-overexpressing strains had signs of reduced and increased turgor, respectively. The apical hyphal tips of *hfb4*-deletion strains of Tg had characteristic indentations, although in healthy *Neurospora crassa* hyphae, these areas were known to have the highest turgor pressure [34]. This fact alone suggests the role of HFBs in turgor pressure. Furthermore, HFB-overexpressing strains of both species and conidiophores of the wild-type strains had a visibly inflated appearance and were frequently covered by multiple large herniations filled with HFB-containing vesicles. The putative role of intracellular HFBs in turgor pressure is also confirmed by the outstanding resistance of the HFB-overexpressing mutant to the osmotic stress. The later fact opens an avenue for the follow up investigation of the potential involvement of HFBs or other surface-active proteins in fungal resilience to the osmotic stress.

Considering that the cell wall ultrastructure was not visibly changed, these observations suggested increased turgor pressure when HFB production was either naturally (conidiophore formation) or artificially (by using a strong constitutive promoter controlling *hfb* genes) stimulated. We also speculate that the lack of conidia in HFB-deletion mutants was caused by an alteration of intracellular turgor pressure required for conidiophore emergence.

To date, the understanding of turgor pressure in fungi is limited to studies of hydrophilic trophic hyphae frequently observed in fungi-like protozoans (Oomycota) [47], plant-pathogenic fungi [48, 49] or *N*. *crassa* [50] and explained by cytoplasm and water flow, osmosis and regulation of the mitogen-activated protein kinase cascade [34, 36]. However, these studies are not relevant to aerial hyphae where HFB-encoding genes are highly active. The mechanisms of turgor pressure modulation in aerial hyphae are far not understood [51]. In aerial hyphae, turgor pressure is required not only for apical elongation but also for growth against the force of gravity and production of mechanically stable conidiophores. However, because aerial hyphae are usually hydrophobic (coated by HFBs or other surface-active proteins), their ability to absorb water and exchange ions within the environment is limited [51]. Thus, the available methods to measure turgor are not applicable. Because HFBs showed affinity to conidiophores and phialides, we propose that they contribute to the specific mechanisms for turgor pressure regulation in aerial structures. Detailed observations of conidiophore morphology in more than 200 *Trichoderma* spp. taxonomically characterized by W. M. Jaklitsch [52, 53] (s*ee also* S13 Fig) showed that herniations of conidiophores are very common, at least in these fungi. The accumulation of HFBs in tonoplasts, where they form or line up the membranes (*see below*), may increase their stability and could represent a major factor underlying turgor pressure formation in these specific cells. In this sense, the biological function of HFBs in *Trichoderma* observed in this study resembles the role of HFBs in the formation of mushrooms, where they provide hydrophobicity to fruiting bodies and contribute to the mechanical stability of aerial channels in basidiocarps [35, 54]. Interestingly, similar conclusions were proposed when the first hydrophobins in Ascomycota were discovered [14, 55]. But none of those studies attributed this functionality to intracellular accumulation. Here, we can generalize that intracellular HFBs are essential for the formation of sporogenic structures and therefore are universally present in all filamentous fungi regardless of the hydrophobicity of the hyphae and spores [11, 56]. Consequently, the accumulation and secretion of HFBs in the aerial mycelium largely explain the development and architecture of mold colonies.

### HBF secretory pathway in aerial hyphae

Fungi must be able to secrete abundant HFBs to coat spores, thereby protecting against abiotic stresses [1, 26, 57] and supporting attachment to hosts or substrates [58]. However, the details of protein secretion by aerial hyphae have received essentially less attention. Here, we uncovered a distinctive secretory pathway of HFBs in aerial (nonfeeding and hydrophobic) hyphae that includes their accumulation in lipid-enriched organelles (putative lipid bodies), the formation of putative HFB-enriched vacuolar vesicles [33] for an intracellular accumulation step, transport to the periplasm, and release to the outer space by squeezing through the cell wall. Our results show that this pathway is not universally active in all parts of the fungal colony but is tightly determined by the involvement of HFBs in conidiation [26, 58–60]. Fluorescence imaging revealed the abundant and coordinated intracellular accumulation of HFBs by aerial hyphae shortly before the start of conidiophore formation. Then, the sporulation onset was accompanied by the massive extracellular release of HFBs from nontrophic aerial hyphae and conidiophores. This process may explain how the colony efficiently prepares for coating newly formed spores in protective HFB-enriched envelopes [1].

### Intracellular compartmentalization of HFBs

Massive accumulation of highly surface-active molecules in an eukaryotic cell that has multiple compartments and membranes (i.e., potential interfaces) presents risks for normal metabolic processes. Our results show that both filamentous fungi and yeasts can tolerate high amount of intracellular HFBs. Initially, we hypothesized that in both fungi, HFB-containing organelles filled some of the cell compartments and influenced intracellular homeostasis. However, an expression analysis of ER stress marker genes, autophagy, and the unconventional protein sorting pathway revealed that neither *P*. *pastoris* nor *Trichoderma* cells were stressed, which was also confirmed by the visually healthy colonies.

Due to the presence of the characteristic signal prepropeptide sequence, HFBs are initially translated into the ER lumen [25], where the cysteine residues become oxidized and disulfide bonds are formed [61]. Our results suggest that in spores, trophic and hydrophilic hyphae, where only a limited amount of HFBs may be required and *hfb* genes are generally moderately active or downregulated, HFBs become conventionally secreted into the medium. The accumulation of HFBs in aerial hyphae (where *hfb* genes are highly active) probably started when these proteins become translocated from the ER to lipid-enriched organelles. The precise identification of these organelles as canonical eukaryotic ER-derived lipid bodies [62] was beyond the scope of our investigation, However, the future studies may address question along with the investigation of HFBs affinity to lipids. The colocalization of fluorescently labeled HFBs and lipids was observed in aerial hyphae of *Trichoderma* as well as in *P*. *pastoris*. Previous studies have shown the ability of various HFBs to stabilize lipid emulsions [63, 64]. Our TEM analysis revealed that lipid droplets of HFB-overexpressing strains were more electron dense than those of the control strains, which may be caused by the enrichment of dissolved HFBs; however, this hypothesis requires further verification. Remarkably, Hähl et al. [33] demonstrated that HFB1 from *T*. *reesei* (which is chemically similar to HFBs studied here [19]) can form pure bilayer HFB vesicles stable in a variety of liquids and sizes. Our observation that aerial hyphae and conidiophores in particular possessed large vacuoles or tonoplast-like structures that were massively HFB-positive indicated that intracellular HFBs formed similar membranous vesicles (HFB vesicles), as described in a cell-free system [33].

Comparisons of micrographs from super-resolution fluorescent *in vivo* microscopy and TEM indicate that HFBs did not fill in these organelles but instead lined up their membranes. TEM micrographs of the wild-type, *hfb* deletion and HFB-overexpressing hyphae revealed occasional events of lipid-enriched organelles-to-vacuole internalization at an equal frequency in all strains, and such events were also reported for other fungi [32]. HFB-overexpressing strains were enriched in specific organelles that we assigned as VMSs containing HFBs. Such vesicles were usually spherical and frequently included in one another, thus forming one large tonoplast resembling a “matryoshka”. We speculate that such structures were formed after internalization of HFB-enriched lipid droplets in vacuoles. In this case, HFBs likely assembled at the lipid/water interface and formed HFB membranes in VMSs. Importantly, the formation of VMSs with putative HFB vesicles was also observed in recombinant *P*. *pastoris* cells producing HFBs.

### Extracellular vesicles containing HFBs

In *Trichoderma*, the release of putative HFB-enriched vesicles occurred first in the periplasm through the fusion of VMSs with the plasma membrane or budding. Consistently, the periplasm volume of *hfb*-deletion mutants was substantially reduced compared to that of the wild-type strains and HFB-overexpressing mutants. Our results do not allow us to conclude whether HFB membranes dissociate after/during release from the cells; however, we observed extracellular HFB-enriched vesicles. Previous reports stated that HFBs are secreted in a soluble form [7]. The solubility of HFB layers was used for grouping HFBs in conventional classes, where class I HFBs formed insoluble functional amyloids (rodlets), while the layers of class II HFBs (which were studied here) were soluble in organic solvents and ethanol [7, 20, 21]. However, Winandy et al. [63] showed a similar stability of layers formed on glass by HFBs from both classes, and Hähl et al. [65] demonstrated the stability of HFB vesicles formed by HFB1 (class II). Therefore, we speculate that HFBs can contribute to the formation of extracellular vesicles that attract attention in cell biology as intracellular delivery and communication vehicles [66], as demonstrated in fungi [67], including *Trichoderma* [68].

Subcellular vacuolar multicisternal structures similar to VMSs and fingerprint bodies detected in this study have been observed in many fungi, such as in studies characterizing the ultrastructure of *T*. *reesei* during cellulase production [31] or investigating the benzyl-penicillin-overproducing strains of *Penicillium* sp. [69]. VMSs formation and the presence of membrane-covered lipid droplets were associated with a drastic increase in cell wall thickness during penicillin biosynthesis. Structures resembling VMSs were also detected in the nematophagous fungus *Arthrobotrys oligospora* [70] and in arbuscular mycorrhizal fungi [71], and they are commonly seen in TEM micrographs [70] of fungi producing HFBs from both classes.

### The role of HFBs in spore dormancy and water sensing

In this study, we observed a water-aided mechanism of spore coating by extracellular HFBs. Mature *Trichoderma* colonies were covered by a massive HFB-enriched matrix that was loosely attached to hyphae and was therefore easily washed away by water. Drops of water either guttated or externally applied (resembling rain or dew) became covered by macroscopically visible multiple layers of HFBs that had a complex structure, and individual HFBs visibly did not mix or interfere. Using fluorescent proteins, we showed how spores became packed in HFBs in such droplets and speculated that microscopic water aids the even coating of spores by HFBs, which is essential for spore maturation and therefore fitness. Further ecological implications of this finding can provide an explanation of the conidiation dynamics in molds that are not always explained by illumination or other stress factors. Similar to Basidiomycota fungi, which require a particular range of air humidity for basidiospore discharge [72], molds may also form conidia in response to environmental conditions that provide sufficient moisture for the formation of mature HFB-coated spores suitable for dispersal.

Interestingly, *hfb*s are actively transcribed and translated into HFBs if putatively dormant spores are wetted. *Trichoderma* spores are coated by class II HFBs that are considered essentially more soluble than insoluble rodlet-forming HFBs from class I, known from *Aspergillus* spp. and other fungi [7, 73]. Using fluorescently labeled *Trichoderma* HFBs, we observed that the HFB coat of spores consisting of HFB4 and HFB10 was easily removed by water. Although both HFBs then assembled at the solid/water and water/air interfaces, the majority of HFB4 remained in the solution. Another set of unexpected findings in this study was linked to the role of *hfb* genes in spore dormancy control. A gene expression analysis of washed spores (as required by the method) indicated a constant relatively high level of *hfb10* expression but low level of *hfb4* expression. However, we observed the extensive translation of fluorescently labeled HFB4 if washed spores of Tg were incubated in water for 20 min or more. Moreover, we noticed that spores of HFB4-deletion mutants or mutants lacking HFB4 and HFB10 had no lag phase and germinated almost immediately. Putting these facts and the affinity of HFB4 to remain in the solution together, we propose a water sensory function for this protein. We assume that the germination of Tg preferentially occurs in water. This species forms fluffy buoyant colonies and shows properties of an aero-aquatic fungus [23, 74]. Thus, when a HFB4-coated spore is placed in a small water droplet, its germination is not favorable because the drop may dry and the germling will not survive. HFB4 becomes washed away and dissolved in the droplet. This exposure is followed by the activation of the translation and immediate secretion of HFB4 in spores. While the small water drop shrinks because of drying, the extracellular concentration of HFB4 increases, which can signal to the spore that environmental moisture is not sufficient for germination. Thus, the spore remains (re)dormant, and drying results in the recovery of the HFB coat (HFB4 and HFB10). If a spore lands in a large water droplet, germination becomes risk-free. The HFB4 coat on the spore surface is washed away, resulting in a low concentration of this protein in the solution. Due to the relatively large drop size (water volume), the subsequent secretion of HFB4 by the spore also does not result in a significant increase in HFB4 concentration or in a HFB4 coat on the spore surface. This environment signals to the spore that the moisture in the environment is suitable for germination.

Thus, HFBs are essential accessory proteins with a range of pleiotropic functions in fungal development and thus, are essential for fungal fitness.

## Materials and methods

### Strains and cultivation conditions

All strains used in this study are listed in Table 2. If not otherwise specified, filamentous fungi were maintained on PDA (Sigma, USA) at 25°C in dark conditions. *Komagataella pastoris* (Saccharomycetales, syn. *Pichia pastoris*) was maintained on yeast extract peptone dextrose medium (YPD) at 28°C. Yeasts were cultivated in buffered minimal glycerol medium (BMG, including 100 mM potassium phosphate, pH 6.0, 1.34% yeast nitrogen base, 4 × 10^−5^% biotin and 1% glycerol) and then transferred to buffered minimal methanol medium (BMM, including 100 mM potassium phosphate, pH 6.0, 1.34% yeast nitrogen base, 4 × 10^−5^% biotin and 0.5% methanol) for the designed protein production. All strains are available from the TU Collection of Industrial Microorganisms (TUCIM) at TU Wien, Vienna (Austria) and Nanjing Agricultural University, Nanjing (China). For the testing the resistance to the osmotic stress, strains were inoculated in the glucose synthetic medium (9.89 mM KNO_3_, 7.35 mM KH_2_PO_4_, 6.7 mM KCl, 2.03 mM MgSO_4_ × 7H_2_O, 0.9 mM CaCl_2_, 0.094 mM MnSO_4_ × H_2_O, 0.048 mM ZnSO_4_ × 7H_2_O, 0.18 mM FeSO_4_ × 7H_2_O, 0.121 mM CoCl_2_ × 6H_2_O, 1% glucose) supplemented with NaCl (0.75 M, 1 M, or 1.25 M), sucrose (1 M or 2 M), or 2 M glycerol as osmolytes. Experiments were performed in 96-well microplates inoculated with 10^6^ spores/ml and incubated at 25°C in darkness. Each assay was repeated four times.

### Molecular biology techniques

#### Gene expression

For the gene expression analysis, fungal biomass corresponding to the four stages of the life cycle, namely, (i) germlings, (ii) trophic hyphae, (iii) aerial hyphae, and (iv) conidiophores and conidia, were collected from cellophane-covered agar plates or liquid cultures. Total RNA was extracted using an RNeasy Plant MiniKit (Qiagen, Germany) according to the manufacturer’s protocol. cDNA was synthesized with a RevertAid First Strand cDNA Kit (Thermo Fischer Scientific, USA) using the oligo (dT)^18^ primer. qPCR was performed using qTOWER (Jena Analytics, Germany) for the genes of interest (listed in Table 1) and calculated by the 2^-ΔΔCt^ method [75] using *tef1* as the housekeeping gene [76]. PCR was prepared in a total volume of 20 μl containing 10 μl of iQ SYBR Green Supermix (Bio-Rad, USA), 0.5 μM each primer and 100 ng of cDNA. The program was 40 cycles with 30 s at 95°C and 60 s at 60°C, which was initially denatured for 6 min at 95°C. A melting curve from 55°C to 95°C was generated for each qPCR run.

#### Gene deletion and reverse complementation

The polyethylene glycol (PEG)-mediated protoplast transformation procedure was adopted [76]. Gene deletion in Tg and Th was performed as described previously in Cai et al. [1] using a hygromycin B cassette (*hph*, from the plasmid pPcdna1-hph [77]) and/or geneticin cassette (*neo*, from the plasmid pPki-Gen [78]) to replace the gene of interest (S1 File). For reverse complementation, a copy of the gene with its promoter and terminator region from the wild-type strain or a copy of this gene with a fluorescent tag (*mrfp* or *yfp*, see below) fused at the C-terminus was amplified and inserted into the corresponding deletion mutant. The positive mutants were further confirmed at the transcriptional level. All primers used in this study are given in S1 Table.

#### *In vivo* fluorescent labeling

HFB-encoding genes were conjugated with genes encoding fluorescent proteins (followed by a 6×His tag) at the C-terminus over a synthetic GGGGS×3 linker [79]. Briefly, a 1.2 kb fragment including the entire *hfb* gene before the stop codon (namely, the 5’ homologous arm), a 1.0 kb fragment of its native terminator region and a 1.2 kb fragment after the terminator region (namely, the 3’ homologous arm) were amplified by PCR and then cloned into the pUC19 plasmid (Thermo Fisher Scientific, USA) in the order of the 5’ homologous arm, the fluorescent protein gene (either yellow fluorescent protein (YFP; excitation at 514 nm and emission at 527 nm) or mutated red fluorescent protein (mRFP; excitation at 559 nm and emission at 630 nm), terminator region, selection marker cassette and 3’ homologous arm by a ClonExpress MultiS One Step Cloning Kit (Vazyme, China). The obtained plasmids were confirmed by Sanger sequencing to determine the in-frame fusion constructs and transformed into *Trichoderma* with the above two selection markers. For the putatively labeled strains, transformants from PEG-mediated protoplast transformation were screened by PCR using the same strategy as above and confirmed by the presence of the corresponding fluorescent signals by a Leica DMi8 microscope with fluorescent applications (Leica, Germany). Additionally, a mutant expressing mRFP under the control of an *hfb* promoter (P_*hfb4*_) was generated. All PCR products and vector constructs were verified by sequencing. Details on the mutant construction process are provided in S1 File.

#### Overexpression of *hfb* genes in *Trichoderma*

Overexpression vectors were constructed by cloning a 1.6 kb fragment that contained the open reading frame (ORF) of the *hfb* gene and its terminator region into the pUCP*cdna1*-*hph* plasmid after a constitutive promoter, P_*cdna1*_, from *T*. *reesei* QM 6a [72]. The PCR products were purified and fused into a ClaI-digested plasmid with an In-Fusion HD cloning kit (TAKARA, Japan). Mutants for overexpressing HFB2 (_Tg_OPB38530) fused with a mRFP tag were generated with the fluorescent construct mentioned above.

#### Heterologous expression of *hfb* genes from *Trichoderma* in *P*. *pastoris*

An EasySelect *Pichia* Expression Kit was used to express genes from *Trichoderma* in the *P*. *pastoris* strain KM71H (Invitrogen, USA) according to the manufacturer’s instructions. The encoding region after the end of the predicted signal peptide of the *hfb* gene (SignalP 4.1) to the stop codon was amplified from the cDNA of the wild-type strain. The cloned fragment was inserted into the plasmid pPICZαA containing the AOX1 promoter by replacing the fragment between the EcoR I site and Xba I site using an In-Fusion HD cloning kit (Clontech, Japan). To express recombinant proteins with a fluorescent tag, a fluorescent protein-encoding gene (a green fluorescent protein variant (i.e., GFPuv, excitation at 395 nm and emission at 509 nm) was inserted at the C-terminus of the designated *hfb* gene. Additionally, the recombinant protein encoded by this construct contained the *Saccharomyces cerevisiae α*-mating *factor* prepropeptide [80] at the N-terminus of the *hfb* gene and a His×6 epitope at the C-terminus. The resulting vector was linearized with Sac I or Pme I and then transformed into *P*. *pastoris* by electroporation. One of the immunoblotting-positive transformants (*see below*) was chosen to obtain the recombinant protein.

### Biochemical and biophysical techniques

#### Protein detection techniques

For sodium dodecyl sulfate-polyacrylamide gel electrophoresis (SDS-PAGE) and immunoblotting assays, protein samples from the surface of *Trichoderma* spores or hyphae were collected by washing with 1% SDS (pH 8.1, 50 mM HCl-Tris). The *Pichia*-produced proteins were collected from BMM fermentation (see *P*. *pastoris* fermentation in Przylucka et al. [26]). Protein samples were then denatured and loaded into a 15% polyacrylamide gel, followed by silver staining with a SilverQuest Silver Staining Kit (Life Technologies, Germany) suitable for HFBs [26]. Immunological visualization of HFBs was performed using a mouse anti-His-tagged horseradish peroxidase (HRP) antibody (Genescript, USA). The target proteins were visualized following the protocol supplied with Clarity Western ECL (Bio-Rad, USA) or with the ONE-HOUR Western Standard Kit (Genscript, China).

#### Protein-protein interaction by quartz crystal microbalance (QCM) measurement with dissipation monitoring

Quartz crystal sensors (5 MHz, AT-cut, gold electrode, LOT Quantum Design, Germany) coated with a homogeneous borosilicate film were used for monitoring the representative adsorption kinetics of *P*. *pastoris*-produced HFBs. QCM measurements were conducted at 25°C with a Q-Sense E4 QCM-D system (Biolin Scientific, Sweden) for three biological replicates. The measurement cell was injected with a solution containing 5 μM purified _Tg_HFB4 (_Tg_OPB37525) until the surface was saturated, followed by a 5 μM _Tg_HFB10 (_Tg_OPB44696) application. The Q-Tools 3.1 software package (Biolin Scientific, Sweden) was used for data extraction and normalization.

#### Surface hydrophobicity

For the surface hydrophobicity assay, a drop of 10 μl of distilled water was placed on the surface of a fungal colony through an OCA 20 (DataPhysics, Filderstadt, Germany). Videos of droplet formation on the colony surface were recorded through a goniometric eye piece (Krüss GmbH, Germany) with a horizontal light path. The wettability of the fungus was then expressed by the water contact angle (θ).

### Phenotype investigation

#### Macromorphology

The strains were cultivated on PDA plates for seven days at 25°C in darkness. The colony morphology was recorded by a Cannon EOS 70D (equipped with a Cannon 100 μm macro lens) under white light. The development of the colonies of the fluorescently labeled *Trichoderma* strains was monitored by making regular (every 24 h) images in a Bio-Rad ChemiDoc MP system (Bio-Rad, USA) equipped with multiplex fluorescent channels. The system was first optimized based on the florescence of single-labeled strains and then applied for the double-labeled mutants.

#### Biolog phenotype microarrays

The growth rates and carbon utilization profiles of the strains were monitored using a phenotype microarray system with Biolog FF microplates for filamentous fungi (BIOLOG, Hayward, USA) as described previously [81], with the following modifications. Briefly, *Trichoderma* spores were collected and suspended in sterile Milli-Q water in disposable borosilicate tubes. Then, 90 μl of the adjusted spore suspension (with a transmission of 75% ± 2% at 590 nm, ca. 10^7^ cells ml^-1^) was dispensed into each well. Microplates were sealed in the original bags and incubated at 25°C in darkness, and the OD_750_ and OD_490_ were measured at 12, 18, 24, 36, 48, 60, 72, 96, 120, 144 and 168 h. OD_750_ values were adopted for the biomass measurements [81], while respiratory activity (activity of the succinate dehydrogenase proportional to the formation of red color of the formazan dye integrated into the FF Biolog microplate) was calculated by subtracting the OD_750_ from the OD_490_ [82].

#### *In vivo* microscopy and fluorescent staining

The fluorescently labeled strains were imaged by a stereo confocal microscope (Olympus, MVX10, Japan). Protein localization in fungal cells was carried out by an UltraVIEW VoX Spinning Disk Confocal Microscope (PerkinElmer, USA) and a Leica DMi8 (Leica, Germany), and 3D images and videos were acquired by a Leica LAS X (Germany). The detailed intracellular localization of HFBs was performed by a fast superresolution laser confocal microscope (Zeiss LSM980 Airyscan2, Germany). For intracellular lipid staining, fungal cells were incubated in 2 μM BODIPY FL C_12_ (excitation 500 nm, emission 510 nm, Thermo Fischer Scientific, USA) for 15 min and washed with 50 mM PBS three times. Stained samples were imaged using a Leica DMi8 microscope (Leica, Germany) or LSM980 Airyscan2 (Zeiss, Germany).

#### Electron microscopy

Fungal colonies were investigated by cryo-SEM (Quorum PP3010T integrated onto a Hitachi SU8010 FE-SEM, Japan). The culture samples were rapidly frozen in nitrogen slush, fractured at -140°C and coated with 5 nm platinum.

The cell ultrastructure was investigated by TEM (Hitachi H-7650, Japan). Fungal cells were fixed in a 2.5% glutaraldehyde solution at 4°C overnight. Fixed cells were then washed with 0.1 M PBS three times, postfixed with 1% osmium tetroxide (for 2 h), washed with 0.1 M PBS again and dehydrated with a gradation of ethanol, namely, 50%, 70%, 90% and 100%, followed by 100% acetone. Dehydrated samples were infiltrated in graded acetone/epoxy resin and cured at 60°C for 48 h. Cured resin blocks were trimmed, sectioned at a 70-nm thickness and poststained with uranyl acetate and lead citrate (3%).

### Statistical analyses

The data were calculated and statistically examined by one-way analysis of variance (ANOVA) or multivariate analysis of variance (MANOVA) using STATISTICA 6 (StatSoft, Germany). Heatmap analysis and average linkage hierarchical clustering and PCA plots were obtained using R (version 3.2.2). The significance level was set at *p* < 0.05 unless otherwise stated.

## Supporting information

S1 Fig Principal component analysis (PCA) of the *hfb* expression pattern during *Trichoderma* development. (PDF)

S2 Fig Molecular dynamic simulation and solvent accessible surface area calculation of fluorescently tagged HFBs used in this study. (PDF)

S3 Fig Phenotypic characterization of HFB mutants. (PDF)

S4 Fig Intracellular accumulation of mRFP-labeled HFB4 in conidiophores. (PDF)

S5 Fig Localization of _Tg_HFB3::mRFP in vacuole-resembling organelles and on the surface of phialides near the collarette. (PDF)

S6 Fig Secretion of mRFP expressed using the signal peptide under the control of the promoter of _Tg_*hfb4*. (PDF)

S7 Fig Morphology and ultrastructure of *Komagataella pastoris* (Saccharomycetales, syn. *Pichia pastoris*) expressing *Trichoderma* HFB-encoding genes. (PDF)

S8 Fig Representative morphology of *Trichoderma* mutants lacking *rab7*. (PDF)

S9 Fig Fluorescent microscopy imaging and ultrastructure of *Trichoderma* spores. (PDF)

S10 Fig Morphology of _Tg_OE*hfb2*::*mrfp* and immunochemical characterization of HFB2 secreted by aerial hyphae. (PDF)

S11 Fig Full size TEM micrographs used for the photoplate in Fig 3. (PDF)

S12 Fig Quantification of autophagy-related genes in *Trichoderma* spp. and *P*. *pastoris* expressing *hfbs*. (PDF)

S13 Fig Herniations on conidiophores of *Trichoderma* spp. from the taxonomic work of W. M. Jaklitsch. (PDF)

S14 Fig Cell wall ultrastructure of HFB mutants of *Trichoderma*. (PDF)

S15 Fig Response of the wild type, HFB-deficient, and HFB-overexpressing strains of *T*. *guizhouense* to the conditions of the osmotic stress. (PDF)

S16 Fig Intracellular, periplasmic, and extracellular vesicles in mature aerial hyphae overproducing HFBs. (PDF)

S17 Fig Dynamic release of HFBs during conidiogenesis of *Trichoderma* and an architecture of colonies. (PDF)

S18 Fig Colony architecture and dynamic HFB accumulation in aerial hyphae before and during conidiogenesis. (PDF)

S19 Fig Secondary growth of _Tg_Δ*hfb4*. (PDF)

S20 Fig Interaction monitoring between HFBs using quartz crystal microbalance (QCM). (PDF)

S1 Video Intracellular localization of HFB2::mRFP in two-day-old aerial hyphae of the _Tg_OE*hfb2*::*mrfp* strain. (MP4)

S2 Video Intracellular, periplasmic, and extracellular vesicles in mature aerial hyphae overproducing HFBs. (MP4)

S3 Video Animated 3D reconstructions of extracellular HFB-enriched matrices coating sporulating *Trichoderma* colonies. (MP4)

S4 Video Showing the formation of the HFB-containing film on the surface of the water droplet applied on the conidiating colony of Tg and _Tg_Δ*hfb4*. (MP4)

S5 Video Animated image of HFB4 and HFB10 coating spores in the presence of water. (GIF)

S1 Dataset Results of the BIOLOG Phenotype MicroArrays. (XLSX)

S1 File Detailed Methods. (PDF)

S1 Table Primers used in this study. (XLSX)

## References

[1] Cai F, Gao R, Zhao Z, Ding M, Jiang S, Yagtu C, et al. Evolutionary compromises in fungal fitness: hydrophobins can hinder the adverse dispersal of conidiospores and challenge their survival. ISME J. 2020. Epub 2020/07/08. doi: 10.1038/s41396-020-0709-0 . 32632264 PMC7490268

[2] Elliot MA, Talbot NJ. Building filaments in the air: aerial morphogenesis in bacteria and fungi. Curr Opin Microbiol. 2004;7(6):594–601. Epub 2004/11/24. doi: 10.1016/j.mib.2004.10.013 . 15556031

[3] Holder DJ, Keyhani NO. Adhesion of the entomopathogenic fungus *Beauveria* (*Cordyceps*) *bassiana* to substrata. Appl Environ Microbiol. 2005;71(9):5260–6. Epub 2005/09/10. doi: 10.1128/AEM.71.9.5260-5266.2005 ; PubMed Central PMCID: PMC1214598. 16151112 PMC1214598

[4] Vila T, Nazir R, Rozental S, Dos Santos GM, Calixto RO, Barreto-Bergter E, et al. The Role of Hydrophobicity and Surface Receptors at Hyphae of *Lyophyllum* sp. Strain Karsten in the Interaction with *Burkholderia terrae* BS001—Implications for Interactions in Soil. Front Microbiol. 2016;7:1689. Epub 2016/11/12. doi: 10.3389/fmicb.2016.01689 ; PubMed Central PMCID: PMC5081359. 27833591 PMC5081359

[5] Wosten HA, van Wetter MA, Lugones LG, van der Mei HC, Busscher HJ, Wessels JG. How a fungus escapes the water to grow into the air. Curr Biol. 1999;9(2):85–8. Epub 1999/02/18. doi: 10.1016/s0960-9822(99)80019-0 . 10021365

[6] Taylor JW, Berbee ML. Dating divergences in the Fungal Tree of Life: review and new analyses. Mycologia. 2006;98(6):838–49. Epub 2007/05/10. doi: 10.3852/mycologia.98.6.838 . 17486961

[7] Wosten HA. Hydrophobins: multipurpose proteins. Annu Rev Microbiol. 2001;55:625–46. Epub 2001/09/07. doi: 10.1146/annurev.micro.55.1.625 . 11544369

[8] Sunde M, Pham CLL, Kwan AH. Molecular Characteristics and Biological Functions of Surface-Active and Surfactant Proteins. Annu Rev Biochem. 2017;86:585–608. Epub 2017/01/27. doi: 10.1146/annurev-biochem-061516-044847 . 28125290

[9] Wosten HA, Scholtmeijer K. Applications of hydrophobins: current state and perspectives. Appl Microbiol Biotechnol. 2015;99(4):1587–97. Epub 2015/01/08. doi: 10.1007/s00253-014-6319-x . 25564034

[10] Gebbink MF, Claessen D, Bouma B, Dijkhuizen L, Wosten HA. Amyloids—a functional coat for microorganisms. Nat Rev Microbiol. 2005;3(4):333–41. Epub 2005/04/05. doi: 10.1038/nrmicro1127 . 15806095

[11] Dubey MK, Jensen DF, Karlsson M. Hydrophobins are required for conidial hydrophobicity and plant root colonization in the fungal biocontrol agent *Clonostachys rosea*. BMC Microbiol. 2014;14:18. Epub 2014/02/04. doi: 10.1186/1471-2180-14-18 ; PubMed Central PMCID: PMC3922079. 24483277 PMC3922079

[12] Grunbacher A, Throm T, Seidel C, Gutt B, Rohrig J, Strunk T, et al. Six hydrophobins are involved in hydrophobin rodlet formation in *Aspergillus nidulans* and contribute to hydrophobicity of the spore surface. PLoS One. 2014;9(4):e94546. Epub 2014/04/12. doi: 10.1371/journal.pone.0094546 ; PubMed Central PMCID: PMC3983194. 24722460 PMC3983194

[13] Askolin S, Penttila M, Wosten HA, Nakari-Setala T. The *Trichoderma reesei* hydrophobin genes *hfb1* and *hfb2* have diverse functions in fungal development. FEMS Microbiol Lett. 2005;253(2):281–8. Epub 2005/10/26. doi: 10.1016/j.femsle.2005.09.047 . 16243453

[14] Bell-Pedersen D, Dunlap JC, Loros JJ. The *Neurospora* circadian clock-controlled gene, *ccg-2*, is allelic to *eas* and encodes a fungal hydrophobin required for formation of the conidial rodlet layer. Genes Dev. 1992;6(12A):2382–94. Epub 1992/12/01. doi: 10.1101/gad.6.12a.2382 . 1459460

[15] Krijgsheld P, Bleichrodt R, van Veluw GJ, Wang F, Muller WH, Dijksterhuis J, et al. Development in *Aspergillus*. Stud Mycol. 2013;74(1):1–29. Epub 2013/03/02. doi: 10.3114/sim0006 ; PubMed Central PMCID: PMC3563288. 23450714 PMC3563288

[16] Zajc J, Liu Y, Dai W, Yang Z, Hu J, Gostincar C, et al. Genome and transcriptome sequencing of the halophilic fungus *Wallemia ichthyophaga*: haloadaptations present and absent. BMC Genomics. 2013;14:617. Epub 2013/09/17. doi: 10.1186/1471-2164-14-617 ; PubMed Central PMCID: PMC3849046. 24034603 PMC3849046

[17] Rineau F, Lmalem H, Ahren D, Shah F, Johansson T, Coninx L, et al. Comparative genomics and expression levels of hydrophobins from eight mycorrhizal genomes. Mycorrhiza. 2017;27(4):383–96. Epub 2017/01/10. doi: 10.1007/s00572-016-0758-4 . 28066872

[18] Kubicek CP, Steindorff AS, Chenthamara K, Manganiello G, Henrissat B, Zhang J, et al. Evolution and comparative genomics of the most common *Trichoderma* species. BMC Genomics. 2019;20(1):485. Epub 2019/06/14. doi: 10.1186/s12864-019-5680-7 ; PubMed Central PMCID: PMC6560777. 31189469 PMC6560777

[19] Kubicek CP, Baker S, Gamauf C, Kenerley CM, Druzhinina IS. Purifying selection and birth-and-death evolution in the class II hydrophobin gene families of the ascomycete *Trichoderma*/*Hypocrea*. BMC Evol Biol. 2008;8:4. Epub 2008/01/12. doi: 10.1186/1471-2148-8-4 ; PubMed Central PMCID: PMC2253510. 18186925 PMC2253510

[20] Askolin S, Linder M, Scholtmeijer K, Tenkanen M, Penttila M, de Vocht ML, et al. Interaction and comparison of a class I hydrophobin from *Schizophyllum commune* and class II hydrophobins from *Trichoderma reesei*. Biomacromolecules. 2006;7(4):1295–301. Epub 2006/04/11. doi: 10.1021/bm050676s . 16602752

[21] Wessels JG. Hydrophobins: proteins that change the nature of the fungal surface. Adv Microb Physiol. 1997;38:1–45. Epub 1997/01/01. doi: 10.1016/s0065-2911(08)60154-x 8922117

[22] Jaklitsch WM, Voglmayr H. Biodiversity of *Trichoderma* (Hypocreaceae) in Southern Europe and Macaronesia. Stud Mycol. 2015;80:1–87. Epub 2016/03/10. doi: 10.1016/j.simyco.2014.11.001 ; PubMed Central PMCID: PMC4779795. 26955191 PMC4779795

[23] Chaverri P, Branco-Rocha F, Jaklitsch W, Gazis R, Degenkolb T, Samuels GJ. Systematics of the *Trichoderma harzianum* species complex and the re-identification of commercial biocontrol strains. Mycologia. 2015;107(3):558–90. Epub 2015/02/11. doi: 10.3852/14-147 ; PubMed Central PMCID: PMC4885665. 25661720 PMC4885665

[24] De Schutter K, Lin YC, Tiels P, Van Hecke A, Glinka S, Weber-Lehmann J, et al. Genome sequence of the recombinant protein production host *Pichia pastoris*. Nat Biotechnol. 2009;27(6):561–6. Epub 2009/05/26. doi: 10.1038/nbt.1544 . 19465926

[25] Joensuu JJ, Conley AJ, Lienemann M, Brandle JE, Linder MB, Menassa R. Hydrophobin fusions for high-level transient protein expression and purification in *Nicotiana benthamiana*. Plant Physiol. 2010;152(2):622–33. Epub 2009/12/19. doi: 10.1104/pp.109.149021 ; PubMed Central PMCID: PMC2815860. 20018596 PMC2815860

[26] Przylucka A, Akcapinar GB, Chenthamara K, Cai F, Grujic M, Karpenko J, et al. HFB7—A novel orphan hydrophobin of the *Harzianum* and *Virens* clades of *Trichoderma*, is involved in response to biotic and abiotic stresses. Fungal Genet Biol. 2017;102:63–76. Epub 2017/01/17. doi: 10.1016/j.fgb.2017.01.002 . 28089933

[27] Espino-Rammer L, Ribitsch D, Przylucka A, Marold A, Greimel KJ, Herrero Acero E, et al. Two novel class II hydrophobins from *Trichoderma* spp. stimulate enzymatic hydrolysis of poly(ethylene terephthalate) when expressed as fusion proteins. Appl Environ Microbiol. 2013;79(14):4230–8. Epub 2013/05/07. doi: 10.1128/AEM.01132-13 ; PubMed Central PMCID: PMC3697496. 23645195 PMC3697496

[28] Chanda A, Roze LV, Kang S, Artymovich KA, Hicks GR, Raikhel NV, et al. A key role for vesicles in fungal secondary metabolism. Proc Natl Acad Sci U S A. 2009;106(46):19533–8. Epub 2009/11/06. doi: 10.1073/pnas.0907416106 ; PubMed Central PMCID: PMC2773199. 19889978 PMC2773199

[29] Nordmann M, Cabrera M, Perz A, Brocker C, Ostrowicz C, Engelbrecht-Vandre S, et al. The Mon1-Ccz1 complex is the GEF of the late endosomal Rab7 homolog Ypt7. Curr Biol. 2010;20(18):1654–9. Epub 2010/08/28. doi: 10.1016/j.cub.2010.08.002 . 20797862

[30] Schuck S, Gallagher CM, Walter P. ER-phagy mediates selective degradation of endoplasmic reticulum independently of the core autophagy machinery. J Cell Sci. 2014;127(Pt 18):4078–88. Epub 2014/07/24. doi: 10.1242/jcs.154716 ; PubMed Central PMCID: PMC4163648. 25052096 PMC4163648

[31] Nykanen M, Birch D, Peterson R, Yu H, Kautto L, Gryshyna A, et al. Ultrastructural features of the early secretory pathway in *Trichoderma reesei*. Curr Genet. 2016;62(2):455–65. Epub 2015/12/25. doi: 10.1007/s00294-015-0555-1 . 26699139

[32] van Zutphen T, Todde V, de Boer R, Kreim M, Hofbauer HF, Wolinski H, et al. Lipid droplet autophagy in the yeast *Saccharomyces cerevisiae*. Mol Biol Cell. 2014;25(2):290–301. Epub 2013/11/22. doi: 10.1091/mbc.E13-08-0448 ; PubMed Central PMCID: PMC3890349. 24258026 PMC3890349

[33] Hahl H, Vargas JN, Griffo A, Laaksonen P, Szilvay G, Lienemann M, et al. Pure Protein Bilayers and Vesicles from Native Fungal Hydrophobins. Adv Mater. 2017;29(1). Epub 2016/10/16. doi: 10.1002/adma.201602888 . 27740699

[34] Lew RR. How does a hypha grow? The biophysics of pressurized growth in fungi. Nat Rev Microbiol. 2011;9(7):509–18. Epub 2011/06/07. doi: 10.1038/nrmicro2591 . 21643041

[35] van Wetter MA, Wosten HA, Sietsma JH, Wessels JG. Hydrophobin gene expression affects hyphal wall composition in *Schizophyllum commune*. Fungal Genet Biol. 2000;31(2):99–104. Epub 2001/02/15. doi: 10.1006/fgbi.2000.1231 . 11170739

[36] Lew RR, Levina NN, Shabala L, Anderca MI, Shabala SN. Role of a mitogen-activated protein kinase cascade in ion flux-mediated turgor regulation in fungi. Eukaryot Cell. 2006;5(3):480–7. Epub 2006/03/10. doi: 10.1128/EC.5.3.480-487.2006 ; PubMed Central PMCID: PMC1398064. 16524903 PMC1398064

[37] Bertl A, Slayman CL. Complex modulation of cation channels in the tonoplast and plasma membrane of *Saccharomyces cerevisiae*: single-channel studies. J Exp Biol. 1992;172:271–87. Epub 1992/11/01. 1283402 10.1242/jeb.172.1.271

[38] Andres Z, Perez-Hormaeche J, Leidi EO, Schlucking K, Steinhorst L, McLachlan DH, et al. Control of vacuolar dynamics and regulation of stomatal aperture by tonoplast potassium uptake. Proc Natl Acad Sci U S A. 2014;111(17):E1806–14. Epub 2014/04/16. doi: 10.1073/pnas.1320421111 ; PubMed Central PMCID: PMC4035970. 24733919 PMC4035970

[39] Gareis M, Gareis EM. Guttation droplets of *Penicillium nordicum* and *Penicillium verrucosum* contain high concentrations of the mycotoxins ochratoxin A and B. Mycopathologia. 2007;163(4):207–14. Epub 2007/04/04. doi: 10.1007/s11046-007-9003-1 . 17404894

[40] Hutwimmer S, Wang H, Strasser H, Burgstaller W. Formation of exudate droplets by *Metarhizium anisopliae* and the presence of destruxins. Mycologia. 2010;102(1):1–10. Epub 2010/02/03. doi: 10.3852/09-079 . 20120222

[41] Castagnoli E, Marik T, Mikkola R, Kredics L, Andersson MA, Salonen H, et al. Indoor *Trichoderma* strains emitting peptaibols in guttation droplets. J Appl Microbiol. 2018;125(5):1408–22. Epub 2018/05/21. doi: 10.1111/jam.13920 . 29779239

[42] Zhang J, Miao Y, Rahimi MJ, Zhu H, Steindorff A, Schiessler S, et al. Guttation capsules containing hydrogen peroxide: an evolutionarily conserved NADPH oxidase gains a role in wars between related fungi: The role of hydrogen peroxide in fungal wars. Environmental Microbiology. 2019. doi: 10.1111/1462-2920.14575 30815928 PMC6850483

[43] Wessels J, De Vries O, Asgeirsdottir SA, Schuren F. Hydrophobin Genes Involved in Formation of Aerial Hyphae and Fruit Bodies in *Schizophyllum*. Plant Cell. 1991;3(8):793–9. Epub 1991/08/01. doi: 10.1105/tpc.3.8.793 ; PubMed Central PMCID: PMC160046. 12324614 PMC160046

[44] Kontar S, Varecka L, Hires M, Krystofova S, Simkovic M. Light-induced conidiation of *Trichoderma* spp. strains is accompanied by development-dependent changes in the Ca(2+) binding to cell walls. Can J Microbiol. 2018;64(11):856–64. Epub 2018/06/16. doi: 10.1139/cjm-2017-0747 . 29906398

[45] Steyaert JM, Weld RJ, Loguercio LL, Stewart A. Rhythmic conidiation in the blue-light fungus *Trichoderma pleuroticola*. Fungal Biol. 2010;114(2–3):219–23. Epub 2010/10/15. doi: 10.1016/j.funbio.2010.01.001 . 20943132

[46] Osorio-Concepcion M, Cristobal-Mondragon GR, Gutierrez-Medina B, Casas-Flores S. Histone Deacetylase HDA-2 Regulates *Trichoderma atroviride* Growth, Conidiation, Blue Light Perception, and Oxidative Stress Responses. Appl Environ Microbiol. 2017;83(3). Epub 2016/11/20. doi: 10.1128/AEM.02922-16 ; PubMed Central PMCID: PMC5244289. 27864177 PMC5244289

[47] Meyer R, Parish RW, Hohl HR. Hyphal tip growth in *Phytophthora*. Gradient distribution and ultrahistochemistry of enzymes. Arch Microbiol. 1976;110(23):215–24. Epub 1976/11/02. doi: 10.1007/BF00690230 . 1015947

[48] Bastmeyer M, Deising HB, Bechinger C. Force exertion in fungal infection. Annu Rev Biophys Biomol Struct. 2002;31:321–41. Epub 2002/05/04. doi: 10.1146/annurev.biophys.31.091701.170951 . 11988473

[49] Cavinder B, Sikhakolli U, Fellows KM, Trail F. Sexual development and ascospore discharge in *Fusarium graminearum*. J Vis Exp. 2012;(61). Epub 2012/04/12. doi: 10.3791/3895 ; PubMed Central PMCID: PMC3460587. 22491175 PMC3460587

[50] Lew RR, Levina NN, Walker SK, Garrill A. Turgor regulation in hyphal organisms. Fungal Genet Biol. 2004;41(11):1007–15. Epub 2004/10/07. doi: 10.1016/j.fgb.2004.07.007 . 15465389

[51] Balmant W, Sugai-Guerios MH, Coradin JH, Krieger N, Furigo Junior A, Mitchell DA. A model for growth of a single fungal hypha based on well-mixed tanks in series: simulation of nutrient and vesicle transport in aerial reproductive hyphae. PLoS One. 2015;10(3):e0120307. Epub 2015/03/19. doi: 10.1371/journal.pone.0120307 ; PubMed Central PMCID: PMC4364911. 25785863 PMC4364911

[52] Jaklitsch WM. European species of *Hypocrea* Part I. The green-spored species. Stud Mycol. 2009;63:1–91. Epub 2009/10/15. doi: 10.3114/sim.2009.63.01 ; PubMed Central PMCID: PMC2757427. 19826500 PMC2757427

[53] Jaklitsch WM. European species of *Hypocrea* part II: species with hyaline ascospores. Fungal Divers. 2011;48(1):1–250. Epub 2011/10/14. doi: 10.1007/s13225-011-0088-y ; PubMed Central PMCID: PMC3189789. 21994484 PMC3189789

[54] Lugones LG, Bosscher JS, Scholtmeyer K, de Vries OM, Wessels JG. An abundant hydrophobin (ABH1) forms hydrophobic rodlet layers in *Agaricus bisporus* fruiting bodies. Microbiology. 1996;142 (Pt 5):1321–9. Epub 1996/05/01. doi: 10.1099/13500872-142-5-1321 . 8704971

[55] Stringer MA, Dean RA, Sewall TC, Timberlake WE. Rodletless, a new *Aspergillus* developmental mutant induced by directed gene inactivation. Genes Dev. 1991;5(7):1161–71. Epub 1991/07/01. doi: 10.1101/gad.5.7.1161 . 2065971

[56] Simon A, Bindschedler S, Job D, Wick LY, Filippidou S, Kooli WM, et al. Exploiting the fungal highway: development of a novel tool for the in situ isolation of bacteria migrating along fungal mycelium. FEMS Microbiol Ecol. 2015;91(11). Epub 2015/10/04. doi: 10.1093/femsec/fiv116 . 26432804

[57] Moonjely S, Keyhani NO, Bidochka MJ. Hydrophobins contribute to root colonization and stress responses in the rhizosphere-competent insect pathogenic fungus *Beauveria bassiana*. Microbiology. 2018;164(4):517–28. Epub 2018/03/09. doi: 10.1099/mic.0.000644 . 29517481

[58] Quarantin A, Hadeler B, Kroger C, Schafer W, Favaron F, Sella L, et al. Different Hydrophobins of *Fusarium graminearum* Are Involved in Hyphal Growth, Attachment, Water-Air Interface Penetration and Plant Infection. Front Microbiol. 2019;10:751. Epub 2019/04/30. doi: 10.3389/fmicb.2019.00751 ; PubMed Central PMCID: PMC6474331. 31031728 PMC6474331

[59] Wessels JG, de Vries OM, Asgeirsdottir SA, Springer J. The thn mutation of *Schizophyllum commune*, which suppresses formation of aerial hyphae, affects expression of the *Sc3* hydrophobin gene. J Gen Microbiol. 1991;137(10):2439–45. Epub 1991/10/01. doi: 10.1099/00221287-137-10-2439 . 1770359

[60] Sammer D, Krause K, Gube M, Wagner K, Kothe E. Hydrophobins in the Life Cycle of the Ectomycorrhizal Basidiomycete *Tricholoma vaccinum*. PLoS One. 2016;11(12):e0167773. Epub 2016/12/10. doi: 10.1371/journal.pone.0167773 ; PubMed Central PMCID: PMC5147985. 27936063 PMC5147985

[61] Oka OB, Bulleid NJ. Forming disulfides in the endoplasmic reticulum. Biochim Biophys Acta. 2013;1833(11):2425–9. Epub 2013/02/26. doi: 10.1016/j.bbamcr.2013.02.007 . 23434683

[62] Yan Y, Wang H, Zhu S, Wang J, Liu X, Lin FC, et al. The methylcitrate cycle is required for development and virulence in the rice blast fungus *Pyricularia oryzae*. Mol Plant Microbe Interact. 2019. Epub 2019/04/02. doi: 10.1094/MPMI-10-18-0292-R . 30933666

[63] Winandy L, Hilpert F, Schlebusch O, Fischer R. Comparative analysis of surface coating properties of five hydrophobins from *Aspergillus nidulans* and *Trichoderma reseei*. Sci Rep. 2018;8(1):12033. Epub 2018/08/15. doi: 10.1038/s41598-018-29749-0 ; PubMed Central PMCID: PMC6089913. 30104653 PMC6089913

[64] Zhang X, Blalock B, Huberty W, Chen Y, Hung F, Russo PS. Microbubbles and Oil Droplets Stabilized by a Class II Hydrophobin in Marinelike Environments. Langmuir. 2019;35(12):4380–6. Epub 2019/03/16. doi: 10.1021/acs.langmuir.8b03777 . 30873841

[65] Hahl H, Vargas JN, Jung M, Griffo A, Laaksonen P, Lienemann M, et al. Adhesion Properties of Freestanding Hydrophobin Bilayers. Langmuir. 2018;34(29):8542–9. Epub 2018/06/12. doi: 10.1021/acs.langmuir.8b00575 . 29886739

[66] Maas SL, Broekman ML, de Vrij J. Tunable Resistive Pulse Sensing for the Characterization of Extracellular Vesicles. Methods Mol Biol. 2017;1545:21–33. Epub 2016/12/13. doi: 10.1007/978-1-4939-6728-5_2 . 27943204

[67] de Toledo Martins S, Szwarc P, Goldenberg S, Alves LR. Extracellular Vesicles in Fungi: Composition and Functions. Curr Top Microbiol Immunol. 2019;422:45–59. Epub 2018/09/23. doi: 10.1007/82_2018_141 . 30242512

[68] de Paula RG, Antonieto ACC, Nogueira KMV, Ribeiro LFC, Rocha MC, Malavazi I, et al. Extracellular vesicles carry cellulases in the industrial fungus *Trichoderma reesei*. Biotechnol Biofuels. 2019;12:146. Epub 2019/06/22. doi: 10.1186/s13068-019-1487-7 ; PubMed Central PMCID: PMC6570945. 31223336 PMC6570945

[69] Kurylowicz W, Kurzatkowski W, Woznicka W, Polowniak-Pracka H, Paszkiewicz A. The ultrastructure of *Penicillium chrysogenum* in the course of benzyl-penicillin biosynthesis. Zentralbl Bakteriol Naturwiss. 1979;134(8):706–20. Epub 1979/01/01. doi: 10.1016/s0323-6056(79)80031-2 120749

[70] Cole GTH, C.H. The Fungal Spore and Disease Initiation in Plants and Animals: Springer; 1991.

[71] Ivanov S, Austin J 2nd, Berg RH, Harrison MJ. Extensive membrane systems at the host-arbuscular mycorrhizal fungus interface. Nat Plants. 2019;5(2):194–203. Epub 2019/02/10. doi: 10.1038/s41477-019-0364-5 . 30737512

[72] Yafetto L, Carroll L, Cui Y, Davis DJ, Fischer MW, Henterly AC, et al. The fastest flights in nature: high-speed spore discharge mechanisms among fungi. PLoS One. 2008;3(9):e3237. Epub 2008/09/18. doi: 10.1371/journal.pone.0003237 ; PubMed Central PMCID: PMC2528943. 18797504 PMC2528943

[73] Jensen BG, Andersen MR, Pedersen MH, Frisvad JC, Sondergaard I. Hydrophobins from *Aspergillus* species cannot be clearly divided into two classes. BMC Res Notes. 2010;3:344. Epub 2010/12/25. doi: 10.1186/1756-0500-3-344 ; PubMed Central PMCID: PMC3020181. 21182770 PMC3020181

[74] Jaklitsch WM, Voglmayr H. Biodiversity of *Trichoderma* (Hypocreaceae) in Southern Europe and Macaronesia. Studies in Mycology. 2015;80:1–87. doi: 10.1016/j.simyco.2014.11.001 26955191 PMC4779795

[75] Schmittgen TD, Livak KJ. Analyzing real-time PCR data by the comparative C(T) method. Nat Protoc. 2008;3(6):1101–8. Epub 2008/06/13. doi: 10.1038/nprot.2008.73 . 18546601

[76] Zhang J, Miao Y, Rahimi MJ, Zhu H, Steindorff A, Schiessler S, et al. Guttation capsules containing hydrogen peroxide: an evolutionarily conserved NADPH oxidase gains a role in wars between related fungi. Environ Microbiol. 2019;21(8):2644–58. Epub 2019/03/01. doi: 10.1111/1462-2920.14575 . 30815928 PMC6850483

[77] Uzbas F, Sezerman U, Hartl L, Kubicek CP, Seiboth B. A homologous production system for *Trichoderma reesei* secreted proteins in a cellulase-free background. Appl Microbiol Biotechnol. 2012;93(4):1601–8. Epub 2011/11/15. doi: 10.1007/s00253-011-3674-8 ; PubMed Central PMCID: PMC3275749. 22080343 PMC3275749

[78] Seiboth B, Karimi RA, Phatale PA, Linke R, Hartl L, Sauer DG, et al. The putative protein methyltransferase LAE1 controls cellulase gene expression in *Trichoderma reesei*. Mol Microbiol. 2012;84(6):1150–64. Epub 2012/05/05. doi: 10.1111/j.1365-2958.2012.08083.x ; PubMed Central PMCID: PMC3370264. 22554051 PMC3370264

[79] Trinh R, Gurbaxani B, Morrison SL, Seyfzadeh M. Optimization of codon pair use within the (GGGGS)3 linker sequence results in enhanced protein expression. Mol Immunol. 2004;40(10):717–22. Epub 2003/12/04. doi: 10.1016/j.molimm.2003.08.006 . 14644097

[80] Byrne B. *Pichia pastoris* as an expression host for membrane protein structural biology. Curr Opin Struct Biol. 2015;32:9–17. Epub 2015/02/07. doi: 10.1016/j.sbi.2015.01.005 . 25658849

[81] Druzhinina IS, Schmoll M, Seiboth B, Kubicek CP. Global Carbon Utilization Profiles of Wild-Type, Mutant, and Transformant Strains of *Hypocrea jecorina*. Applied and Environmental Microbiology. 2006;72(3):2126–33. doi: 10.1128/AEM.72.3.2126-2133.2006 16517662 PMC1393202

[82] Atanasova L, Druzhinina IS. Review: Global nutrient profiling by Phenotype MicroArrays: a tool complementing genomic and proteomic studies in conidial fungi. J Zhejiang Univ Sci B. 2010;11(3):151–68. Epub 2010/03/06. doi: 10.1631/jzus.B1000007 ; PubMed Central PMCID: PMC2833400. 20205302 PMC2833400

