# Supplementary material for: The pleiotropic functions of intracellular hydrophobins in aerial hyphae and fungal spores
Source: PLoS Genet. 2021 Nov 17;17(11):e1009924. doi: 10.1371/journal.pgen.1009924 (PMC8635391; doi:10.1371/journal.pgen.1009924)
Supplement: S1 Fig — (PDF) [file pgen.1009924.s001.pdf]

Supporting Information S1 Fig. Principal component analysis (PCA) of the *hfb* expression pattern during *Trichoderma* development

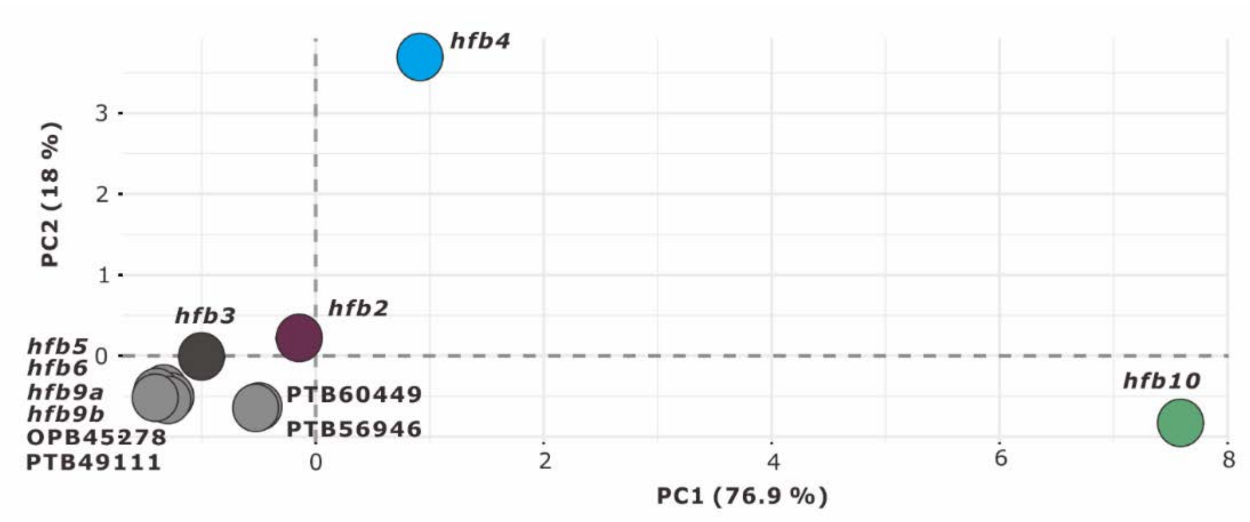

Fig S1 Principal component analysis (PCA) of the *hfb* expression pattern in *T. harzianum* and *T. guizhouense*. The complete list of *hfb*-encoding genes and their transcriptional profile are given in Table 1.
