# Supplementary material for: The pleiotropic functions of intracellular hydrophobins in aerial hyphae and fungal spores
Source: PLoS Genet. 2021 Nov 17;17(11):e1009924. doi: 10.1371/journal.pgen.1009924 (PMC8635391; doi:10.1371/journal.pgen.1009924)
Supplement: S2 Fig — (PDF) [file pgen.1009924.s002.pdf]

Supporting Information S2 Fig. Molecular dynamic simulation and solvent accessible surface area calculation of fluorescently tagged HFBs used in this study

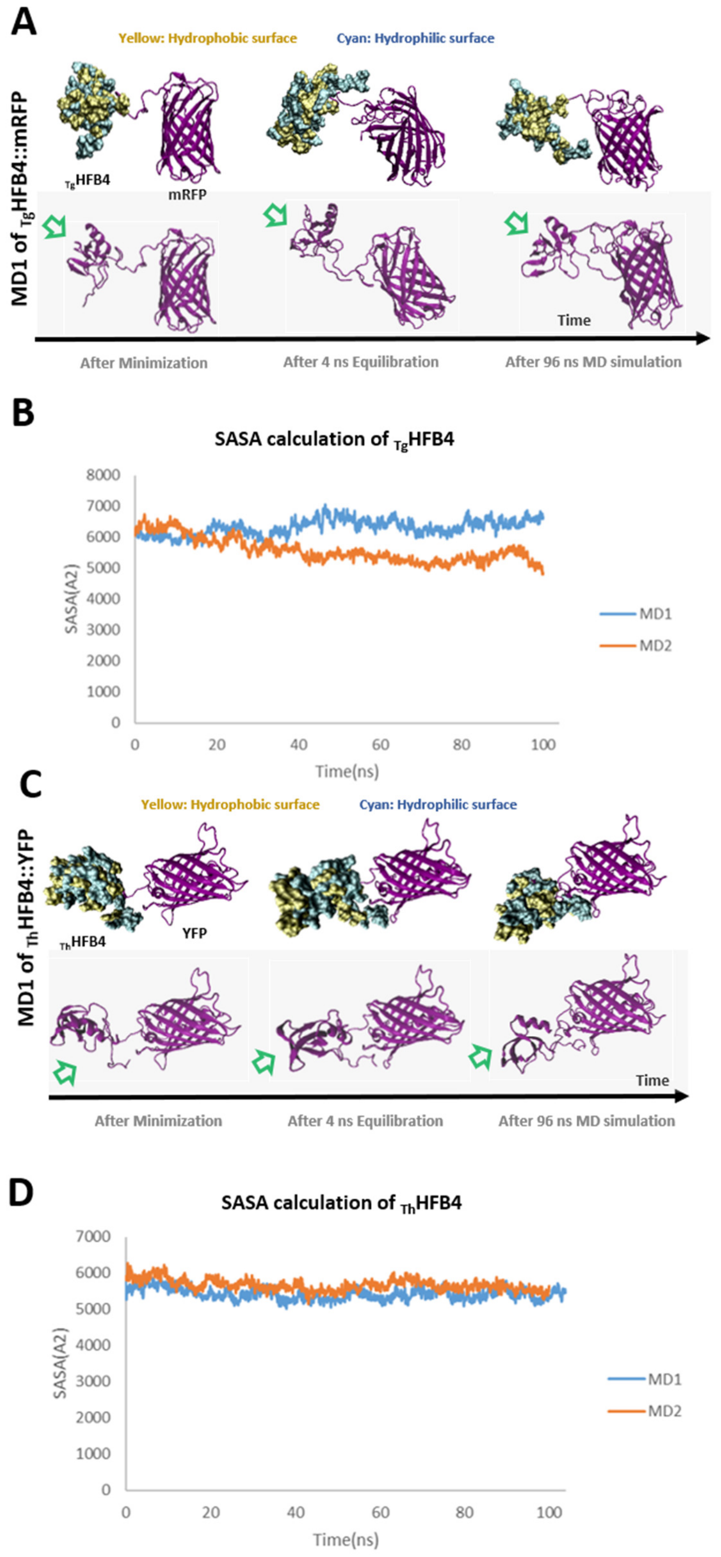

Fig S2 Molecular dynamics (MD, **A** and **C**) simulation and solvent accessible surface area (SASA, **B** and **D**) calculation showed no putative disrupting effect of the fluorescent tags on the studied HFB4. MD simulations were used to analyze the effects of the fusion partners (mRFP and YFP) on the HFB4 protein structures ($_{Tg}$HFB4 and $_{Th}$HFB4), respectively, by the NAMD/VMD software package [1]. Simulations were performed inside a 10 Å water box under periodic boundary conditions at 298 °K using TIP3P water. All the structures were neutralized by the addition of Na^+ or Cl^- ions. A 2 fs timestep was used, and data collection was performed every 2 ps. Modeled structures were minimized in 50000 steps using the conjugate gradient (CG) method before the simulations. MD simulations of 100 ns (4 ns equilibration and 96 ns production runs) were performed at 298 °K using the NPT ensemble under constant pressure and temperature. The root mean square deviation (RMSD) is adopted to indicate the large structural changes in the protein and to measure the scalar distance between atoms of the same type for two structures [2]. SASA is used to calculate the surface area of an atom, a residue, and a molecule that is exposed to a specific solvent. This factor is measured in angstroms2 (Å2)[2]. SASAs of the two fusion proteins ($_{Tg}$HFB4::mRFP and $_{Th}$HFB4::YFP) were calculated for each fusion partner along the simulation time from the trajectories of the MD simulations performed at 298 K using a water sphere with a radius of 1.4 Å via VMD scripting. Homology modeling was performed using Modeler9v23 [3, 4] based on the HFBII (=HFB2) structure deposited in the protein databank (PDB ID: 1R2M-Chain A) from *T. reesei* for $_{Tg}$HFB4 and $_{Th}$HFB4 and the mCherry structure (PDB ID: 6B0B-Chain D) and GFP structure (PDB ID: 4Xl5-Chain A) for mRFP and YFP, respectively. Ten different models were generated for each fusion protein and compared. The best scoring models are selected for the subsequent MD simulation. Note: MD was performed with two repeat runs (MD1 and MD2), and here, only MD1 is shown as a representative example. Green arrows point to the hydrophobic patch of HFBs.

References

1. Phillips JC, Braun R, Wang W, Gumbart J, Tajkhorshid E, Villa E, et al. Scalable molecular dynamics with NAMD. J Comput Chem. 2005;26(16):1781-802. Epub 2005/10/14. doi: 10.1002/jcc.20289. PubMed PMID: 16222654; PubMed Central PMCID: PMCPMC2486339.
2. Knapp B, Lederer N, Omasits U, Schreiner W. vmdICE: a plug-in for rapid evaluation of molecular dynamics simulations using VMD. J Comput Chem. 2010;31(16):2868-73. Epub 2010/10/12. doi: 10.1002/jcc.21581. PubMed PMID: 20928849.
3. Sali A, Potterton L, Yuan F, van Vlijmen H, Karplus M. Evaluation of comparative protein modeling by MODELLER. Proteins. 1995;23(3):318-26. Epub 1995/11/01. doi: 10.1002/prot.340230306. PubMed PMID: 8710825.
4. Fiser A, Sali A. Modeller: generation and refinement of homology-based protein structure models. Methods Enzymol. 2003;374:461-91. Epub 2003/12/31. doi: 10.1016/S0076-6879(03)74020-8. PubMed PMID: 14696385.
