# Supplementary material for: The pleiotropic functions of intracellular hydrophobins in aerial hyphae and fungal spores
Source: PLoS Genet. 2021 Nov 17;17(11):e1009924. doi: 10.1371/journal.pgen.1009924 (PMC8635391; doi:10.1371/journal.pgen.1009924)
Supplement: S3 Fig — (PDF) [file pgen.1009924.s003.pdf]

Supporting Information S3 Fig. Phenotypic characterization of HFB mutants

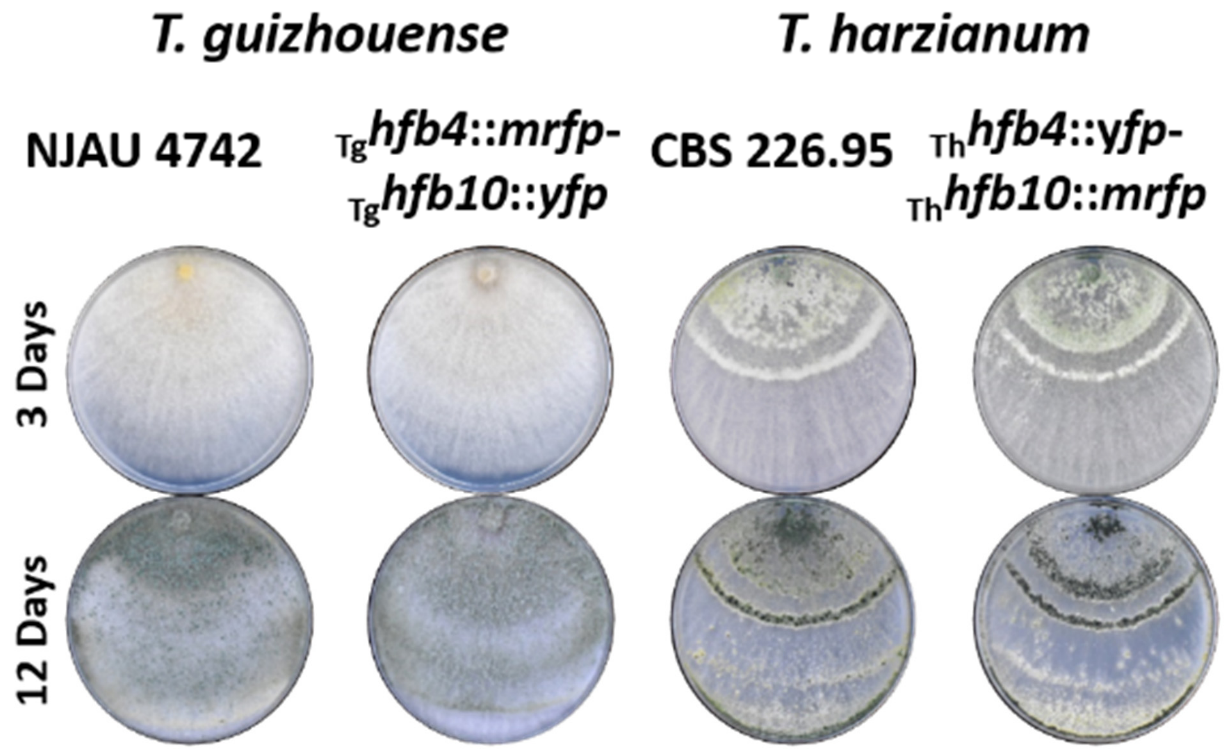

Fig A Representative morphology of the wild-type strains of *Trichoderma* (*T. guizhouense* NJAU 4742 and *T. harzianum* CBS 226.95) and their respective *hfb4* and *hfb10* double-labeled mutants used in this study. *Trichoderma* strains grown on PDA at 25 °C.

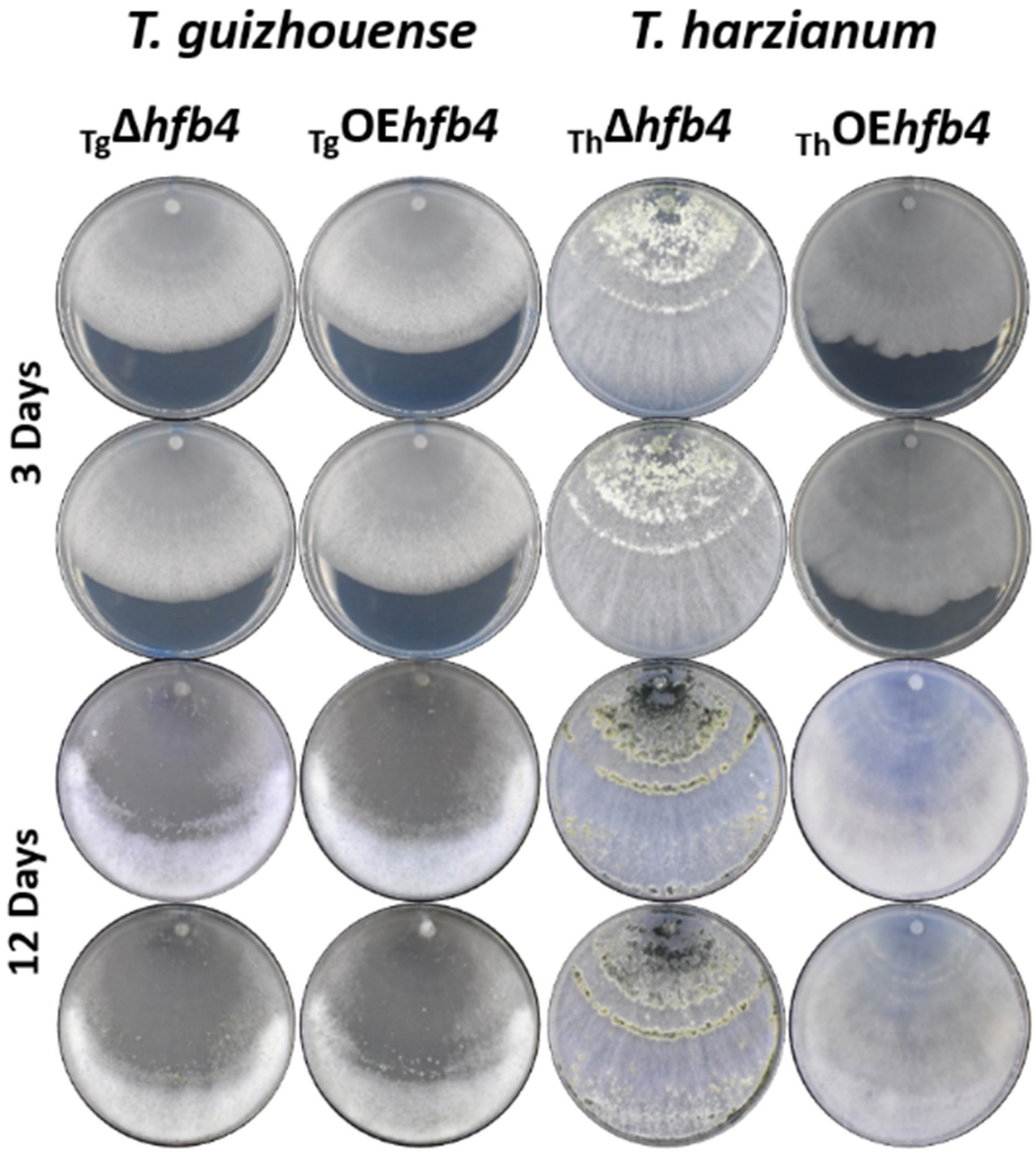

Fig B Representative morphology of *Trichoderma hfb4* mutants (N=2 for each genotype) generated in this study. *Trichoderma* strains grown on PDA at 25 °C.

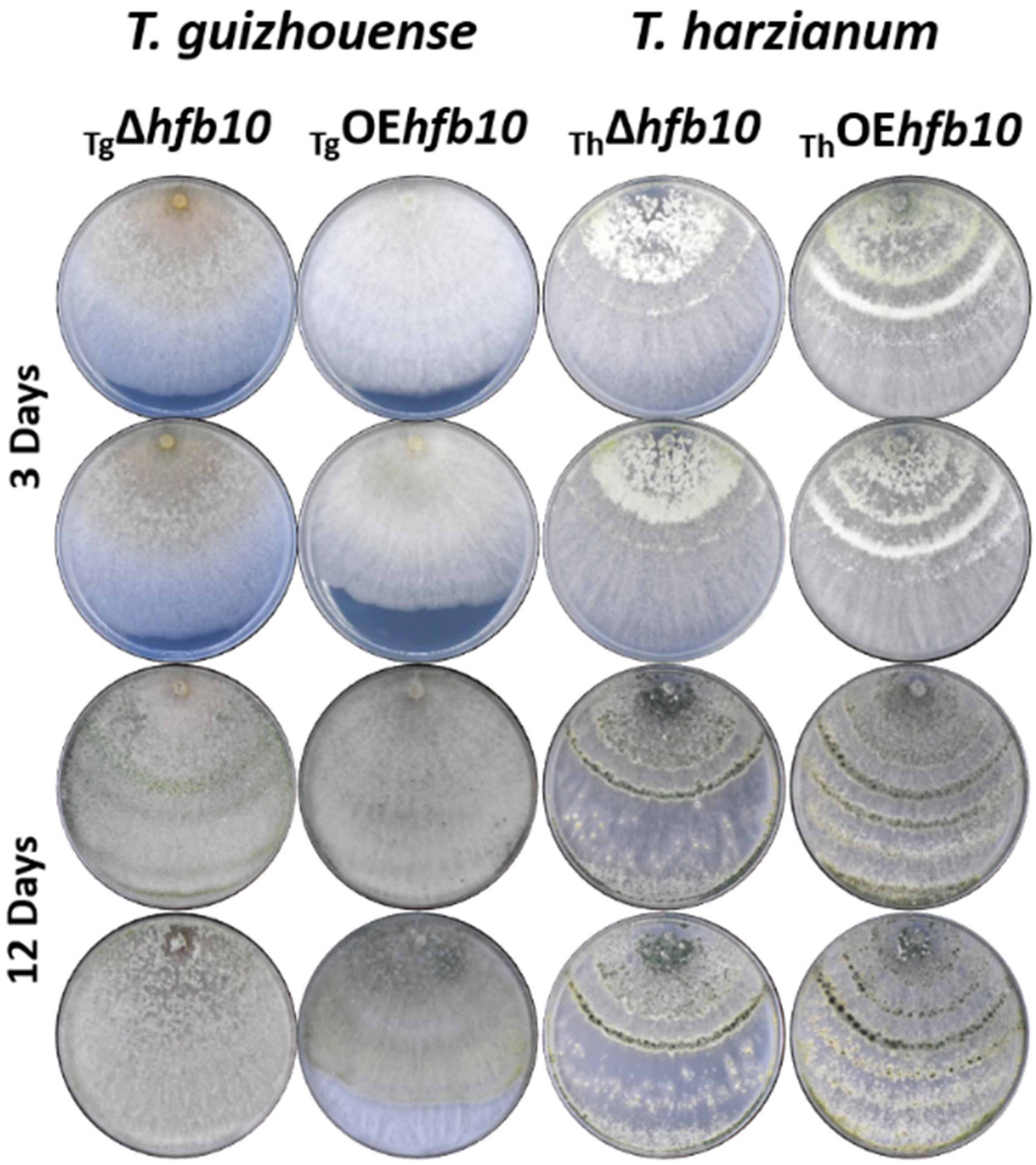

Fig C Representative morphology of *Trichoderma hfb10* mutants (N=2 for each genotype) generated in this study. *Trichoderma* strains grown on PDA at 25 °C.

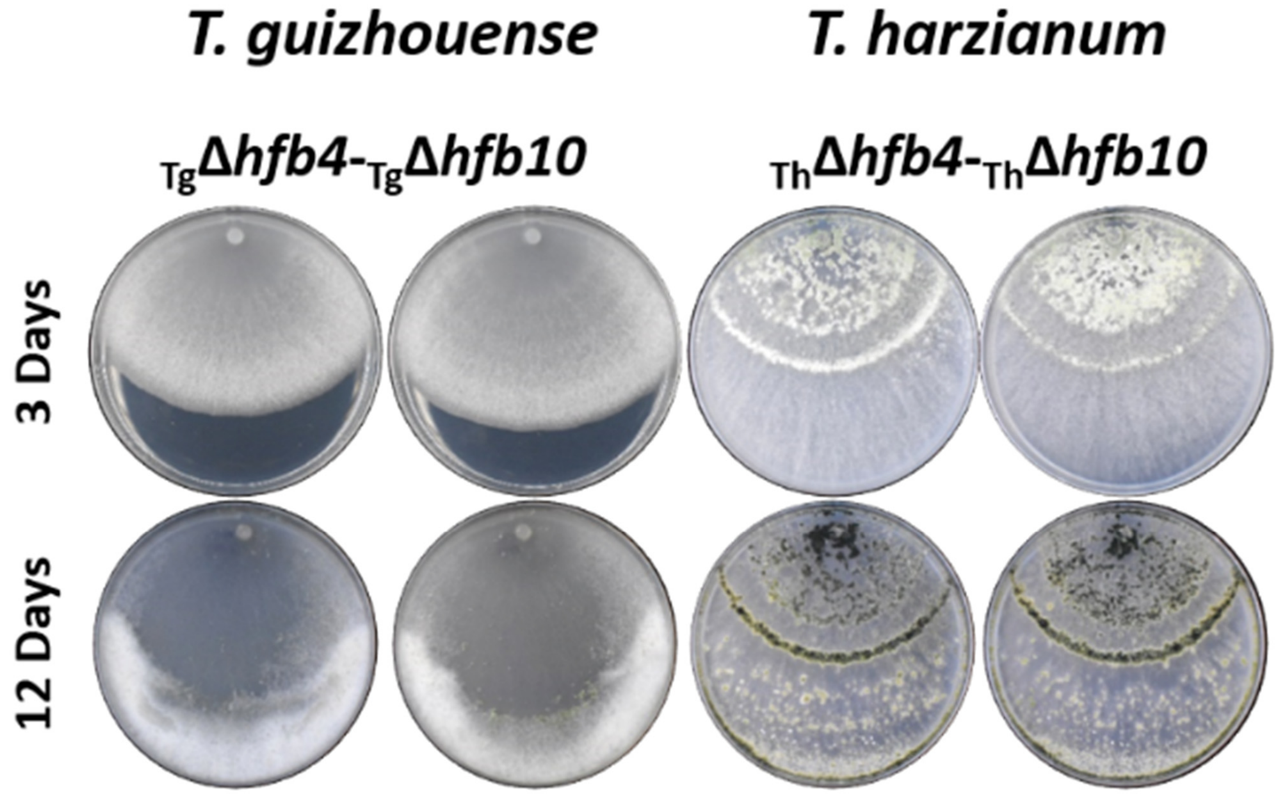

Fig D Representative morphology of *Trichoderma hfb4* and *hfb10* double deletion mutants (N=2 for each genotype) generated in this study. *Trichoderma* strains grown on PDA at 25 °C.

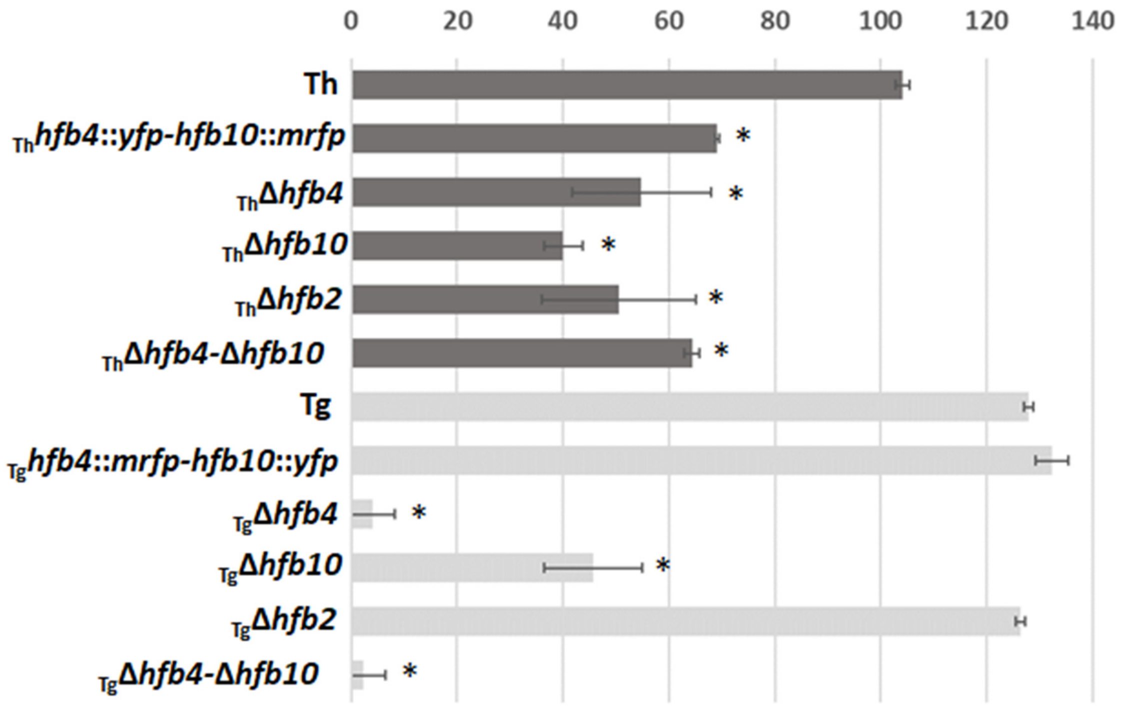

Fig E Surface hydrophobicity of *Trichoderma* hyphae expressed as the water contact angle (WCA) measured by an OCA 20 (DataPhysics, Filderstadt, Germany). The WCA determination was carried on the surfaces of young hyphae grown on PDA plates for 48 h in darkness. Bars with a * represent a significant (ANOVA, $p < 0.05$) difference of surface hydrophobicity measured for at least two mutants and compared the wild type strain. Fungal strains are abbreviated as given in Table 2.

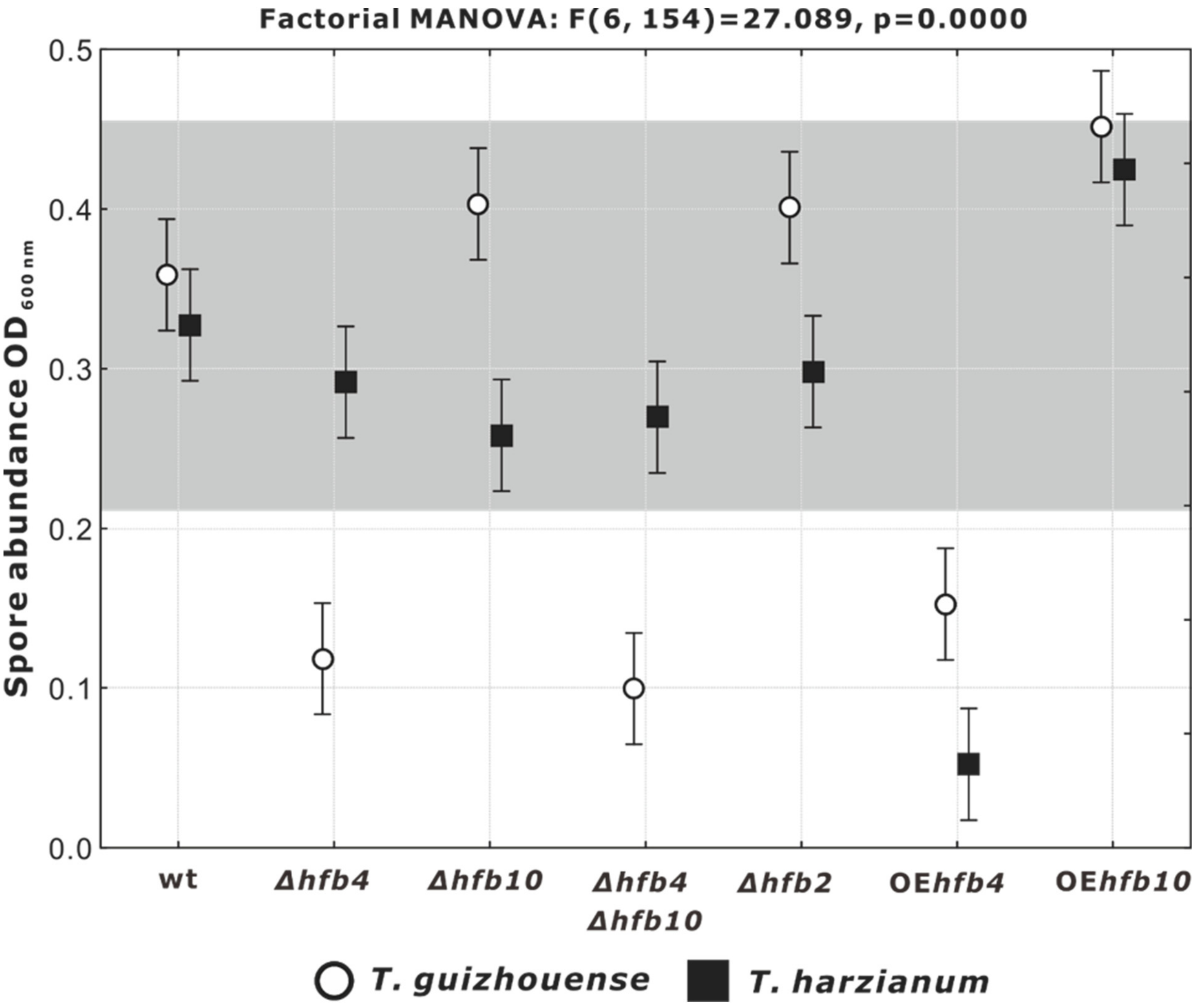

Fig F Statistical analysis of the conidiation abundance of *Trichoderma hfb* mutants. Surface of the PDA plate (6 cm diam.) cultivated for 14 d in darkness was washed thoroughly with sterile water. Spores were filtered through a double-layer gauze and collected by centrifugation at 8000 rpm min^{-1} for 5 min. The resulting pellet was resuspended in 1 ml of sterile water and OD was measured on SpectraMax iD 3 (Molecular Devices, USA) microplate reader at 600 nm after a proper dilution. Each symbol represents values measured for at least three plates per each of two mutants per haplotype. Vertical bars show 95 % confidence interval. Fungal strains are abbreviated as given in Table 2.
