# Supplementary material for: The pleiotropic functions of intracellular hydrophobins in aerial hyphae and fungal spores
Source: PLoS Genet. 2021 Nov 17;17(11):e1009924. doi: 10.1371/journal.pgen.1009924 (PMC8635391; doi:10.1371/journal.pgen.1009924)
Supplement: S4 Fig — (PDF) [file pgen.1009924.s004.pdf]

Supporting Information S4 Fig. Intracellular accumulation of mRFP-labeled HFB4 in conidiophores

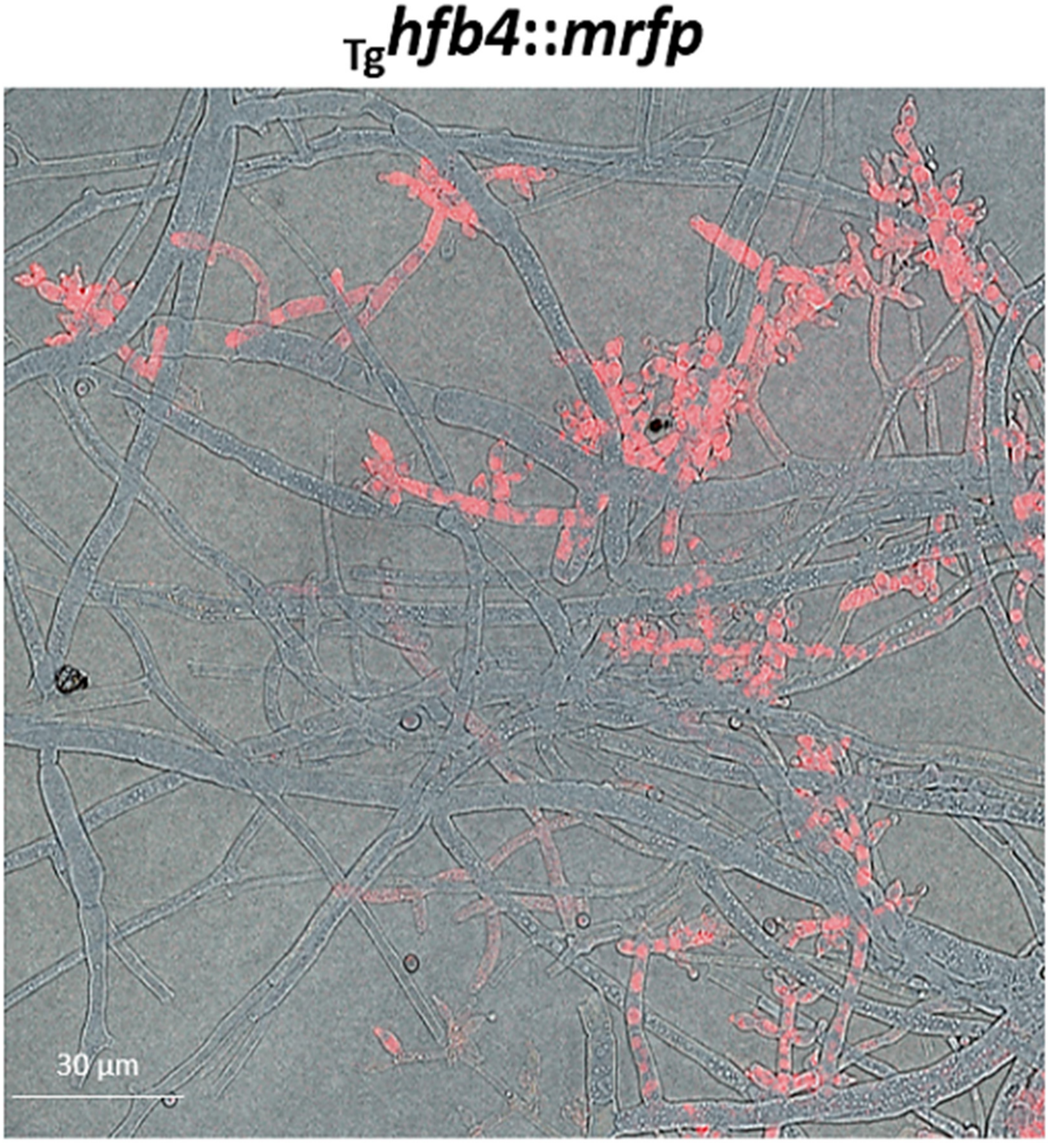

Fig S4 Intracellular accumulation of mRFP-labeled HFB4 in conidiophores of the $_{Tg}$*hfb4*::*mrfp* mutant cultivated on cellophane-covered PDA at 25 °C in darkness. The cellophane covered by hyphae was observed under an epifluorescence microscope (Leica DMi8 microscope, Germany) without water added.
