# Supplementary material for: The pleiotropic functions of intracellular hydrophobins in aerial hyphae and fungal spores
Source: PLoS Genet. 2021 Nov 17;17(11):e1009924. doi: 10.1371/journal.pgen.1009924 (PMC8635391; doi:10.1371/journal.pgen.1009924)
Supplement: S5 Fig — (PDF) [file pgen.1009924.s005.pdf]

Supporting Information S5 Fig. Localization of $_{Tg}$HFB3::mRFP in vacuole-resembling organelles (white arrows) and on the surface of phialides near the collarette (black arrows).

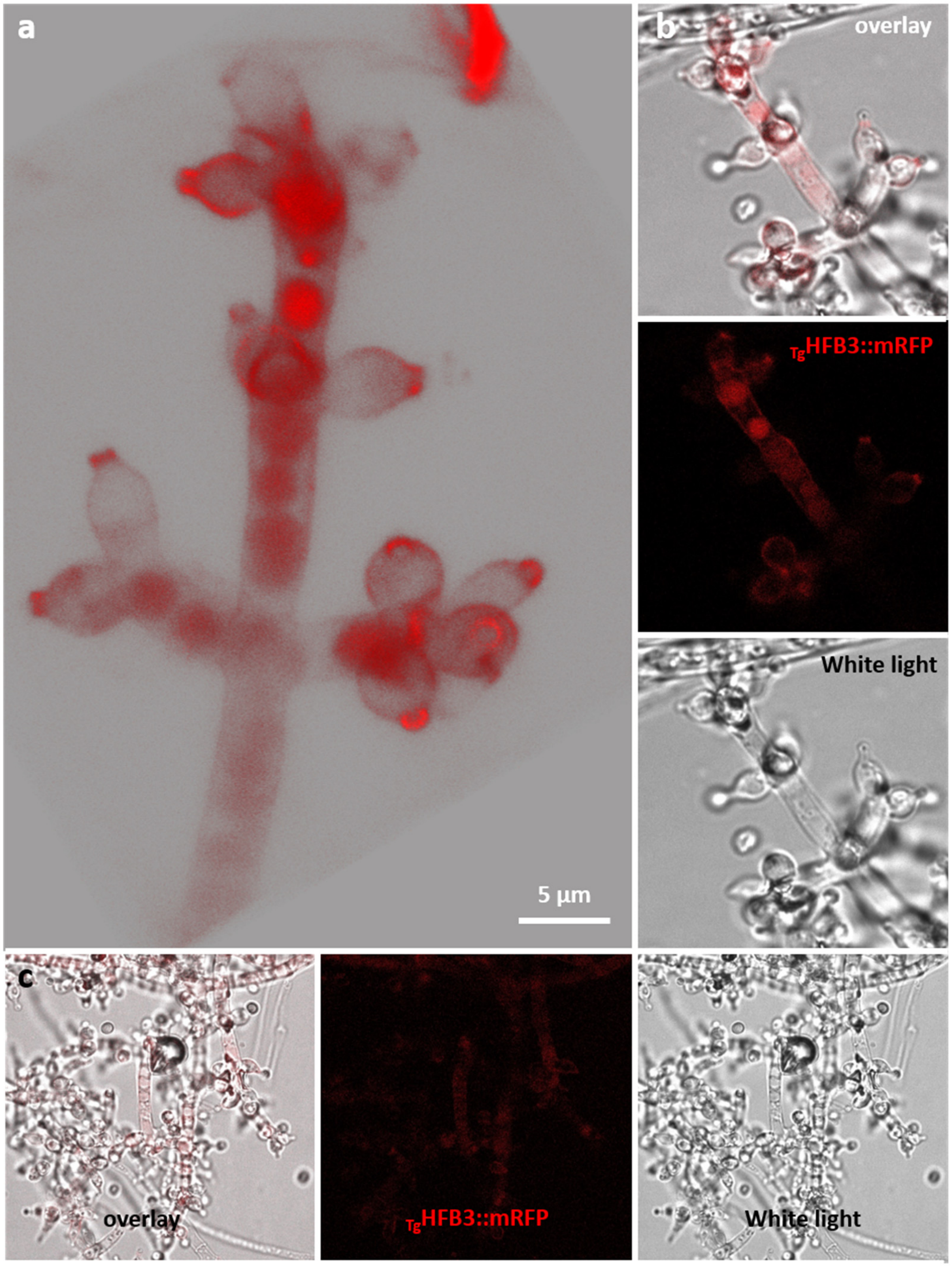

Fig S5 Localization of $_{Tg}$HFB3::mRFP expressed under the control of its native promoter. A A 3D reconstruction from the Z stack images of a conidiophore shown in B. HFB3::mRFP is seen in vacuole-like organelles inside the conidiophore and on the surface of phialides, in particular in the areas of phialide necks. Spores were not coated by HFB3::mRFP. C shows the low level of HFB3 secretion and association with conidiophores.
