# Supplementary material for: The pleiotropic functions of intracellular hydrophobins in aerial hyphae and fungal spores
Source: PLoS Genet. 2021 Nov 17;17(11):e1009924. doi: 10.1371/journal.pgen.1009924 (PMC8635391; doi:10.1371/journal.pgen.1009924)
Supplement: S6 Fig — (PDF) [file pgen.1009924.s006.pdf]

Supporting Information S6 Fig. Secretion of mRFP expressed using the signal peptide under the control of the promoter of $_{Tg}$*hfb4*

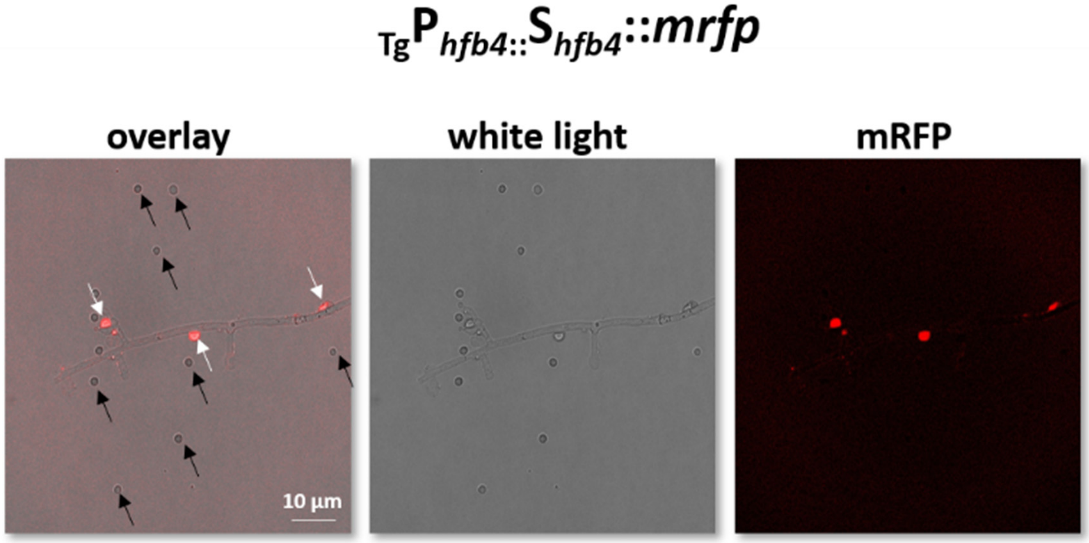

Fig S6 Secretion of mRFP expressed using the signal peptide and the promoter of the *hfb4* gene from *T. guizhouense* NJAU 4742. Microscopic analysis of the 48 h-old culture grown on PDA at 25 °C in darkness; white arrows point to the water/air interface (on air bubbles); black arrows point to spores, Leica DMi8 microscope (Leica, Germany)

This analysis demonstrates that mRFP has no affinity to intracellular accumulation in *Trichoderma* spp.
