# Supplementary material for: The pleiotropic functions of intracellular hydrophobins in aerial hyphae and fungal spores
Source: PLoS Genet. 2021 Nov 17;17(11):e1009924. doi: 10.1371/journal.pgen.1009924 (PMC8635391; doi:10.1371/journal.pgen.1009924)
Supplement: S7 Fig — (PDF) [file pgen.1009924.s007.pdf]

Supporting Information S7 Fig. Morphology and ultrastructure of *Komagataella pastoris* (Saccharomycetales, syn. *Pichia pastoris*) expressing *Trichoderma* HFB-encoding genes.

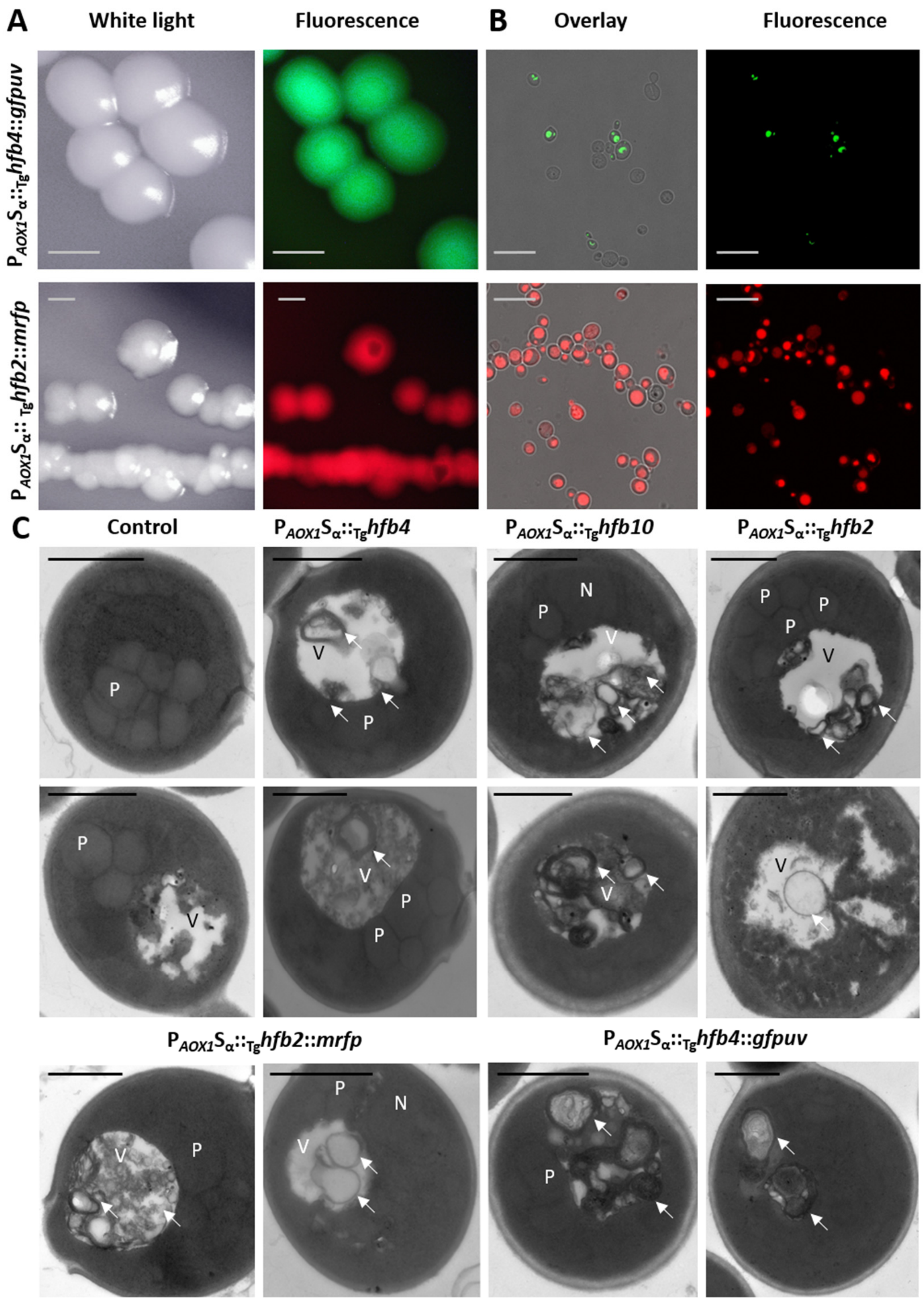

Fig A (A) Stereo confocal microscopy *of P. pastoris* producing fluorescently labeled HFBs from *Trichoderma* using the signal peptide of *Saccharomyces cerevisiae* α-mating factor (Sα) under the control of the AOX1 promoter (P_{AOX1}). *P. pastoris* were cultivated on BMM agar plates for four days, and HFB expression was induced by methanol. Scale bar = 5 mm. (B) Intracellular accumulation of HFBs in the same strains as in (A) fermented in BMM liquid medium. Scale bar = 10 µm. (C) TEM micrographs of the *P. pastoris* mutants overexpressing HFBs shown in (B). The control corresponds to the strain transformed with the HFB-free vector (pPICZαA); see the Materials and Methods for the details. P – peroxisome, V – vacuole, N – nucleus. Arrows point to putative HFB vesicles in VMSs that correspond to HFB-rich organelles in (B). Representative images were selected from total 139 images. Samples for TEM were prepared with at least two mutants and 15 images studied per each haplotype.

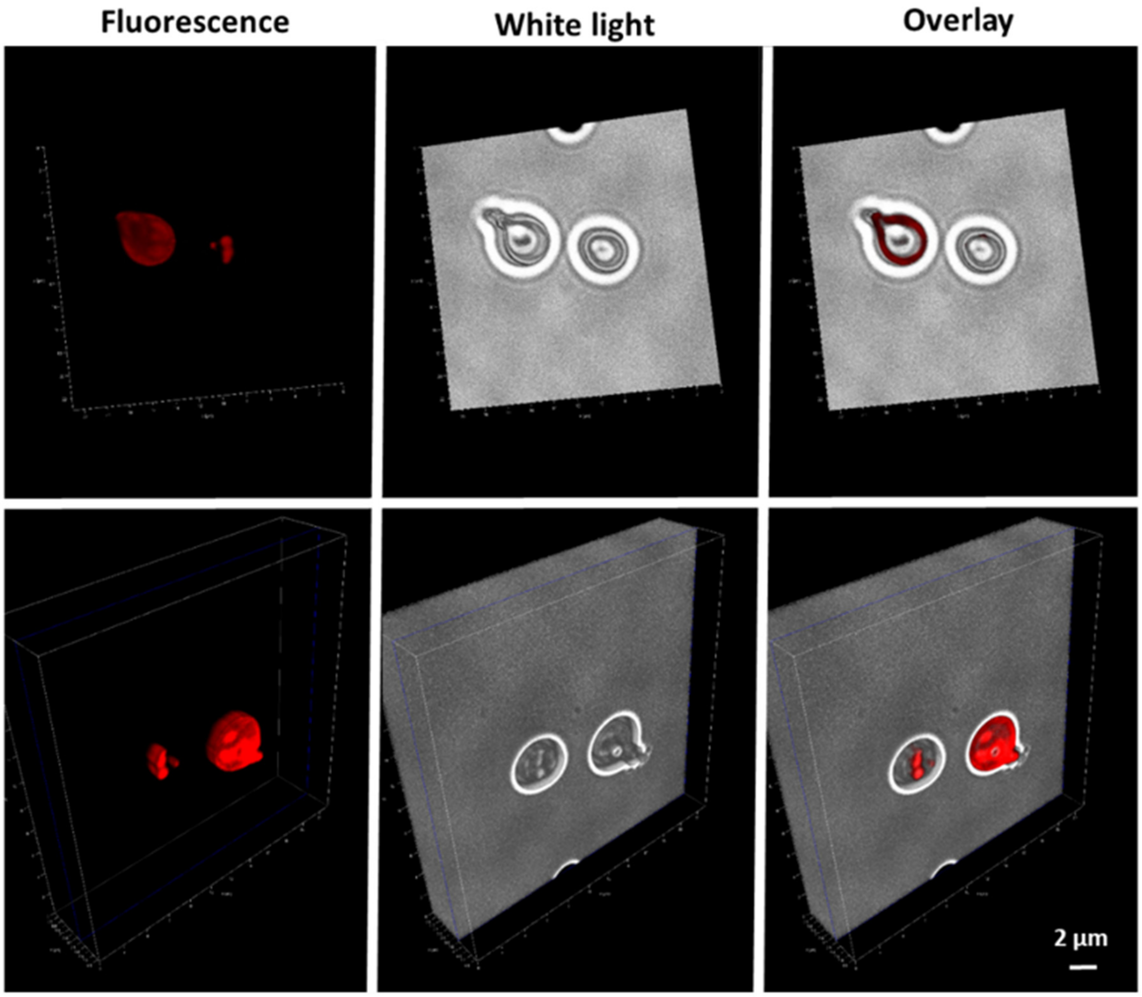

Fig B 3D visualization of HFB intracellular accumulation in the *P. pastoris* strain $P_{aox1}S_{\alpha}$::$_{Tg}$*hfb2*::*mrfp* overexpressing $_{Tg}$*hfb2* was imaged using a Leica DMi8 microscope (Leica, Germany). The strain was cultivated in BMM medium for HFB induction by 0.5% methanol (see the detailed procedure in Material & Method). mRFP was fused to the C-terminus of $_{Tg}$HFB2 for protein localization.
