# Supplementary material for: The pleiotropic functions of intracellular hydrophobins in aerial hyphae and fungal spores
Source: PLoS Genet. 2021 Nov 17;17(11):e1009924. doi: 10.1371/journal.pgen.1009924 (PMC8635391; doi:10.1371/journal.pgen.1009924)
Supplement: S8 Fig — (PDF) [file pgen.1009924.s008.pdf]

Supporting Information S8 Fig. Representative morphology of *Trichoderma* mutants lacking *rab7*

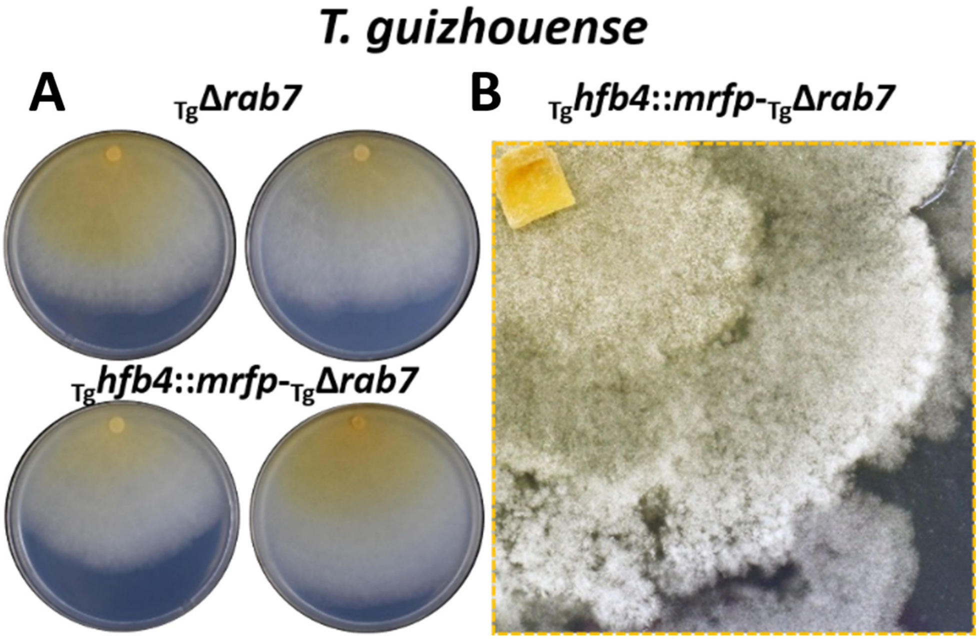

Fig A Representative morphology of *Trichoderma Δrab7* mutants (N=2 for each genotype) generated in this study (14 d). (A), *Trichoderma* strains grown on PDA at 25 °C in darkness; (B), a close-up image of the colony cultivated under the same conditions for 14 d.

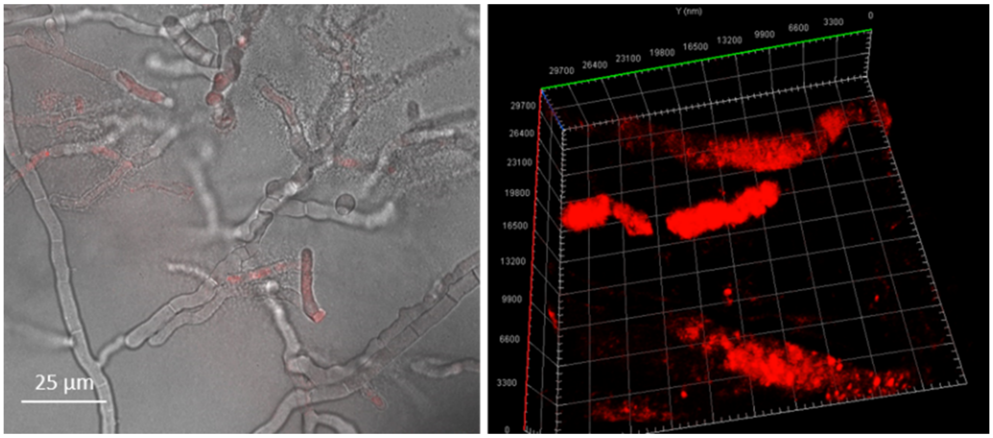

Fig B left: Representative morphology of $_{Tg}$*hfb4*::*mrfp-Δrab7* mutants (N=2 for each genotype observed under an epifluorescence microscope when an agar plug was placed on a cover glass. Right: A fluorescence overlay image is shown on the left, and a 3D reconstruction of the superresolution CLSM image.

$_{Tg}$***hfb4::mrfp-Δrab7***

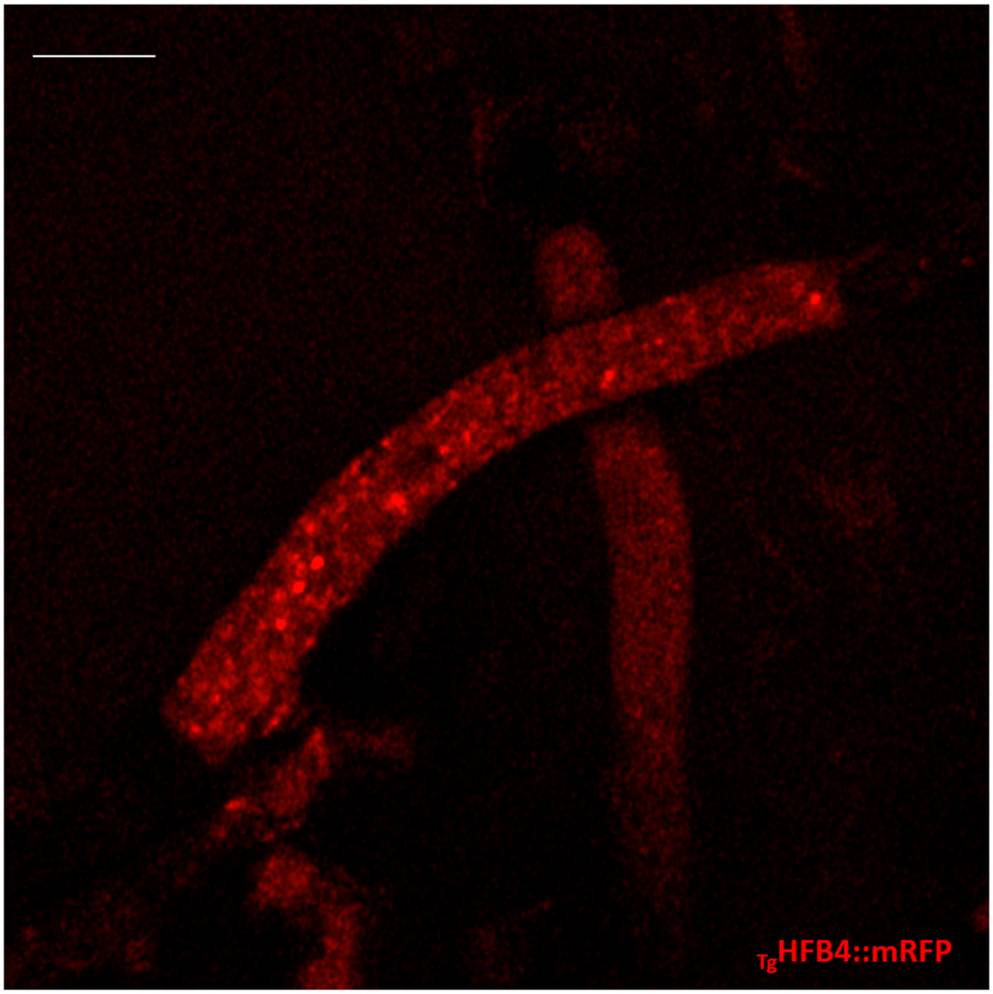

Fig C A 3D reconstruction of the aerial hyphae of the *Trichoderma guizhouense* mutant producing HFB4::mRFP but lacking the key protein for the cytoplasm-to-vacuole sorting pathway, RAB7 (homologous to YPT7 in *S. cerevisiae*). Scale bar= 10 μm.
