# Supplementary material for: The pleiotropic functions of intracellular hydrophobins in aerial hyphae and fungal spores
Source: PLoS Genet. 2021 Nov 17;17(11):e1009924. doi: 10.1371/journal.pgen.1009924 (PMC8635391; doi:10.1371/journal.pgen.1009924)
Supplement: S9 Fig — (PDF) [file pgen.1009924.s009.pdf]

Supporting Information S9 Fig. Fluorescent microscopy imaging and ultrastructure of *Trichoderma* spores

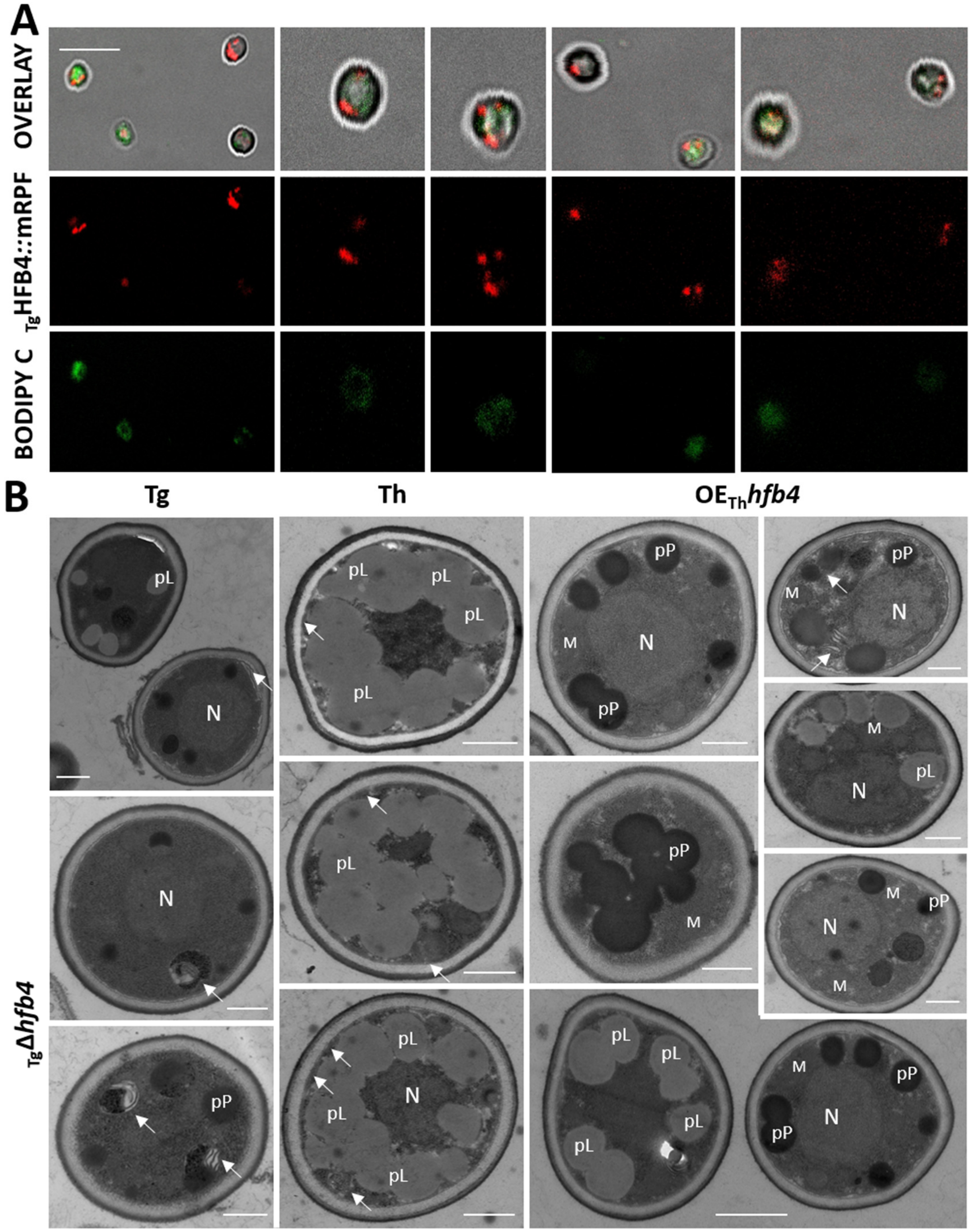

Fig S9 (A) Fluorescent images of the phospholipid-specific stained [using a green fluorescent fatty acid (BODIPYTM FL C12)] aerial hyphal tips of the *T. guizhouense* mutant producing HFB4::mRFP. Scale bar= 5 µm. (B) TEM images of mature conidia produced by the wild-type, HFB deletion, and HBF-overexpressing mutants of *Trichoderma* spp. N – nucleus, pL – putative lipid droplet, pP – putative proteinous inclusion, M – mitochondrion. Arrows point to the putative vacuolar multicisternal structures (VMS). Scale bar= 1 µm.
