# Supplementary material for: The pleiotropic functions of intracellular hydrophobins in aerial hyphae and fungal spores
Source: PLoS Genet. 2021 Nov 17;17(11):e1009924. doi: 10.1371/journal.pgen.1009924 (PMC8635391; doi:10.1371/journal.pgen.1009924)
Supplement: S10 Fig — (PDF) [file pgen.1009924.s010.pdf]

Supporting Information S10 Fig. Morphology of $_{Tg}$OE*hfb2*::*mrfp* and immunochemical characterization of HFB2 secreted by aerial hyphae.

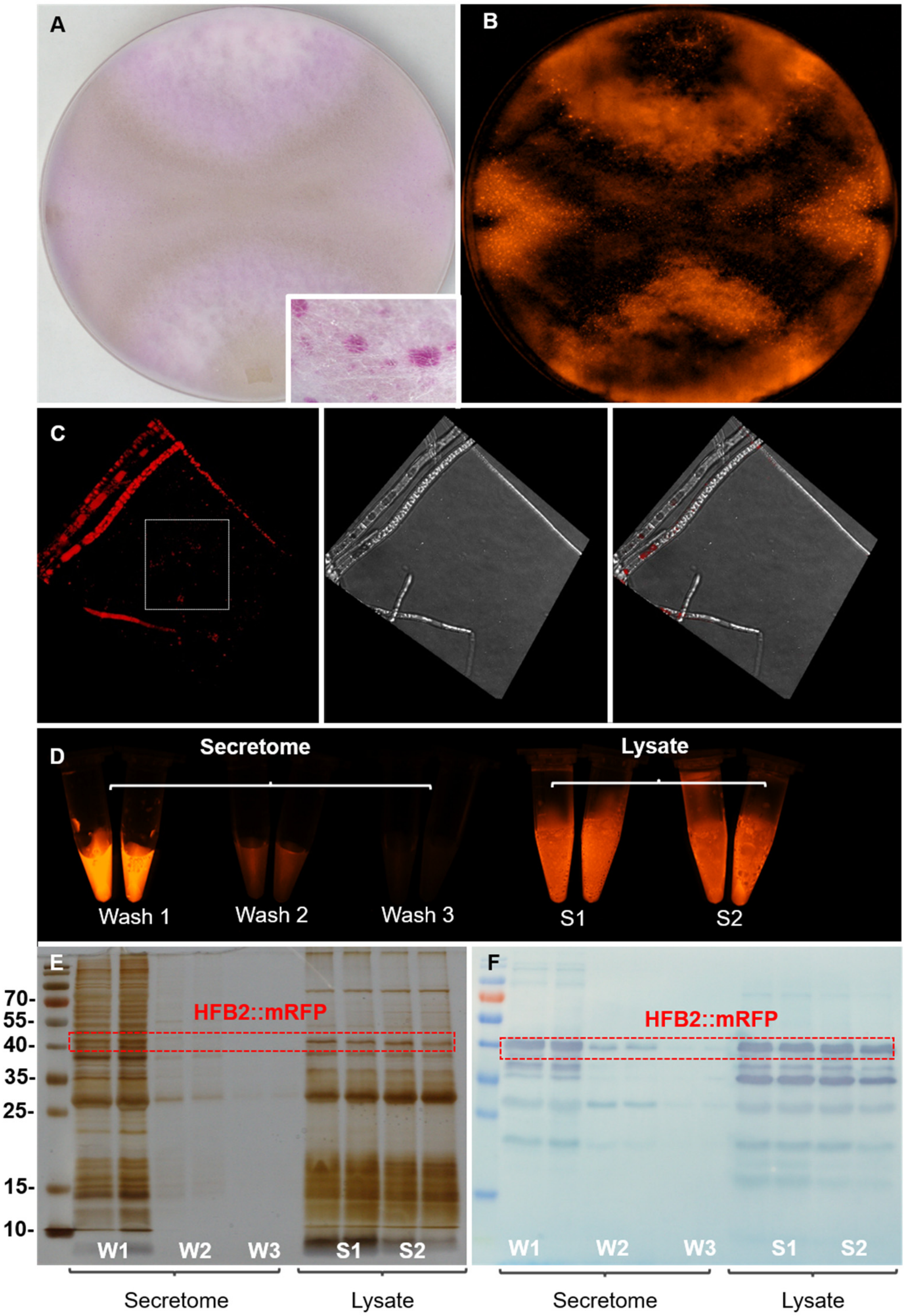

Fig S10 Immunochemical characterization of HFB2 secreted by aerial hyphae of the "pink strain" $_{Tg}$OE*hfb2*::*mrfp*. **(A)** Morphology of the strain after three weeks of incubation at 25 °C in darkness. The

insert in (A) shows a close-up view of the aerial hyphae. Note: the aerial mycelium of the wild-type *T. guizhouense* strain lives up to 10-14 days and then becomes autolyzed. (B) The same plate was imaged by a Bio-Rad ChemiDoc MP (Bio-Rad, USA) equipped with multiplex fluorescent channels. Bright dots correspond to guttation droplets filled with HFB2::mRFP fusion protein seen in the insert in A. (C) Intra- and extracellular HFB-enriched vesicles. Extracellular vesicles are abundantly observed if the hyphae of $_{\mathrm{Tg}}$OE*hfb2*::*mrfp* mechanically disrupted on the microscopy slide. (D) Qualitative detection of fluorescence (of HFB2::mRFP) in samples collected from the secretome and the lysate of culture shown in (A). The secretome samples (W1-W3) were collected by washing the 21-d-old PDA culture three times with HPLC water. The lysate samples (S1-S2) were prepared by lysing the water-washed aerial hyphae two times by 1% SDS (dissolved in 50 mM Tris-HCl, pH 8.1). (E) and (F), SDS-PAGE and immune blotting (WB) confirmation of the protein samples shown in (A) – (D).
