# Supplementary material for: The pleiotropic functions of intracellular hydrophobins in aerial hyphae and fungal spores
Source: PLoS Genet. 2021 Nov 17;17(11):e1009924. doi: 10.1371/journal.pgen.1009924 (PMC8635391; doi:10.1371/journal.pgen.1009924)

Supporting Information S11 Fig. Full size TEM micrographs used for the photoplate in Fig 3.

Representative TEM micrographs showing the hyphal ultrastructure of strains including the wild-type strain of *T. guizhouense* (Tg), HFB2::mRFP-overexpressing strains of Tg, HFB4-overexpressing *T. harzianum* (Th), or hyphae of Tg and Th lacking HFB4 and HFB10. All images of the aerial hyphae were obtained from a two-day-old colony. Representative images were selected from a total of 695 images obtained for Tg (361) and Th (334). Samples for TEM were prepared with at least two mutants and 30 images studied per genotype. Mutants are listed in Table 2.

Tg

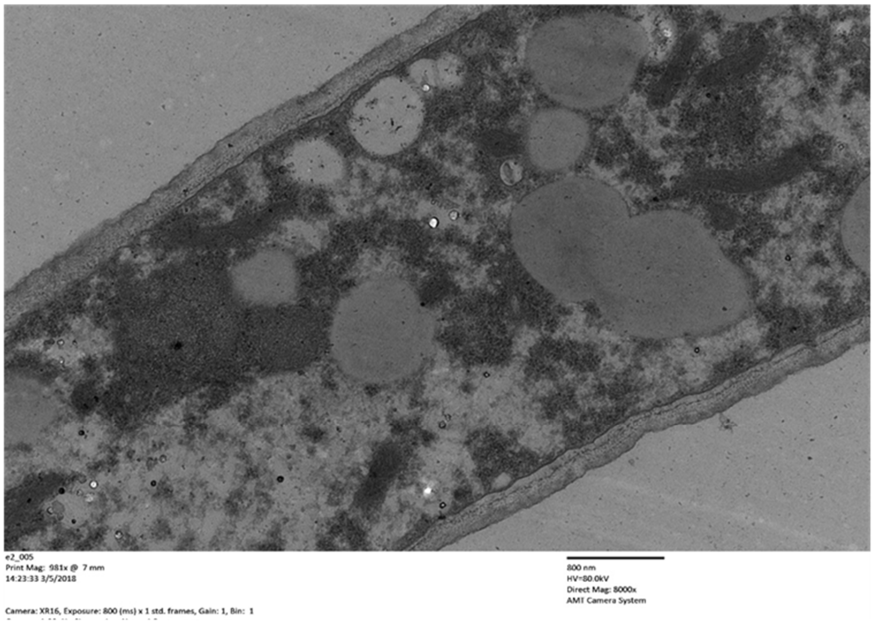

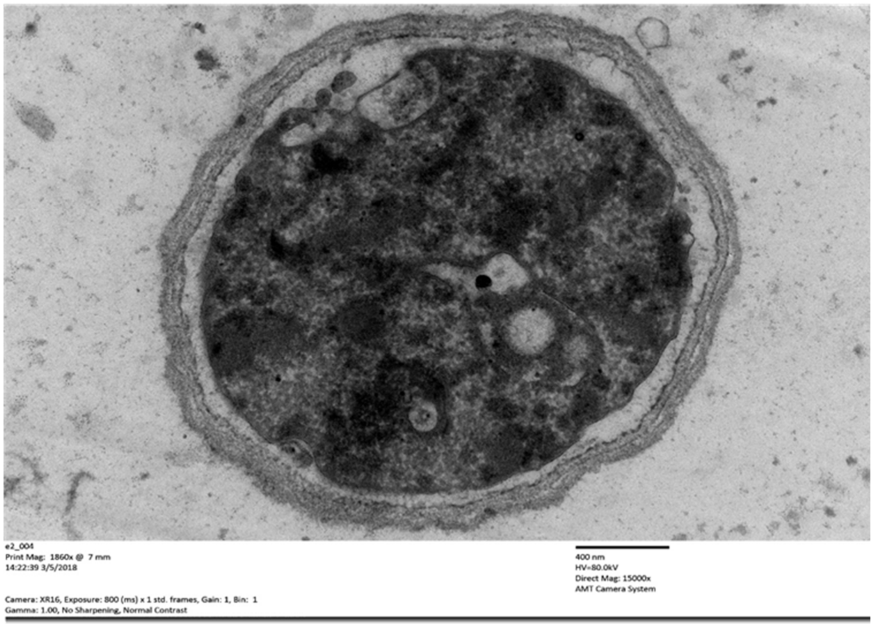

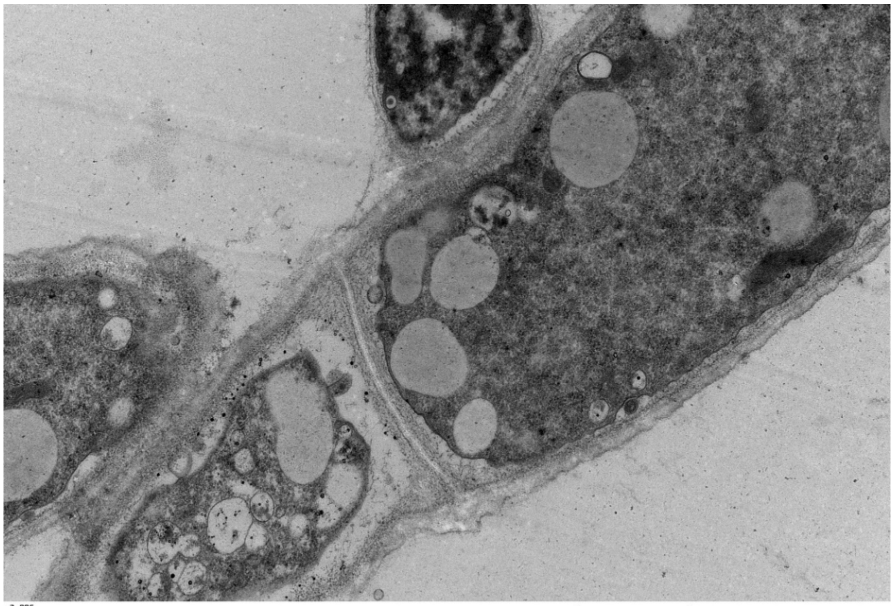

e2_006
Print Mag: 862x @ 7 mm
14:25:46 3/5/2018

1 µm
HV=80.0kV
Direct Mag: 7000x
AMT Camera System

Camera: XR16, Exposure: 800 (ms) x 1 std. frames, Gain: 1, Bin: 1
Gamma: 1.00, No Sharpening, Normal Contrast

$_{Tg}$OE*hfb2*::*mrfp*

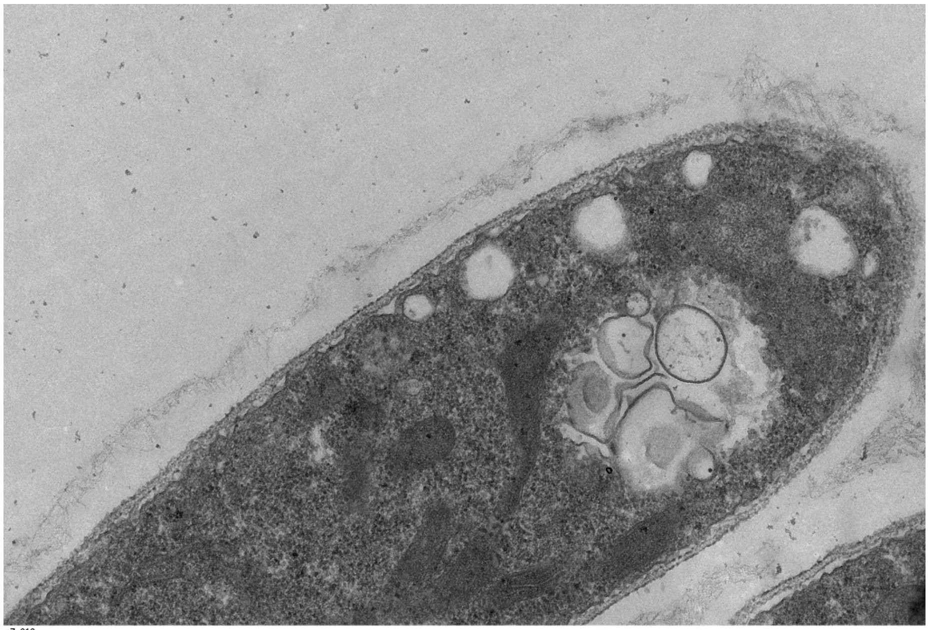

e7_010
Print Mag: 1460x @ 7 mm
15:27:59 3/5/2018

500 nm
HV=80.0kV
Direct Mag: 12000x
AMT Camera System

Camera: XR16, Exposure: 800 (ms) x 1 std. frames, Gain: 1, Bin: 1
Gamma: 1.00, No Sharpening, Normal Contrast

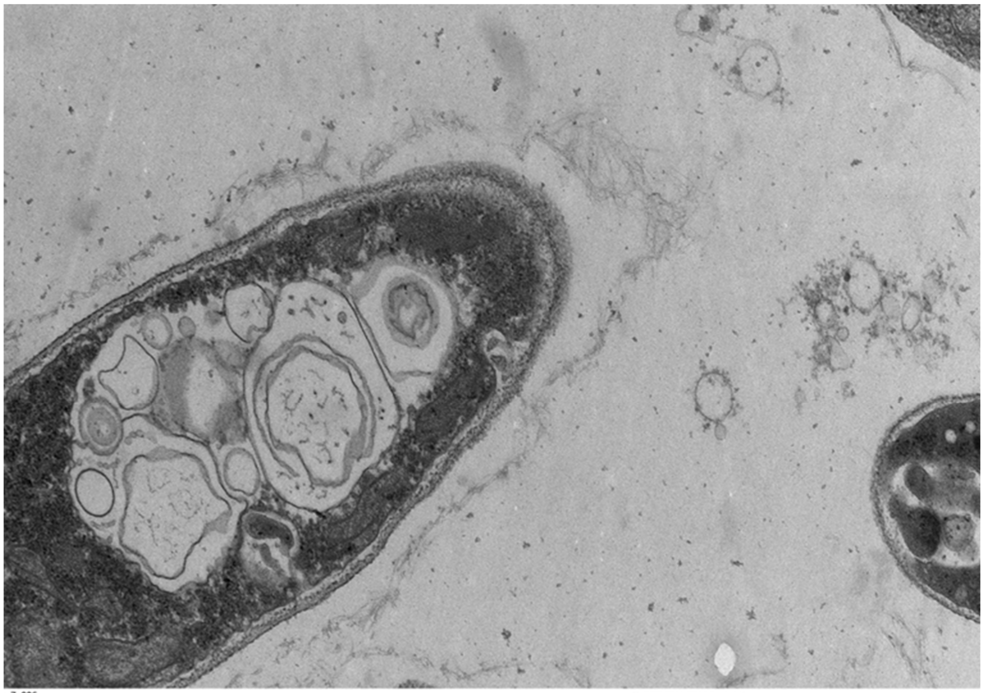

e7_006
Print Mag: 1210x @ 7 mm
15:23:44 3/5/2018

600 nm
HV=80.0kV
Direct Mag: 10000x
AMT Camera System

Camera: XR16, Exposure: 800 (ms) x 1 std. frames, Gain: 1, Bin: 1
Gamma: 1.00, No Sharpening, Normal Contrast

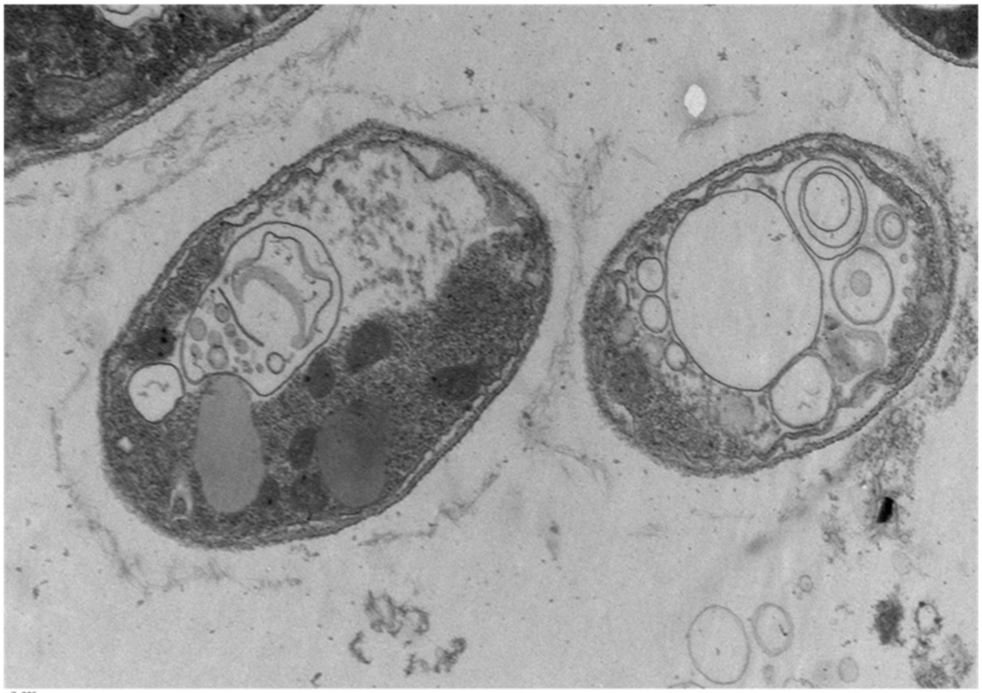

e7_005
Print Mag: 1210x @ 7 mm
15:23:16 3/5/2018

600 nm
HV=80.0kV
Direct Mag: 10000x
AMT Camera System

Camera: XR16, Exposure: 800 (ms) x 1 std. frames, Gain: 1, Bin: 1
Gamma: 1.00, No Sharpening, Normal Contrast

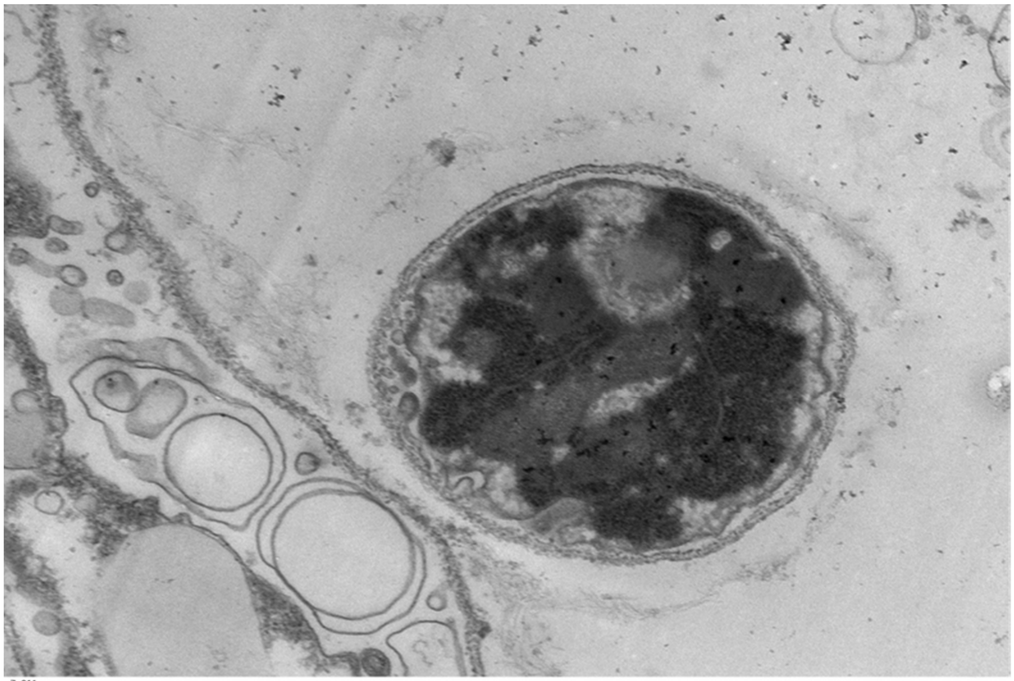

e7_011
Print Mag: 1460x @ 7 mm
15:28:43 3/5/2018

500 nm
HV=80.0kV
Direct Mag: 12000x
AMT Camera System

Camera: XR16, Exposure: 800 (ms) x 1 std. frames, Gain: 1, Bin: 1
Gamma: 1.00, No Sharpening, Normal Contrast

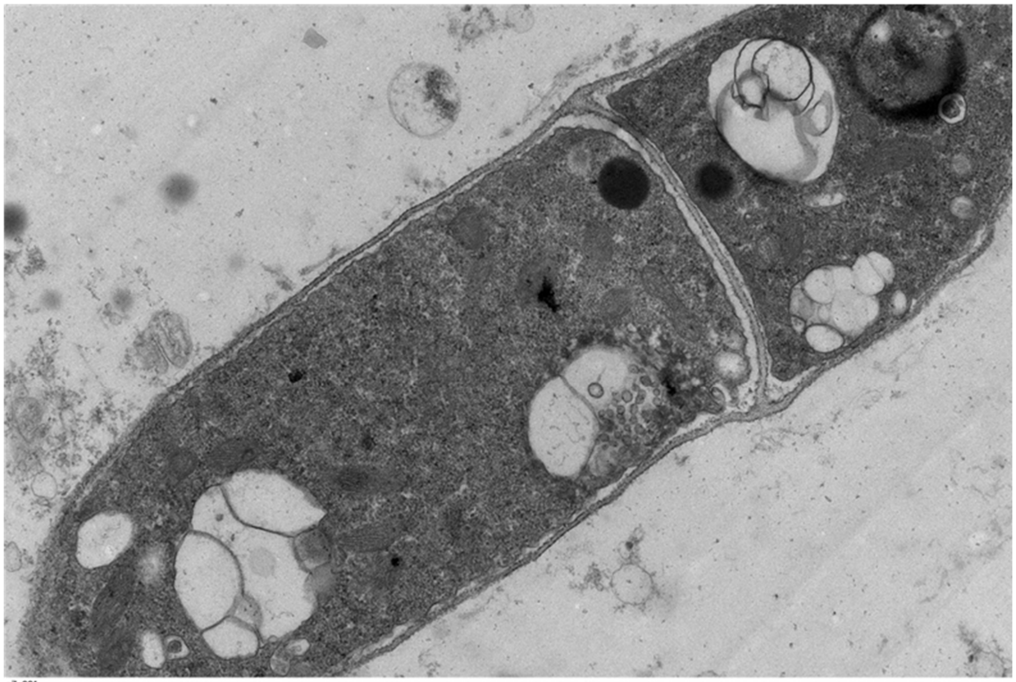

e7_001
Print Mag: 981x @ 7 mm
15:18:54 3/5/2018

800 nm
HV=80.0kV
Direct Mag: 8000x
AMT Camera System

Camera: XR16, Exposure: 800 (ms) x 1 std. frames, Gain: 1, Bin: 1
Gamma: 1.00, No Sharpening, Normal Contrast

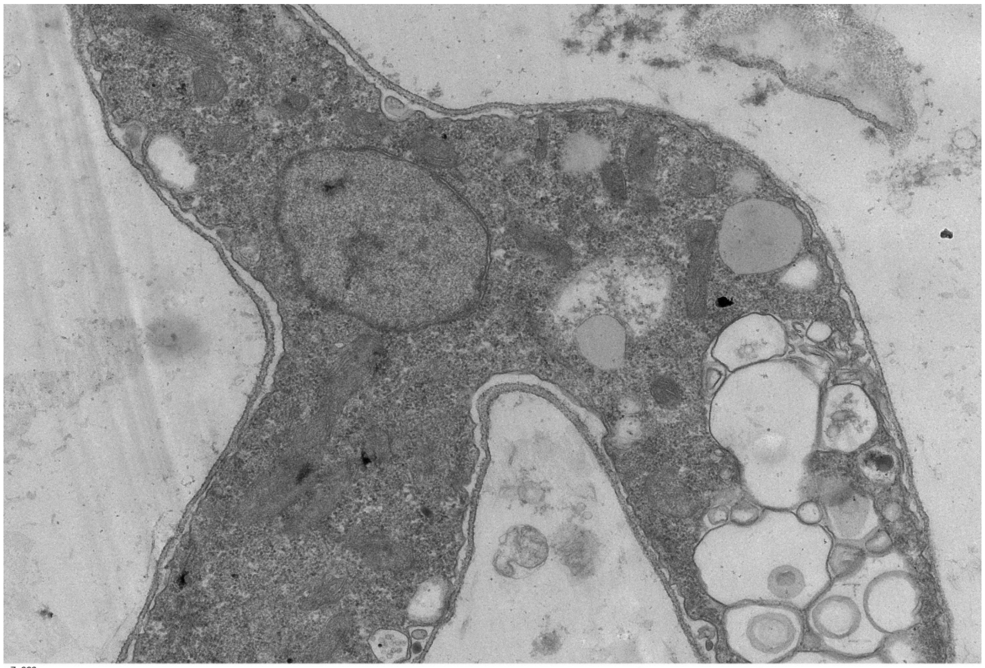

e7_002
Print Mag: 862x @ 7 mm
15:20:00 3/5/2018

1 µm
HV=80.0kV
Direct Mag: 7000x
AMT Camera System

Camera: XR16, Exposure: 800 (ms) x 1 std. frames, Gain: 1, Bin: 1
Gamma: 1.00, No Sharpening, Normal Contrast

$_{Th}$OE*hfb4*

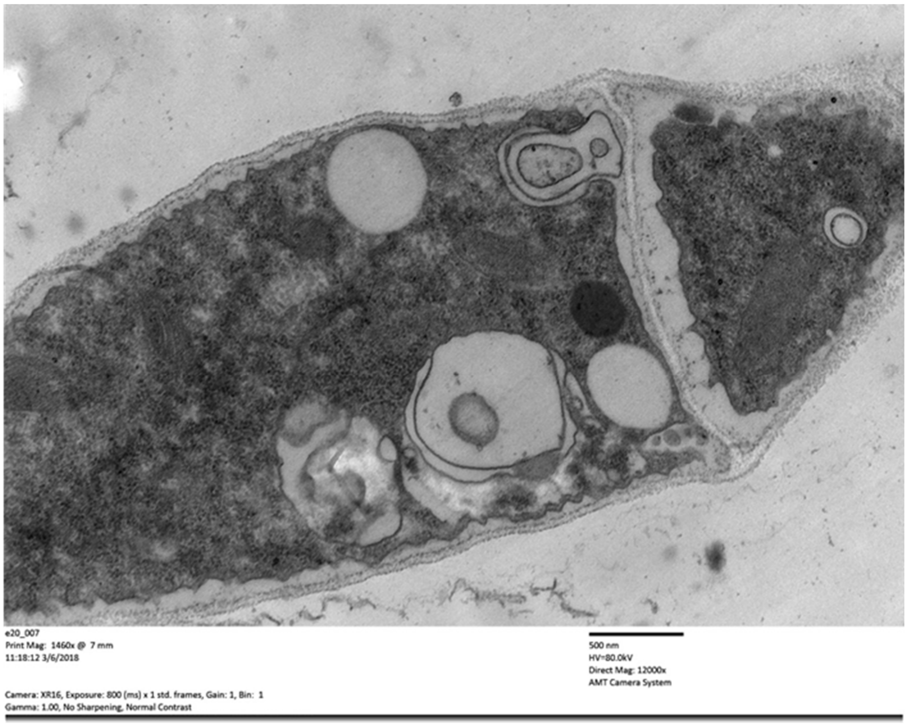
e20_007
Print Mag: 1460x @ 7 mm
11:18:12 3/6/2018
500 nm
HV=80.0kV
Direct Mag: 12000x
AMT Camera System
Camera: XR16, Exposure: 800 (ms) x 1 std. frames, Gain: 1, Bin: 1
Gamma: 1.00, No Sharpening, Normal Contrast

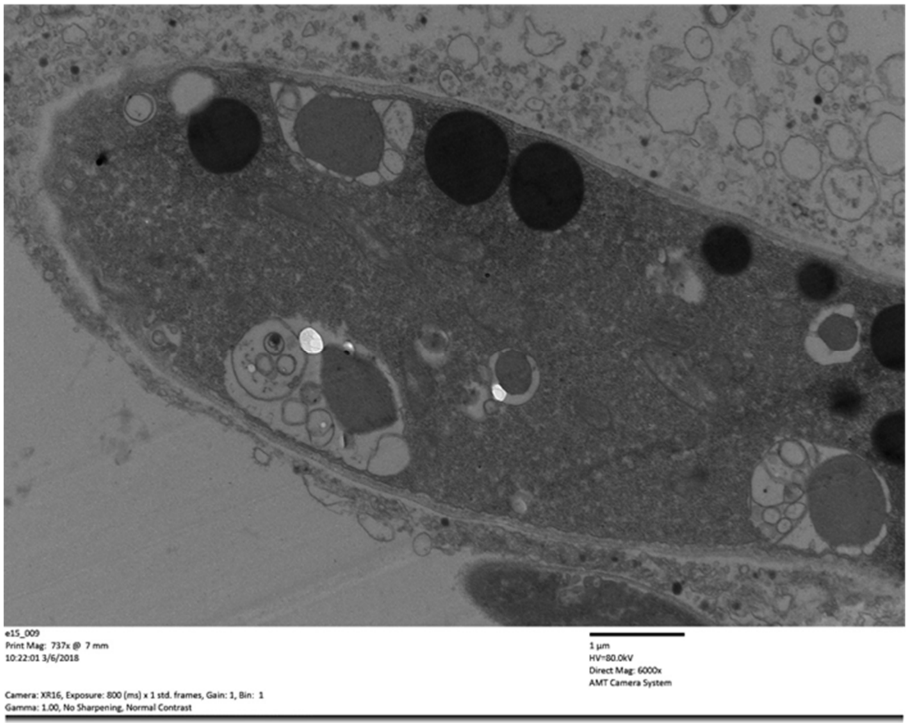
e15_009
Print Mag: 737x @ 7 mm
10:22:01 3/6/2018
1 µm
HV=80.0kV
Direct Mag: 6000x
AMT Camera System
Camera: XR16, Exposure: 800 (ms) x 1 std. frames, Gain: 1, Bin: 1
Gamma: 1.00, No Sharpening, Normal Contrast

$_{Tg}\Delta hfb4$-$_{Tg}\Delta hfb10$

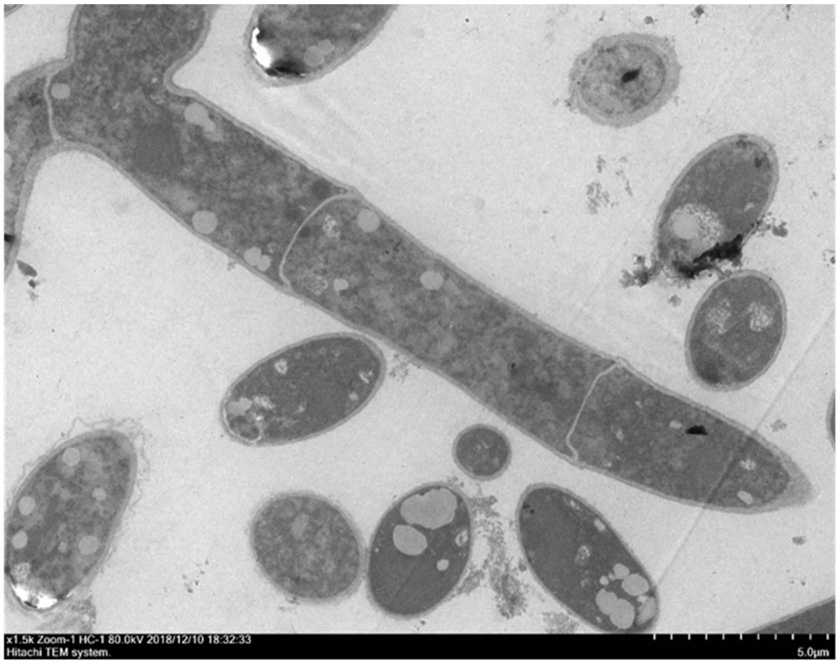

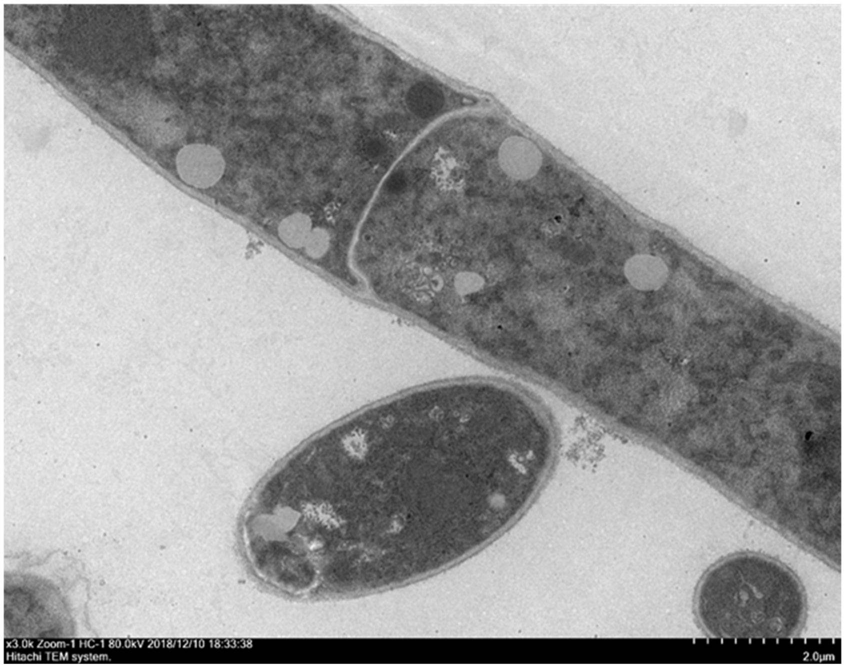

$_{Th}\Delta$*hfb4*-$_{Th}\Delta$*hfb10*

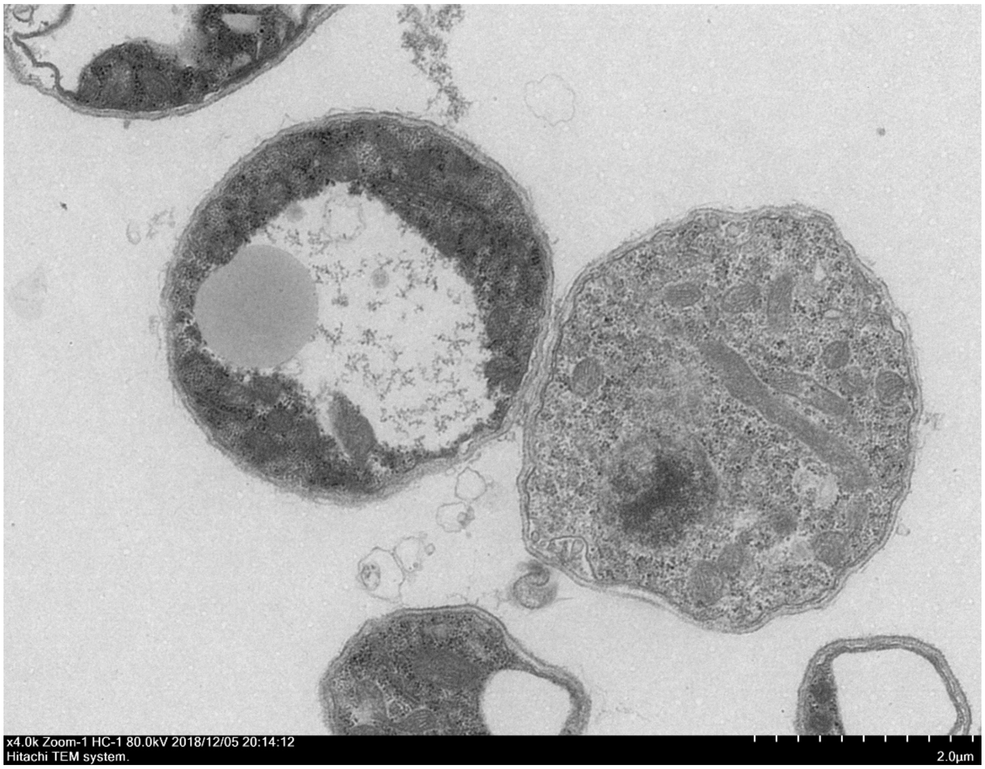

Supplement: S11 Fig — (PDF) [file pgen.1009924.s011.pdf]
