# Supplementary material for: The pleiotropic functions of intracellular hydrophobins in aerial hyphae and fungal spores
Source: PLoS Genet. 2021 Nov 17;17(11):e1009924. doi: 10.1371/journal.pgen.1009924 (PMC8635391; doi:10.1371/journal.pgen.1009924)
Supplement: S12 Fig — (PDF) [file pgen.1009924.s012.pdf]

Supporting Information S12 Fig. Quantification of autophagy-related genes in *Trichoderma* spp. and *P. pastoris* expressing *hfb*s

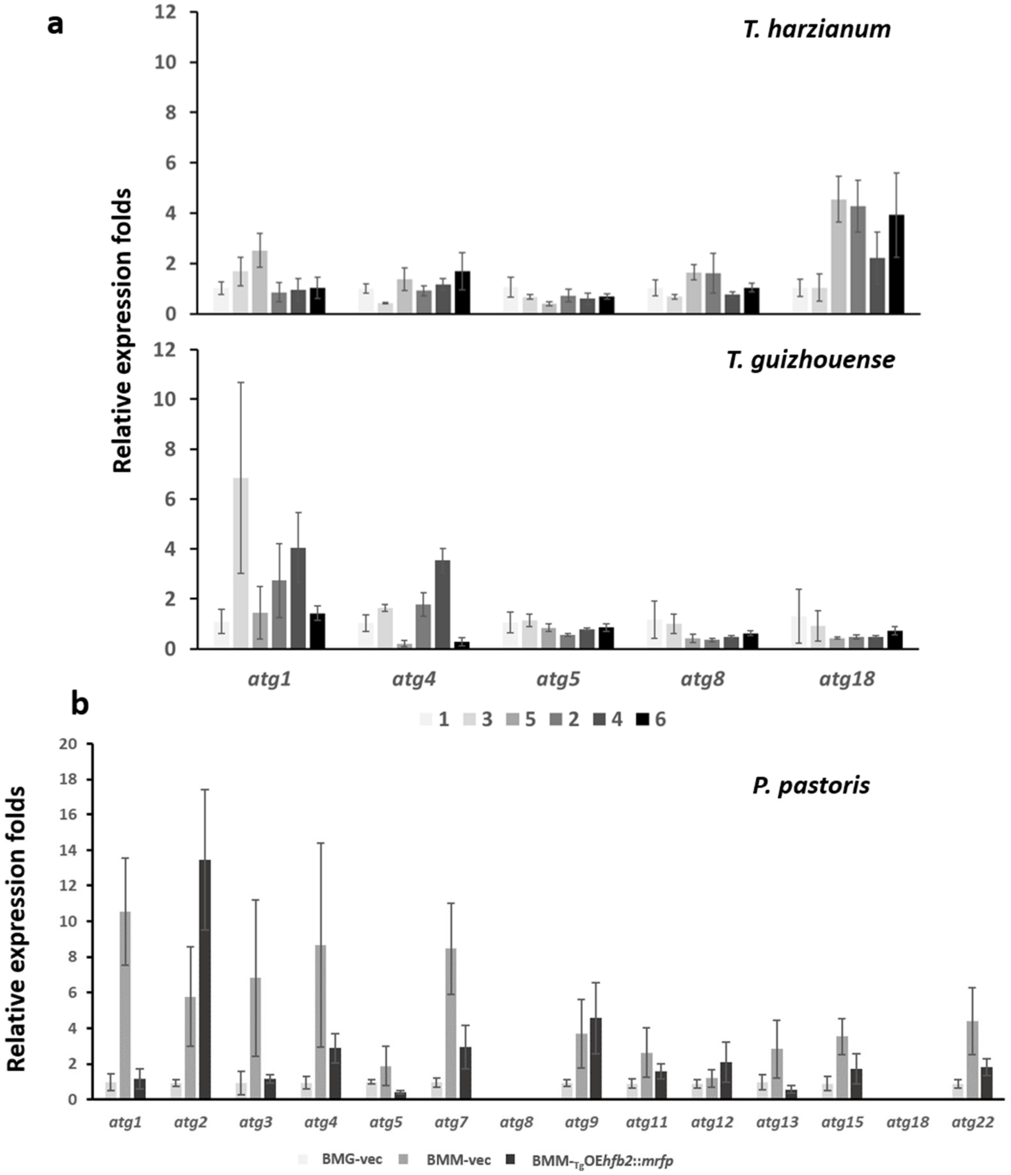

Fig S12 Expression pattern of autophagy-related genes (*atg*) in *Trichoderma* spp. during the formation and maturation of conidiogenic rings and in *Pichia pastoris* strains producing/nonproducing HFBs from *Trichoderma*. The expression ratio of each gene is the mean fold change relative to its expression at the stage of the vegetative growth (see Fig 5) and calibrated by the internal control gene *tef1* for *Trichoderma* spp., and the expression ratio of each gene was compared to its expression in a noninducible sample (BMG-vec) and calibrated by the internal control gene act1 for *P. pastoris* using the $^{2-\Delta\Delta}$Ct method. Error bars represent the standard deviation calculated from four biological replicates. RNAs of *Trichoderma* spp. were extracted from the fungal biomass grown on the desired six positions on the PDA cultures (shown in Fig 5). Position 3 and 4 were the conidiating ring areas. RNA of *P. pastoris* was extracted from

yeast cells grown in inducible (BMM)/noninducible (BMG) cultures. The tested genes were selected based on their reported functions involved in cell autophagy [1] [2, 3]

References

1. Farre JC, Subramani S. Mechanistic insights into selective autophagy pathways: lessons from yeast. Nat Rev Mol Cell Biol. 2016;17(9):537-52. Epub 2016/07/07. doi: 10.1038/nrm.2016.74. PubMed PMID: 27381245; PubMed Central PMCID: PMCPMC5549613.

2. Inoue Y, Klionsky DJ. Regulation of macroautophagy in Saccharomyces cerevisiae. Semin Cell Dev Biol. 2010;21(7):664-70. Epub 2010/04/03. doi: 10.1016/j.semcdb.2010.03.009. PubMed PMID: 20359542; PubMed Central PMCID: PMCPMC2930024.

3. Lipatova Z, Segev N. A Role for Macro-ER-Phagy in ER Quality Control. PLoS Genet. 2015;11(7):e1005390. Epub 2015/07/17. doi: 10.1371/journal.pgen.1005390. PubMed PMID: 26181331; PubMed Central PMCID: PMCPMC4504476.
