# Supplementary material for: The pleiotropic functions of intracellular hydrophobins in aerial hyphae and fungal spores
Source: PLoS Genet. 2021 Nov 17;17(11):e1009924. doi: 10.1371/journal.pgen.1009924 (PMC8635391; doi:10.1371/journal.pgen.1009924)
Supplement: S13 Fig — (PDF) [file pgen.1009924.s013.pdf]

Supporting Information S13 Fig. Herniations on conidiophores of *Trichoderma* spp. from the taxonomic work of W. .M. Jaklitsch [1].

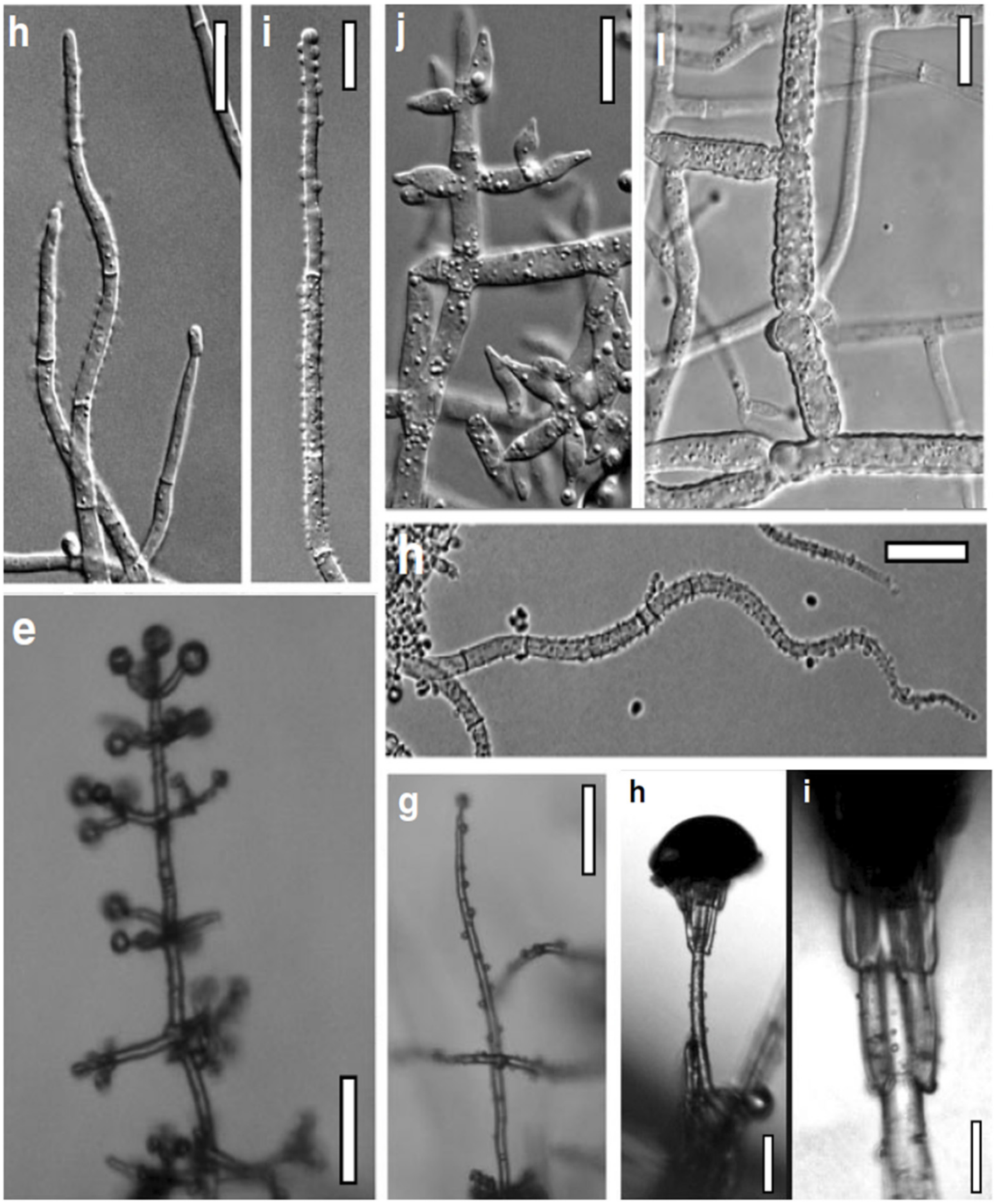

Fig S13 Examples of anamorph morphology showing herniations (protrusions) on conidiophores or aerial hyphae of various *Trichoderma* species as presented in Figs 3, 36, 44, 48, 78, and 97 in the work of Jaklitsch, W.M [1]. Letter labels and species identities are not relevant as taken from the original publication.

References

1. Jaklitsch WM. European species of Hypocrea part II: species with hyaline ascospores. Fungal Divers. 2011;48(1):1-250. Epub 2011/10/14. doi: 10.1007/s13225-011-0088-y. PubMed PMID: 21994484; PubMed Central PMCID: PMCPMC3189789.
