# Supplementary material for: The pleiotropic functions of intracellular hydrophobins in aerial hyphae and fungal spores
Source: PLoS Genet. 2021 Nov 17;17(11):e1009924. doi: 10.1371/journal.pgen.1009924 (PMC8635391; doi:10.1371/journal.pgen.1009924)
Supplement: S14 Fig — (PDF) [file pgen.1009924.s014.pdf]

Supporting Information S14 Fig. Cell wall ultrastructure of HFB mutants of *Trichoderma*

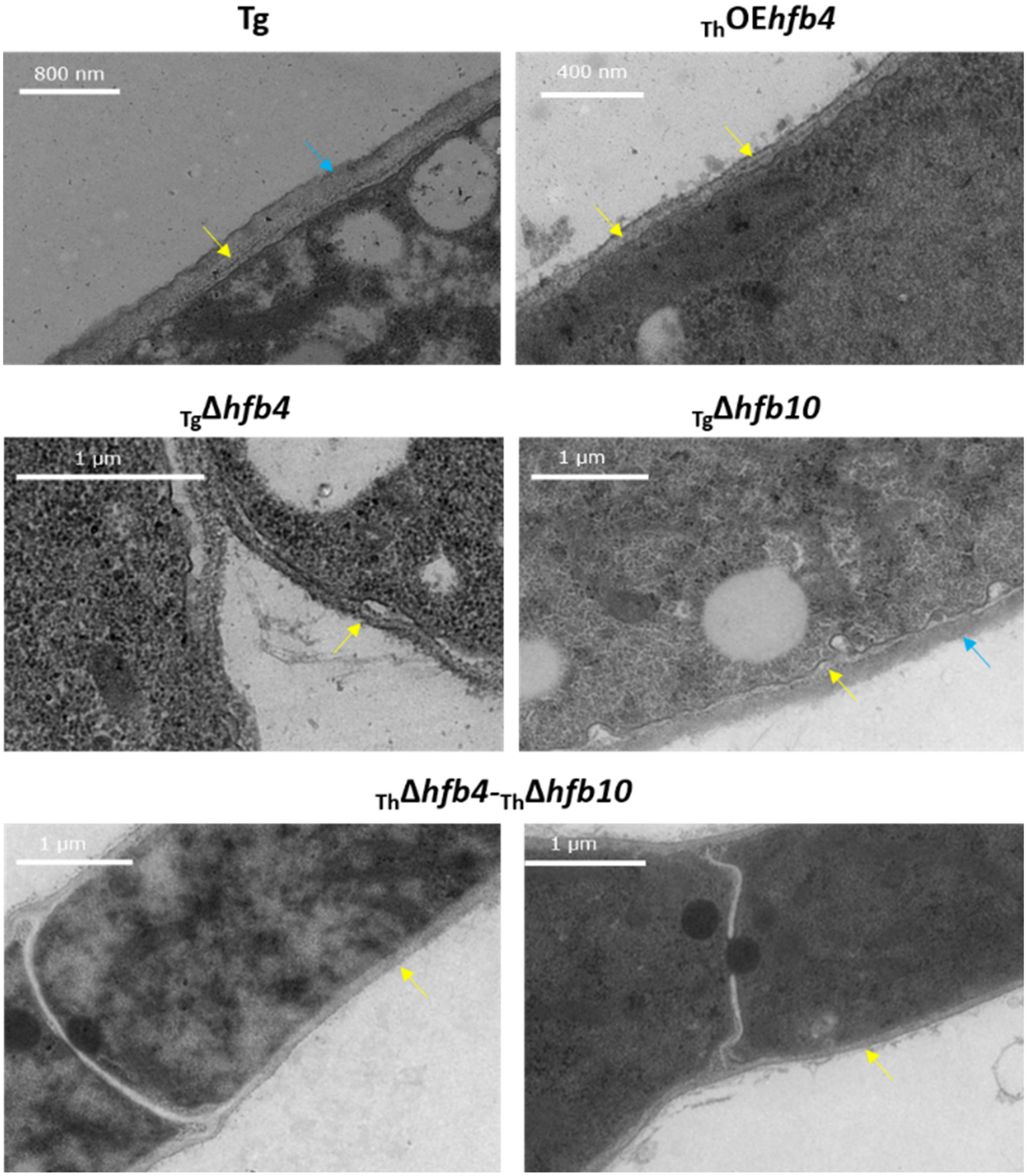

Fig S14 Cell wall ultrastructure of HFB mutants of *Trichoderma*. Strains cultivated on PDA plates at 25 °C in darkness for 48 h. Yellow arrows point to the cell wall, and blue arrows indicate the extracellular matrix.
