# Supplementary material for: The pleiotropic functions of intracellular hydrophobins in aerial hyphae and fungal spores
Source: PLoS Genet. 2021 Nov 17;17(11):e1009924. doi: 10.1371/journal.pgen.1009924 (PMC8635391; doi:10.1371/journal.pgen.1009924)
Supplement: S15 Fig — (PDF) [file pgen.1009924.s015.pdf]

Supporting Information S15 Fig. Response of the wild type, HFB-deficient, and HFB-overexpressing strains of *T. guizhouense* to the conditions of the osmotic stress

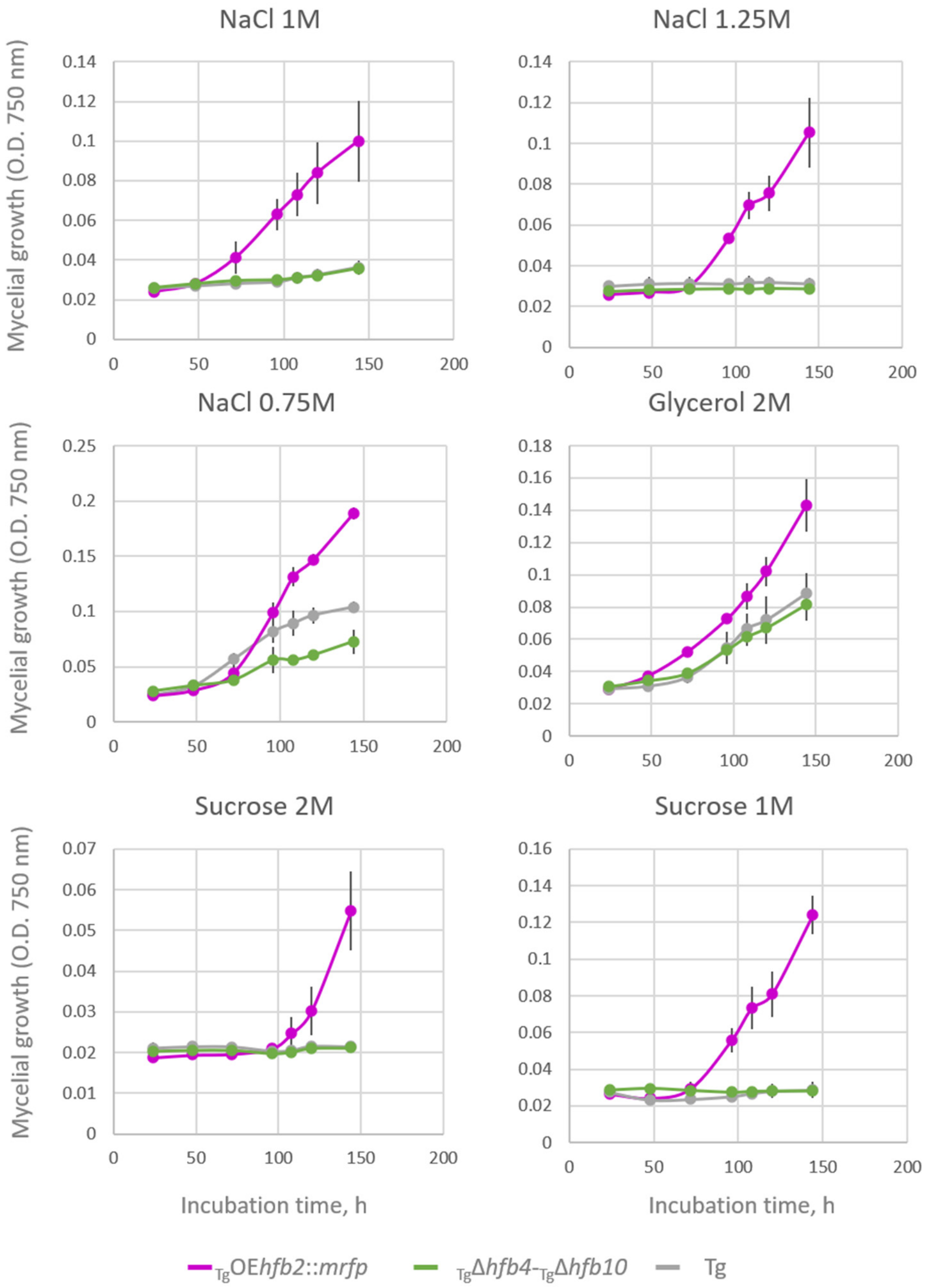

Fig S15 For the testing the resistance to the osmotic stress, strains were inoculated in the glucose synthetic medium (9.89 mM KNO3, 7.35 mM KH2PO4, 6.7 mM KCl, 2.03 mM $MgSO_4 \times 7H_2O$, 0.9 mM $CaCl_2$, 0.094 mM $MnSO_4$ x H_2O, 0.048 mM $ZnSO_4 \times 7H_2O$, 0.18 mM $FeSO_4 \times 7H_2O$, 0.121 mM $CoCl_2 \times 6H_2O$, 1% glucose) supplemented with NaCl (0.75 M, 1 M, or 1.25 M), sucrose (1 M or 2 M) or 2 M glycerol as osmolytes. These experiments were performed in 96-well microplates inoculated with 10^6 spores/ml and incubated at 25°C in darkness. Each assay was repeated four times.
