# Supplementary material for: The pleiotropic functions of intracellular hydrophobins in aerial hyphae and fungal spores
Source: PLoS Genet. 2021 Nov 17;17(11):e1009924. doi: 10.1371/journal.pgen.1009924 (PMC8635391; doi:10.1371/journal.pgen.1009924)
Supplement: S16 Fig — (PDF) [file pgen.1009924.s016.pdf]

Supporting Information S16 Fig. Intracellular, periplasmic, and extracellular vesicles in mature aerial hyphae overproducing HFBs.

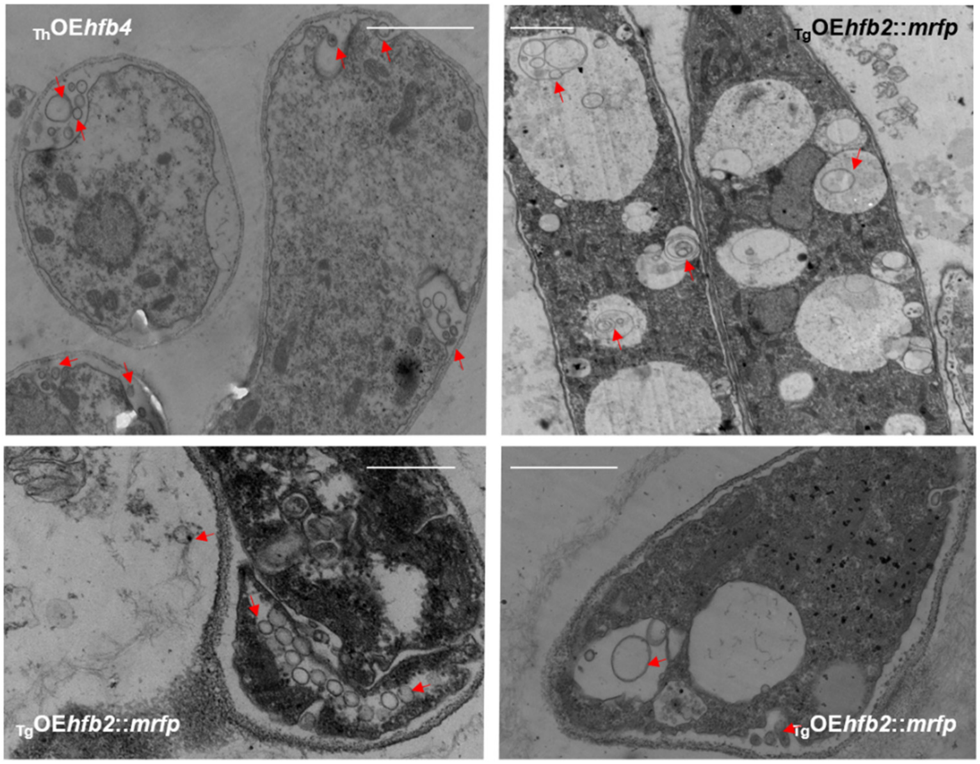

Fig S16 TEM micrographs of *Trichoderma* spp. hyphae overexpressing HFBs without and with fluorescent tags. Red arrows indicate putative HFB-enriched vesicles. Representative images were selected from 523 images obtained for Tg. Samples for TEM were prepared with at least two mutants and 30 images studied per genotype. Mutants are listed in Table 2. Scale bar = 1 µm.
