# Supplementary material for: The pleiotropic functions of intracellular hydrophobins in aerial hyphae and fungal spores
Source: PLoS Genet. 2021 Nov 17;17(11):e1009924. doi: 10.1371/journal.pgen.1009924 (PMC8635391; doi:10.1371/journal.pgen.1009924)
Supplement: S17 Fig — (PDF) [file pgen.1009924.s017.pdf]

Supporting Information S17 Fig. Dynamic release of HFBs during conidiogenesis of *Trichoderma* and an architecture of colonies

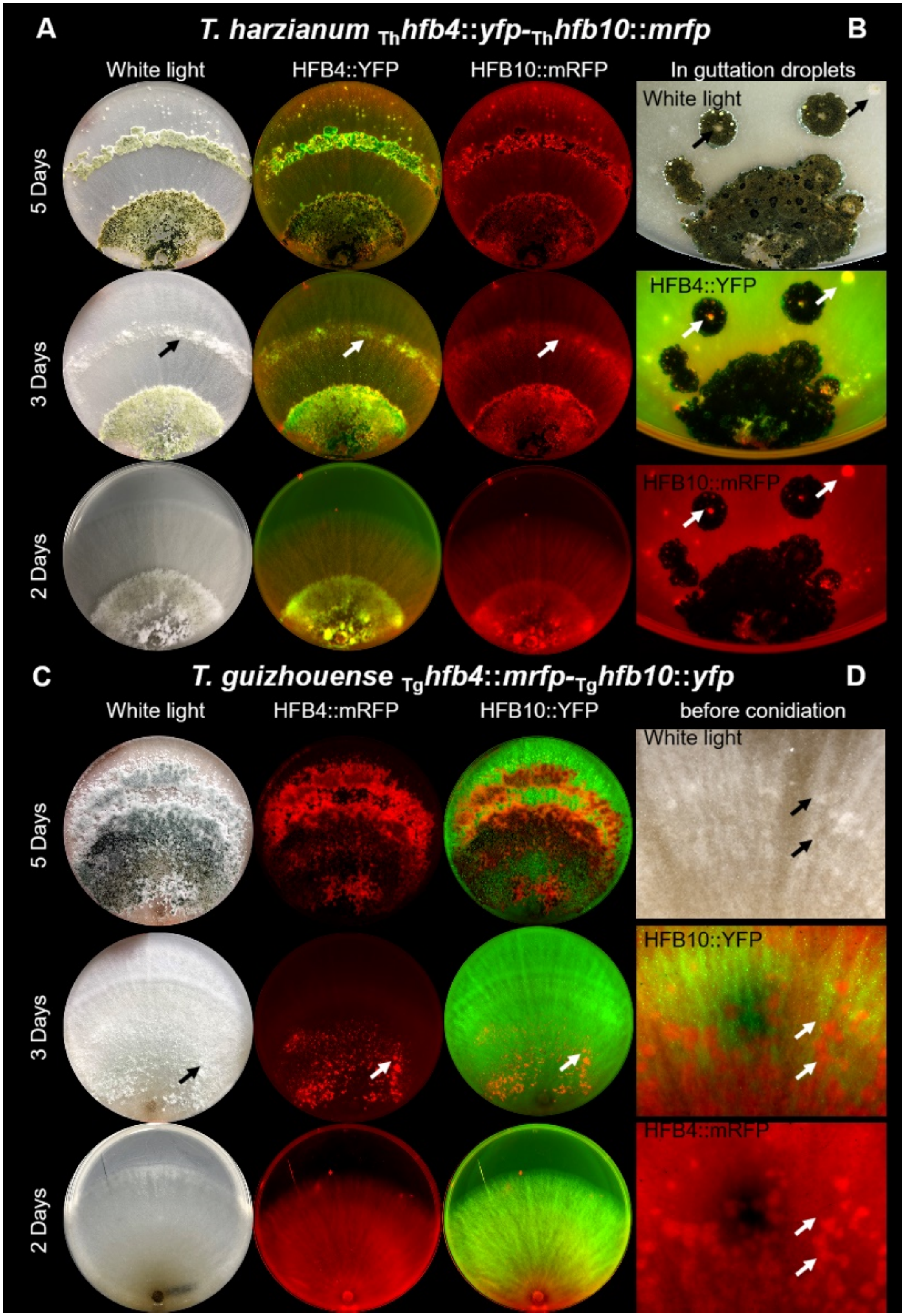

Fig S17 The dynamic release of HFBs during conidiogenesis of *Trichoderma*.

Note: HFB4 is labeled with red and yellow fluorescent proteins in Tg and Th, respectively, while HFB10 is labeled with yellow and red fluorescent proteins in Tg and Th, respectively.

A, Time course-dependent production of HFB4 and HFB10 during the development of Tg colonies imaged by a ChemiDoc MP system without magnification, plate diameter 9 cm. Arrows point to primordial pustules with premature conidiophores. *T. guizhouense* is autofluorescent in the green spectrum.

B, Close-up images obtained from old cultures showing accumulation of HFBs in guttation droplets above conidia (arrows) but not in drops above young hyphae. C, Time course-dependent production of HFB4 and HFB10 during the development of Th colonies imaged by a ChemiDoc MP system without magnification, plate diameter 9 cm. Arrows point to primordial pustules with premature conidiophores. Note: *T. harzianum* is strongly autofluorescent in the green spectrum. D. Production of HFB4 and HFB10 at the spots of subsequent formation of conidiophores and spores. Primordial pustules are shown by arrows.
