# Supplementary material for: The pleiotropic functions of intracellular hydrophobins in aerial hyphae and fungal spores
Source: PLoS Genet. 2021 Nov 17;17(11):e1009924. doi: 10.1371/journal.pgen.1009924 (PMC8635391; doi:10.1371/journal.pgen.1009924)
Supplement: S18 Fig — (PDF) [file pgen.1009924.s018.pdf]

Supporting Information S18 Fig. Colony architecture and dynamic HFB accumulation in aerial hyphae before and during conidiogenesis

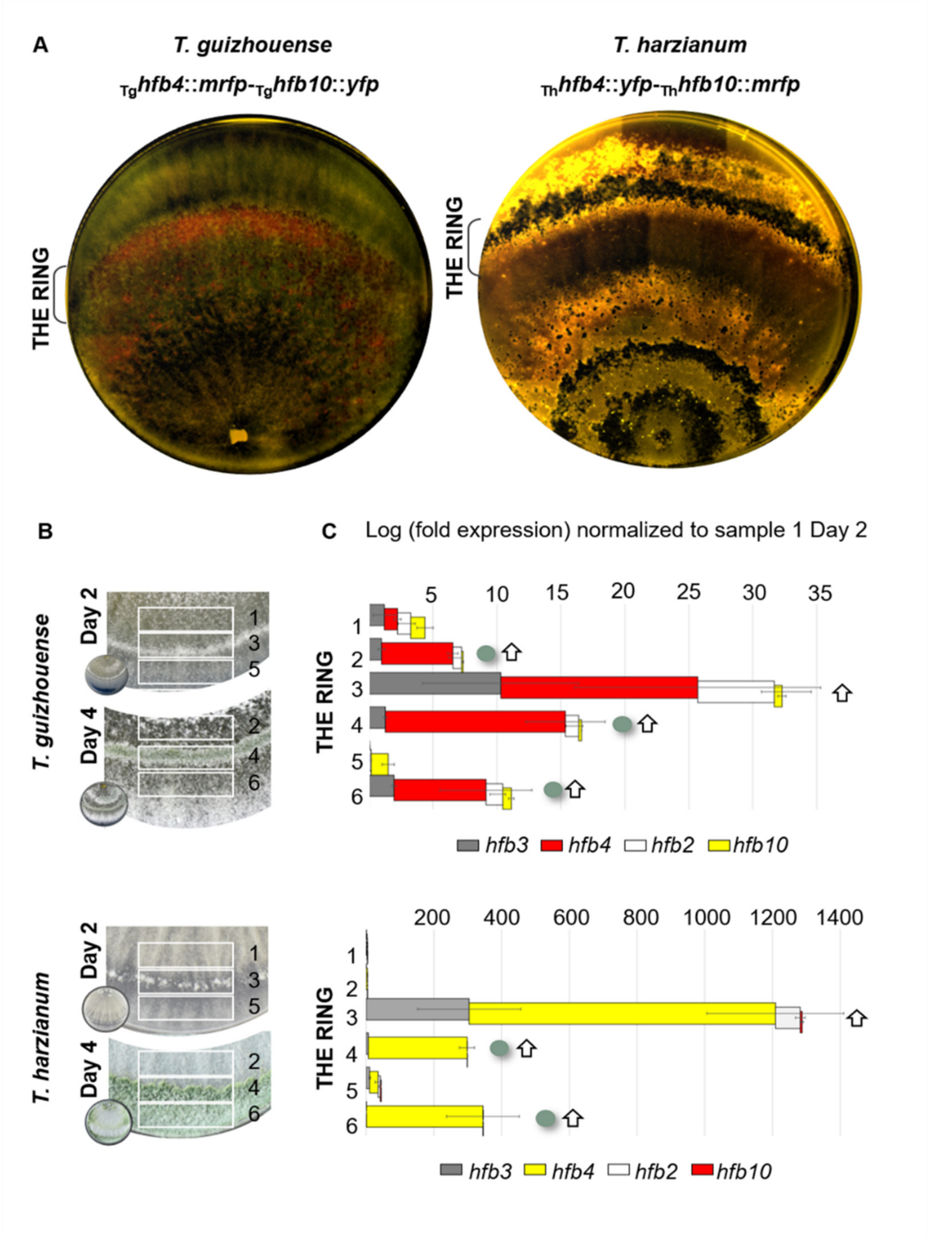

Fig S18 (A) Fluorescent imaging of conidiating *Trichoderma* colonies labeled with HFB4 and HFB10. The time course images based on a similar principle are shown in Supporting Information S18. Note that HFB4 is

labeled with red and yellow fluorescent proteins in Tg and Th, respectively, while HFB10 is labeled with yellow and red fluorescent proteins in Tg and Th, respectively. (B) Formation of the conidiating ring during the fine time course of conidiogenesis of *Trichoderma* spp. Photos show sampled areas of *Trichoderma* cultures for gene expression analysis (boxed). (C) Relative expression of *hfb*s during the fine time course of conidiogenesis of *Trichoderma* spp., quantified by qPCR. The values are normalized to those of the housekeeping gene *tef1* and expressed in relation to aerial hyphae prior to conidiogenesis (position 1 in B). Horizontal bars indicate standard deviations. Green dots and white arrows indicate spores and conidiophores appearing in stages, respectively.
