# Supplementary material for: The pleiotropic functions of intracellular hydrophobins in aerial hyphae and fungal spores
Source: PLoS Genet. 2021 Nov 17;17(11):e1009924. doi: 10.1371/journal.pgen.1009924 (PMC8635391; doi:10.1371/journal.pgen.1009924)
Supplement: S19 Fig — (PDF) [file pgen.1009924.s019.pdf]

Supporting Information S19 Fig. Secondary growth of $_{Tg}\Delta$*hfb4*

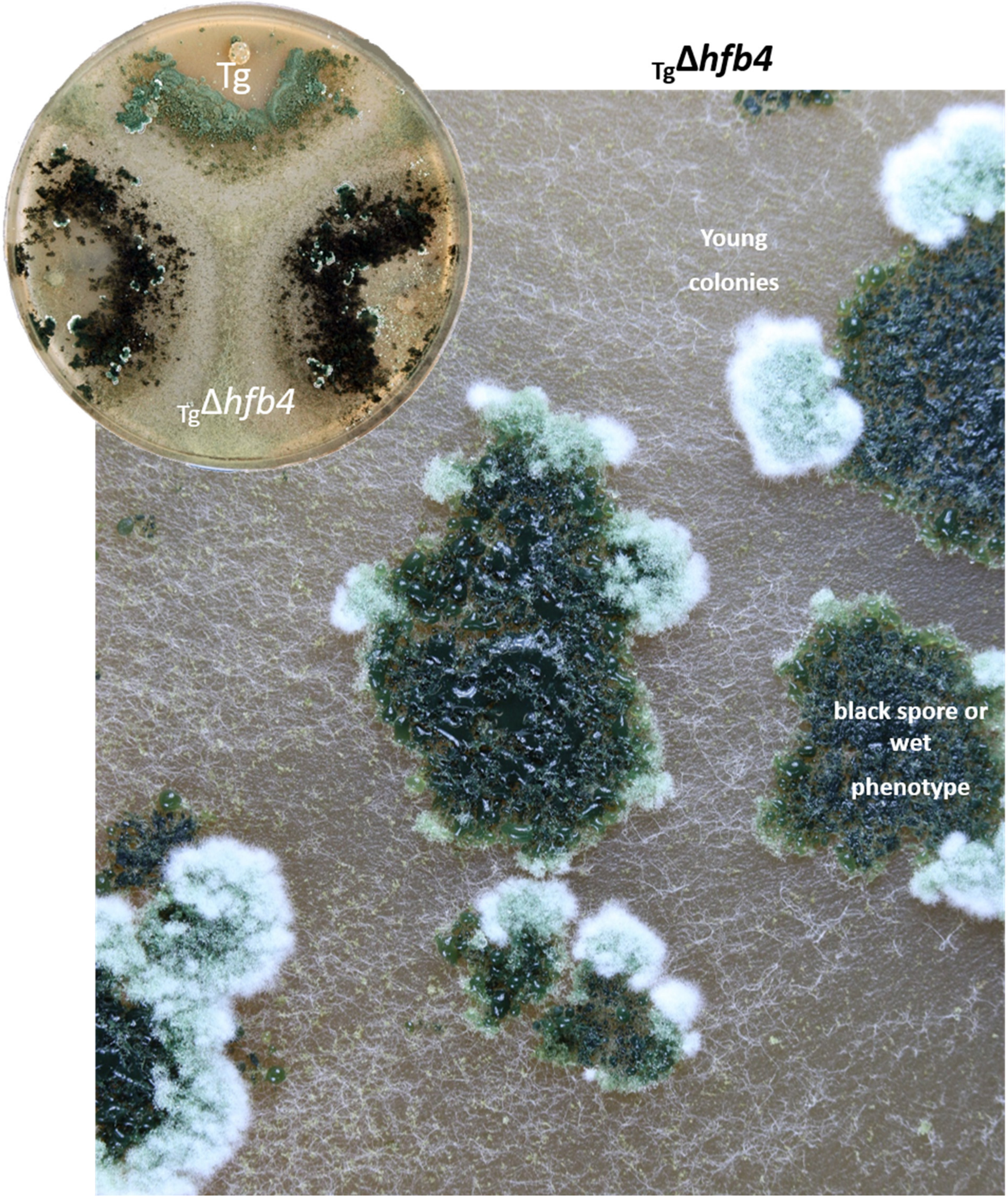

Fig S19 "Black spore" or wetted phenotype of $_{Tg}\Delta$*hfb4* mutants after two weeks of cultivation. Note the emergence of young colonies above autolyzed hyphae due to the altered dormancy control in this mutant.
