# Supplementary material for: The pleiotropic functions of intracellular hydrophobins in aerial hyphae and fungal spores
Source: PLoS Genet. 2021 Nov 17;17(11):e1009924. doi: 10.1371/journal.pgen.1009924 (PMC8635391; doi:10.1371/journal.pgen.1009924)
Supplement: S20 Fig — (PDF) [file pgen.1009924.s020.pdf]

Supporting Information S20 Fig. Interaction monitoring between HFBs using quartz crystal microbalance (QCM)

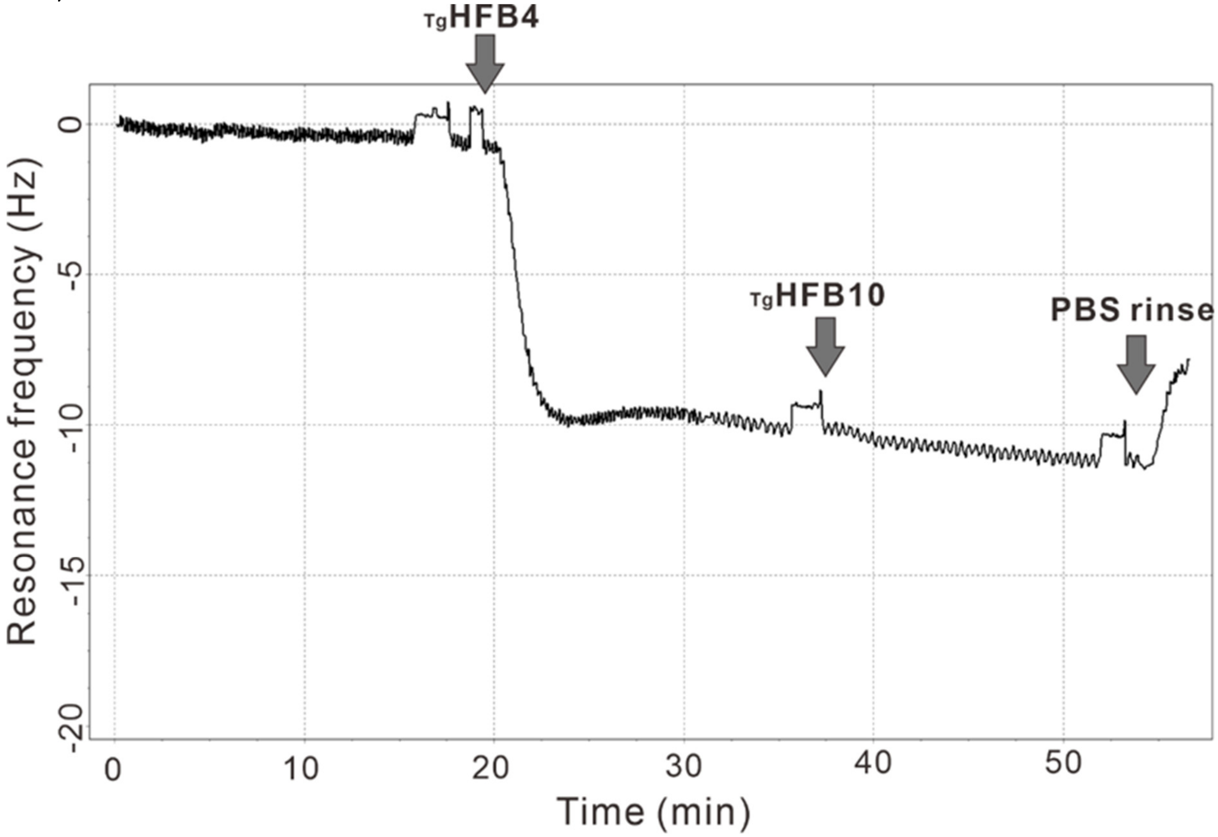

Fig S20 Representative adsorption kinetics recorded using quartz crystal microbalance (QCM) for the interaction of $_{Tg}$HFB4 and $_{Tg}$HFB10 on the surface of borosilicate substrates. Frequency changes (Δf) are shown for the 3rd overtone normalized to the fundamental resonance frequency. Arrows represent the sample application and start of the buffer rinse by 100 mM PBS, respectively. All measurements were conducted at 25℃ with a Q-Sense E4 system (Biolin Scientific, Sweden) for at least three times and quartz crystal sensors (4.95 MHz, AT-cut, gold electrode, LOT Quantum Design, Germany) coated with a homogeneous borosilicate film was adopted. *Pichia pastoris*-produced HFB proteins were purified and then buffer-exchanged into a 100mM PBS buffer at a concentration of 5 µM protein. $_{Tg}$HFB4 was applied first until the surface was saturated, which was followed by a $_{Tg}$HFB10 application and a buffer rinse. The frequency shift of the surface corresponds to a coating mass of 200.75±30.90 ng cm-2 for HFB4 and a coating mass of 208.78±16.32 ng cm^{-2} after HFB10 application, indicating a non-interaction between the tested HFBs. The Sauerbrey formula m= -k·Δf, where k≈ 18 ng/cm^{-2}·Hz [1].

References

1. Przylucka A, Akcapinar GB, Bonazza K, Mello-de-Sousa TM, Mach-Aigner AR, Lobanov V, et al. Comparative Physiochemical Analysis of Hydrophobins Produced in Escherichia Coli And Pichia Pastoris. Colloids Surf B Biointerfaces. 2017;159:913-23. Epub 2017/09/15. doi: 10.1016/j.colsurfb.2017.08.058. PubMed PMID: 28903187.
