# Supplementary figures and images for: The pleiotropic functions of intracellular hydrophobins in aerial hyphae and fungal spores
Source: PLoS Genet. 2021 Nov 17;17(11):e1009924. doi: 10.1371/journal.pgen.1009924 (PMC8635391; doi:10.1371/journal.pgen.1009924)

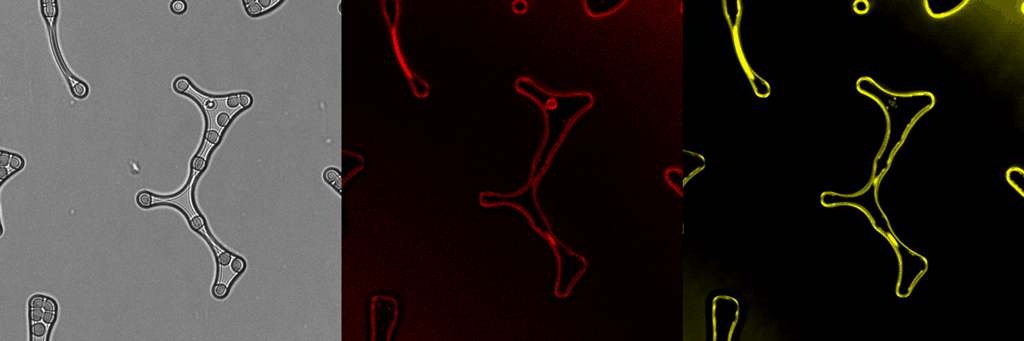

Supplement: S5 Video — (GIF) [file pgen.1009924.s025.gif]
