# Supplementary material for: The pleiotropic functions of intracellular hydrophobins in aerial hyphae and fungal spores
Source: PLoS Genet. 2021 Nov 17;17(11):e1009924. doi: 10.1371/journal.pgen.1009924 (PMC8635391; doi:10.1371/journal.pgen.1009924)
Supplement: S1 File — (PDF) [file pgen.1009924.s027.pdf]

Supporting Information S1 File Detailed Methods

# Table of Contents

Supporting Information S1 File Detailed Methods ........ 1
Gene deletion ........ 1
*In situ* fluorescence labeling ........ 2
Overexpression of *hfb* encoding genes in *Trichoderma* ........ 4
Reverse complement of *hfb*-encoding genes in *hfb* deletion mutants ........ 5
Heterologous expression of *hfb*-encoding genes from *Trichoderma* in *P. pastoris* ........ 6
References ........ 7

## Gene deletion

HFB-encoding genes in the genomes of *T. harzianum* CBS 226.95 (GenBank: MBGI00000000.1) and *T. guizhouense* NJAU 4742 (GenBank: LVVK00000000.1) were obtained by genome mining as described in Cai et al. [1], and gene IDs are listed in Table 1. Vectors for gene deletion were constructed as described by Cai et al. [1]. Briefly, the gene of interest was replaced by the *hph* cassette or by the neocassette by transformation. Positive mutants were purified by the method of single spore isolation and confirmed for the absence of the target gene with the primer pair F3 and R3. All vectors and PCR products were confirmed by sequencing. After purification and verification by PCR (shown in Fig A with two randomly selected mutants).

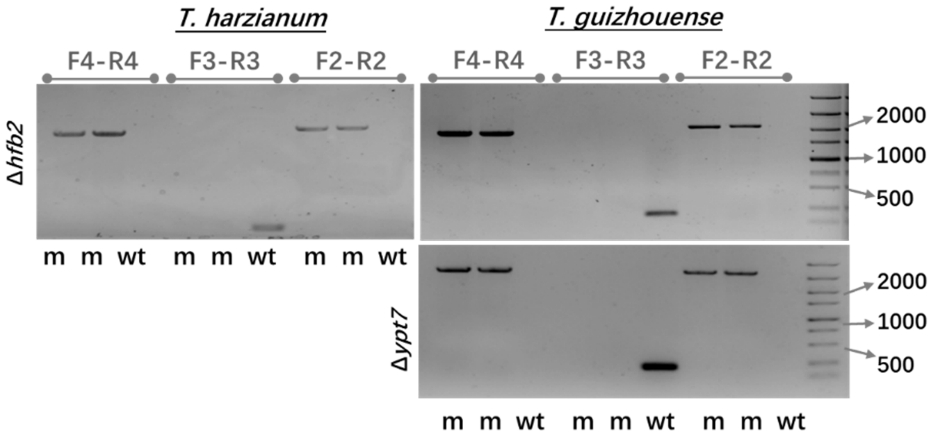


*Fig A PCR verification of gene deletion mutants. wt represents the corresponding wild-type strains; m represents mutants. DL5000 DNA marker (Vazyme, China) was used in gel electrophoresis*

*In situ* fluorescence labeling

A schematic diagram for vector construction of fluorescent labeling is shown in Fig B. Fragments were amplified by PCR and fused into PUC19 together with the selected fluorescent protein gene and the selection marker cassette. A flexible linker sequence (GGGGS×3) was designed to connect the HFB protein and the fluorescent protein. A 6×His tag was fused to the C-terminus of the fluorescent protein, which allows immunological detection of the fused protein. Specifically, HFB4 in the *T. guizhouense* NJAU 4742 strain was labeled by mRFP using *hph* as the marker, and then HFB10 was labeled by YFP using *neo* as the marker. In *T. harzianum* CBS 226.95, HFB4 was labeled by YFP with *hph,* and HFB10 was labeled with mRFP and *neo* to rule out the position effect of the labeling sequence on protein localization. The *hph* cassette, neocassette and mrfp gene were cloned from plasmids of pPcdna1-hph, pKi-Gen and pPICZα-mRFP that were maintained in the Microbiology and Comparative Genomics group of TU Wien (Austria). The *yfp* gene was cloned from the plasmid of pDS22 that was kindly provided by Dr. Norio Takeshita and Dr. Reinhard Fischer (Institute for Applied Biosciences, Karlsruhe Institute of Technology, Germany). After purification, one double-labeled mutant was generated for each species. The results of *hfb* labeling are shown in Fig C.

Additionally, a mutant expressing mRFP after the signal peptide of *hfb4* under the control of the native promoter of *hfb4* ($P_{hfb4}$) was generated (Fig D). A 1.2 kb fragment containing the *hfb4* promoter in frame with the HFB4 native signal peptide sequence (i.e., ATGAAGTTCTCTGCCATCGCTCTCTTCGCCTCGCTGGCCATTGCCGCGCCCGCCACGGAGGCC) and a 1.2 kb fragment containing the native transcription termination of *hfb4* were cloned from *T. guizhouense* NJAU 4742 and fused into the PUC19 plasmid together with the mRFP gene and the selection marker cassette. The vector was constructed to allow the expression of mRFP under the control of $P_{hfb4}$ and its secretion led by the secretion signal of HFB4. One transformant was screened out harboring the correct construct (shown in Fig E).

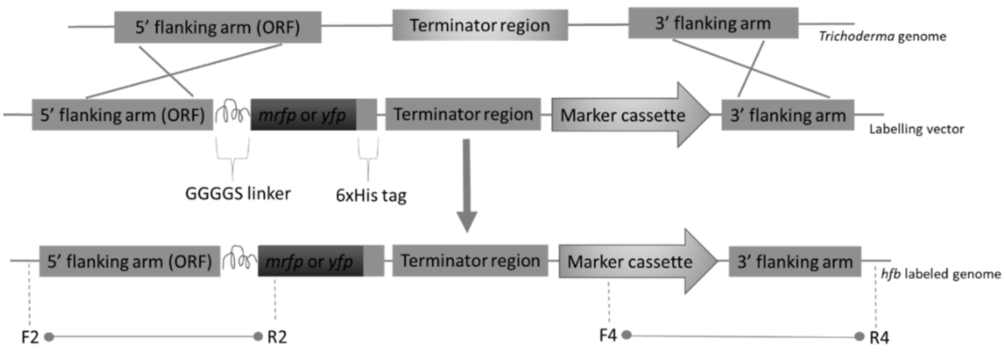


***Fig B** Schematic diagram of* hfb *labeling via homologous recombination and mutant screening*

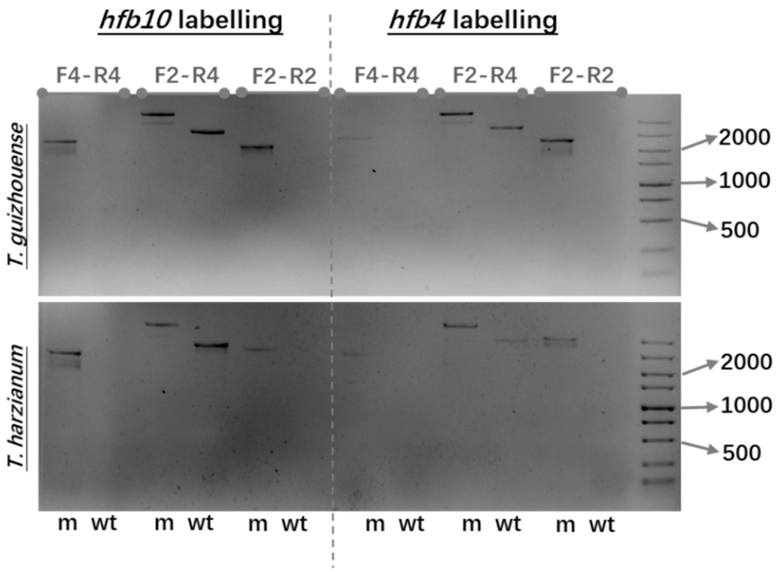


*Fig C* PCR verification of the double-labeled mutants. wt represents the corresponding wild-type strains; m represents mutants. A DL5000 DNA marker (Vazyme, China) was used in gel electrophoresis.

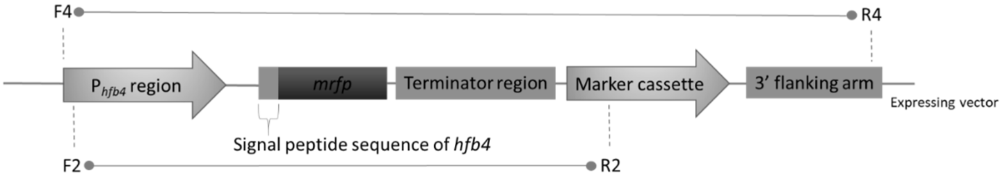


*Fig D* Schematic diagram of mRFP expression under the native promoter of hfb4 (*P*$_{hfb4}$) from T. guizhouense NJAU 4742 and mutant screening.

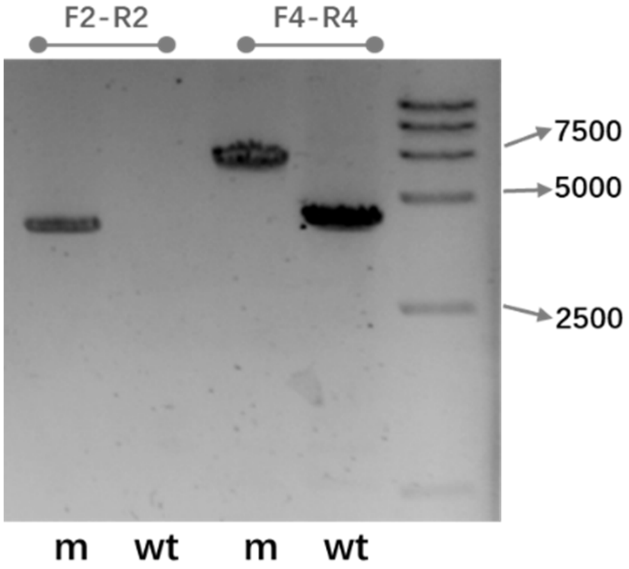


*Fig E* PCR verification of mutant expressing mRFP. wt represents the wild-type strain of T. guizhouense NJAU 4742; m represents mutants. A DL15000 DNA marker (Vazyme, China) was used in gel electrophoresis.

Overexpression of *hfb* encoding genes in *Trichoderma*

Overexpressing vectors were constructed as shown in Fig F. The open reading frame (ORF) of the *hfb* gene and its native terminator region was cloned from *Trichoderma* genomic DNA and inserted into the pUCPcdna1-hph plasmid (Cla I predigested) after a constitutive promoter $P_{cdna1}$ from *T. reesei* QM6a [2]. After purification and verification by PCR (shown in Fig G with two randomly selected mutants), five $_{Th}$OE*hfb4* and two $_{Th}$OE*hfb10 mutants* were obtained for *T. harzianum* CBS 226.95, and three $_{Tg}$OE*hfb4* and two $_{Tg}$OE*hfb10* mutants were obtained for *T. guizhouense* NJAU 4742.

To overexpress *hfb2* with a fluorescent tag (*mrfp*) in *T. guizhouense* NJAU 4742 under the constitutive promoter $P_{cdna1}$, a 0.5 kb fragment of *hfb2* containing the ORF and a 1.3 kb terminator region from the genomic DNA of *T. guizhouense* NJAU 4742 were obtained by PCR. The PCR products were purified and fused together with the *mrfp* gene into the ClaI-digested pPcdna1-hph plasmid in the order shown in Fig H. A GGGGS ×3 linker and a His ×6 tag were introduced into the construction during primer synthesis as mentioned above. The transformation resulted in two positive mutants confirmed by PCR, as shown in Fig I.

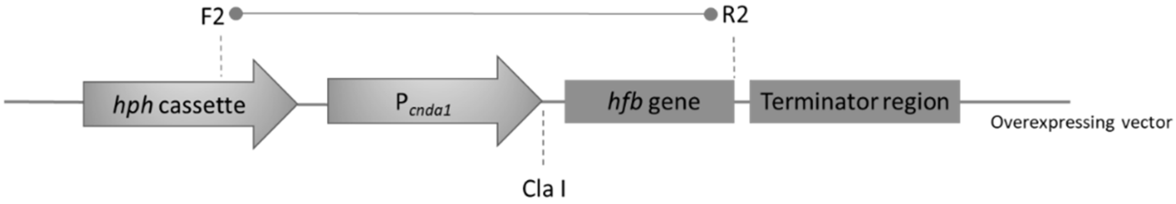


*Fig F Schematic diagram of overexpressing* hfbs *under constitutive promoter* $P_{cnda1}$ *and mutant screening.*

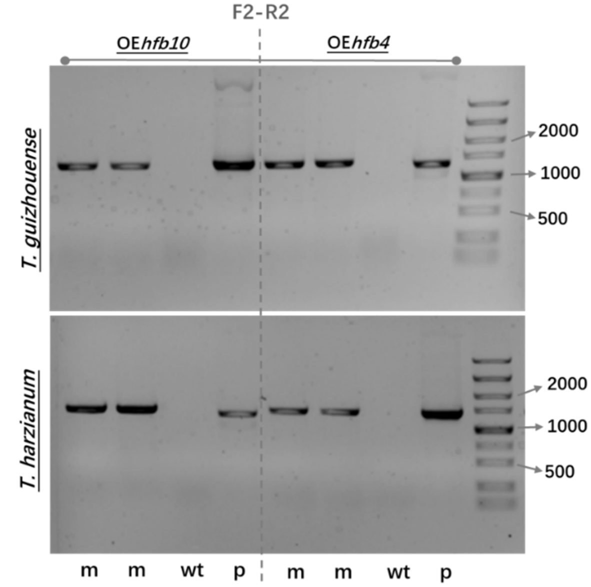


*Fig G PCR verification of mutants overexpressing* hfb4 *or* hfb10*. wt represents the corresponding wild-type strains; m represents mutants; p, positive control cloned from the corresponding plasmid. A DL5000 DNA marker (Vazyme, China) was used in gel electrophoresis.*

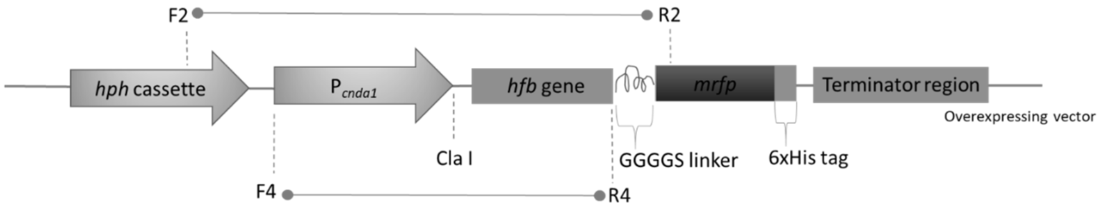


***Fig H*** *Schematic diagram of overexpressing* mrfp-*fused* hfbs *under constitutive promoter P*$_{\mathrm{cnda1}}$ *and mutant screening.*

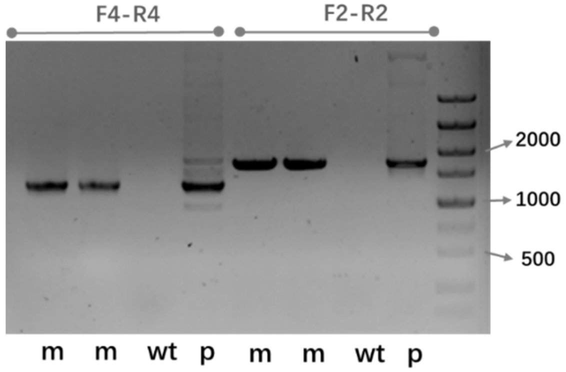


***Fig I*** *PCR verification of mutant overexpressing* mrfp-*fused* hfb2 *in* T. guizhouense *NJAU 4742. wt represents the corresponding wild-type strains; m represents mutants; p, positive control cloned from the corresponding plasmid. A DL5000 DNA marker (Vazyme, China) was used in gel electrophoresis.*

<u>Reverse complement of *hfb*-encoding genes in *hfb* deletion mutants</u>

Reverse complement vectors were constructed as shown in Fig J with two strategies, namely, with or without the fluorescent tag. The open reading frame (ORF) of the *hfb* gene and its native promoter and terminator region was amplified from *Trichoderma* genomic DNA and fused with the neocassette. After purification and verification, PCR confirmation of each genotype is shown in Fig K with two randomly selected mutants.

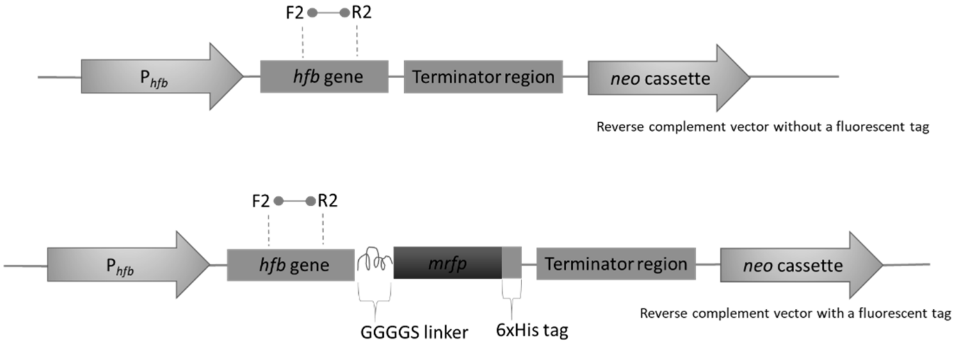


***Fig J*** *Schematic diagram of the reverse complement of* hfbs *to the respective* hfb-*deletion mutant.*

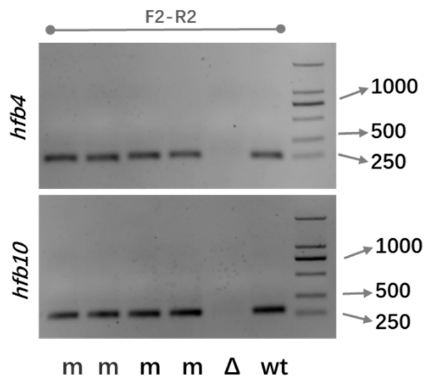


*Fig K Transcriptional verification (RT-PCR) of mutant reverse complemented with* hfb4 *or* hfb10 *with or without a fluorescent tag. wt represents the corresponding wild-type strains; m represents mutants (red font highlights the fluorescently labeled strains); Δ represents the* hfb-*deletion mutant. A DL5000 DNA marker (Vazyme, China) was used in gel electrophoresis. RNA extraction was carried out for 48-h-old PDA cultures of each genotype, and cDNA synthesis was performed as described in the Materials and Methods.*

<u>Heterologous expression of *hfb*-encoding genes from *Trichoderma* in *P. pastoris*</u>

The EasySelect$^{TM}$ *Pichia* Expression Kit was used to express genes from *Trichoderma* in *P. pastoris* strain KM71H, according to the manufacturer's instructions (Invitrogen, USA). The amplified *hfb* gene (without signal peptide or intron sequences) was inserted into the position between the restriction site of EcoR I and Xba I of plasmid pPICZαA (Fig L). To express recombinant proteins with a fluorescent tag, GFPuv or mRFP was adopted and fused at the C-terminus of *hfb* (Fig M). In addition, the native *Saccharomyces cerevisiae* α-factor secretion signal was synthesized at the N-terminus of the *hfb* gene and a His ×6 epitope at the C-terminus. Zeocin resistance driven by the *sh ble* cassette was used for selection. Electroporation resulted in five $_{Tg}$OE*hfb4 mutants*, four $_{Tg}$OE*hfb10 mutants*, ten $_{Tg}$OE*hfb2 mutants*, five $_{Tg}$OE*hfb4-gfpuv* mutants and six $_{Tg}$OE*hfb2-mrfp* mutants (shown in Fig N with one randomly selected mutant).

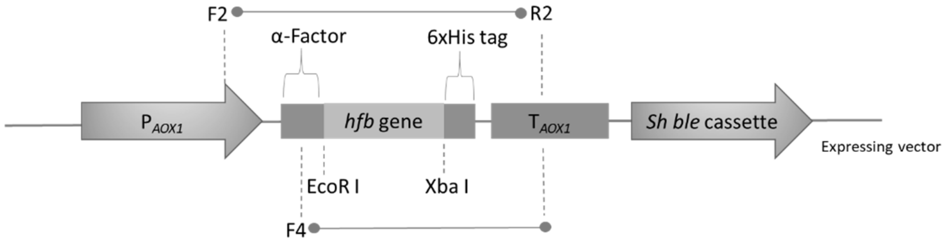


*Fig L Schematic diagram of expressing* hfbs *under the methanol-inducible promoter $P_{AOX1}$ in* P. pastoris *and mutant screening. α-Factor, native* S. cerevisiae *α-factor secretion signal; $T_{AOX1}$, native transcription termination from* AOX1 *gene of* P. pastoris*; Sh ble cassette, from* Streptoalloteichus hindustanus ble *gene driving resistance to Zeocin.*

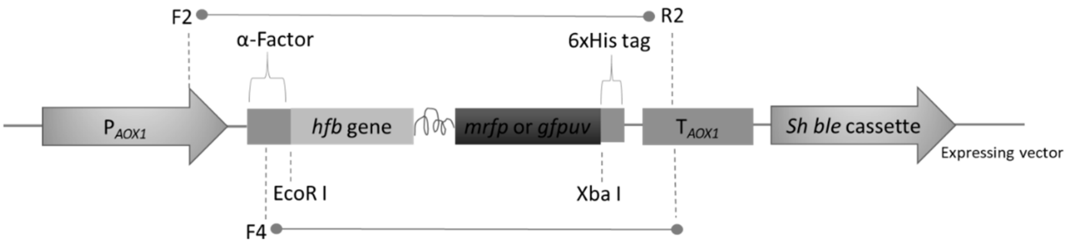


*Fig M Schematic diagram of expressing fluorescently tagged* hfb*s under the methanol-inducible promoter* $P_{AOX1}$ *in* P. pastoris *and mutant screening. α-Factor, native* S. cerevisiae *α-factor secretion signal;* $T_{AOX1}$*, native transcription termination from* AOX1 *gene of* P. pastoris*; Sh* ble *cassette, from* S. hindustanus ble *gene driving resistance to Zeocin.*

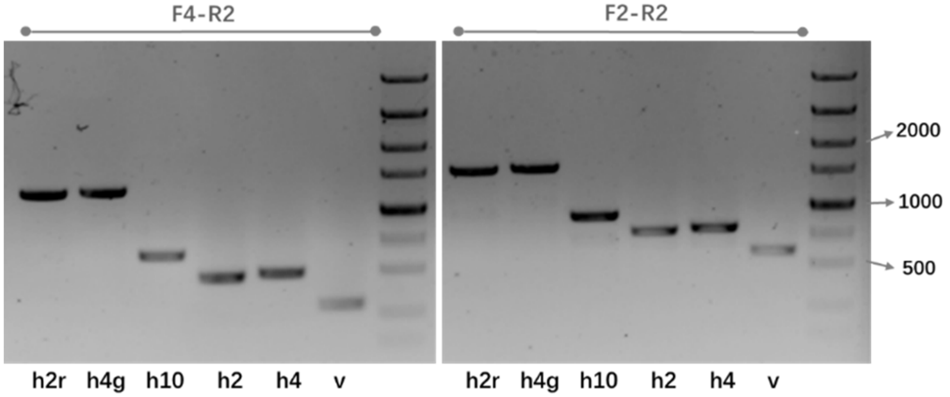


*Fig N PCR verification of mutant overexpressing* hfb*s (from* T. guizhouense *NJAU 4742) or fluorescently tagged* hfb*s in* P. pastoris*. h4 represents mutants harboring* hfb4*; h2 represents mutants harboring* hfb2*; h10 represents mutants harboring* hfb10*; h4 g represents mutants harboring* gfpuv*-fused* hfb4*; h2r represents mutants harboring mrfp-fused* hfb2*; v represents mutants transformed with the original vector pPICZαA without* hfb*s. A DL5000 DNA marker (Vazyme, China) was used in gel electrophoresis.*

## References

1. Cai F, Gao R, Zhao Z, Ding M, Jiang S, Yagtu C, et al. Evolutionary compromises in fungal fitness: hydrophobins can hinder the adverse dispersal of conidiospores and challenge their survival. ISME J. 2020. Epub 2020/07/08. doi: 10.1038/s41396-020-0709-0. PubMed PMID: 32632264.

2. Uzbas F, Sezerman U, Hartl L, Kubicek CP, Seiboth B. A homologous production system for Trichoderma reesei secreted proteins in a cellulase-free background. Appl Microbiol Biotechnol. 2012;93(4):1601-8. Epub 2011/11/15. doi: 10.1007/s00253-011-3674-8. PubMed PMID: 22080343; PubMed Central PMCID: PMCPMC3275749.
